# Monohydrazone Based G-Quadruplex Selective Ligands Induce DNA Damage and Genome Instability in Human Cancer Cells

**DOI:** 10.1021/acs.jmedchem.9b01866

**Published:** 2020-03-06

**Authors:** Jussara Amato, Giulia Miglietta, Rita Morigi, Nunzia Iaccarino, Alessandra Locatelli, Alberto Leoni, Ettore Novellino, Bruno Pagano, Giovanni Capranico, Antonio Randazzo

**Affiliations:** † Department of Pharmacy, University of Naples Federico II, via D. Montesano 49, 80131 Naples, Italy; ‡ Department of Pharmacy and Biotechnology, Alma Mater Studiorum - University of Bologna, 40126 Bologna, Italy

## Abstract

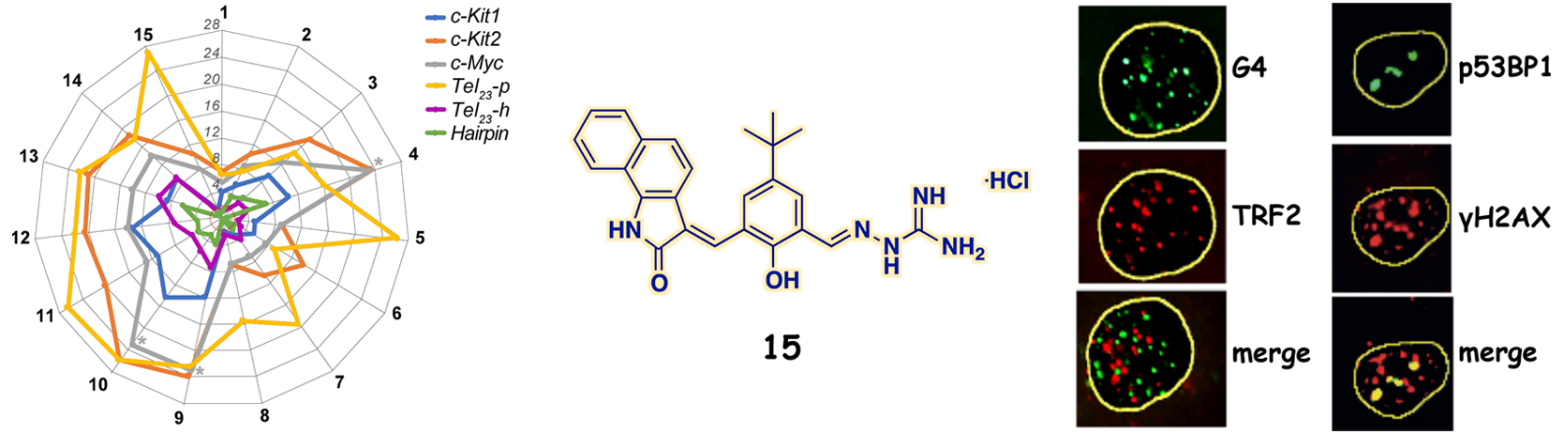

Targeting G-quadruplex structures is currently viewed as a promising anticancer strategy. Searching for potent and selective G-quadruplex binders, here we describe a small series of new monohydrazone derivatives designed as analogues of a lead which was proved to stabilize G-quadruplex structures and increase R loop levels in human cancer cells. To investigate the G-quadruplex binding properties of the new molecules, *in vitro* biophysical studies were performed employing both telomeric and oncogene promoter G-quadruplex-forming sequences. The obtained results allowed the identification of a highly selective G-quadruplex ligand that, when studied in human cancer cells, proved to be able to stabilize both G-quadruplexes and R loops and showed a potent cell killing activity associated with the formation of micronuclei, a clear sign of genome instability.

## Introduction

G-Quadruplexes (G4s) are noncanonical DNA secondary structures formed by G-rich sequences with important roles in the regulation of basic nuclear processes, including promoter activity,^1−4^ chromatin remodeling and replication,^5,6^ genome instability,^7−10^ and epigenetic alterations.^11,12^ G4s consist of four-stranded nucleic acid helical structures formed by the stacking of two or more guanine tetrads—cyclic planar arrays of four guanine bases held together by Hoogsteen hydrogen bonds—and stabilized by monovalent cations.^13^ In the past years, several specific G4 ligands have been shown to selectively stabilize G4 structures in living cells and trigger genome instability and cell killing, therefore supporting G4s as targets for anticancer drug developments.^3,14^ However, despite the high number of G4 binders reported so far, few have entered clinical trials and none have shown efficacy in cancer patients.^3,15^ Besides a general DNA damage response, the chemical stabilization of G4 structures can lead to recombination repair pathways and genomic rearrangements that can be suppressed by a specific G4-resolvase.^16^ The mechanisms are activated with different strengths depending on the chromatin localization and the G4 ligand chemical identity. Recently, some of us have demonstrated that the bis-guanylhydrazone derivative of diimidazo[1,2-*a*:1,2-*c*]pyrimidine (FG) and other G4 binders can induce DNA damage response and genome instability in cancer cells in an R loop-dependent manner.^17^ R loops are triple-stranded structures consisting of an RNA-DNA hybrid duplex and a displaced single-stranded DNA.^17^ They form co-transcriptionally at active genes^18^ and can lead to DNA damage and genome instability in yeast and mammalian cells.^19^ However, whether more-specific G4 binders can enhance the R loops causing genome instability in human cancer cells is not known.

**Figure 1:**
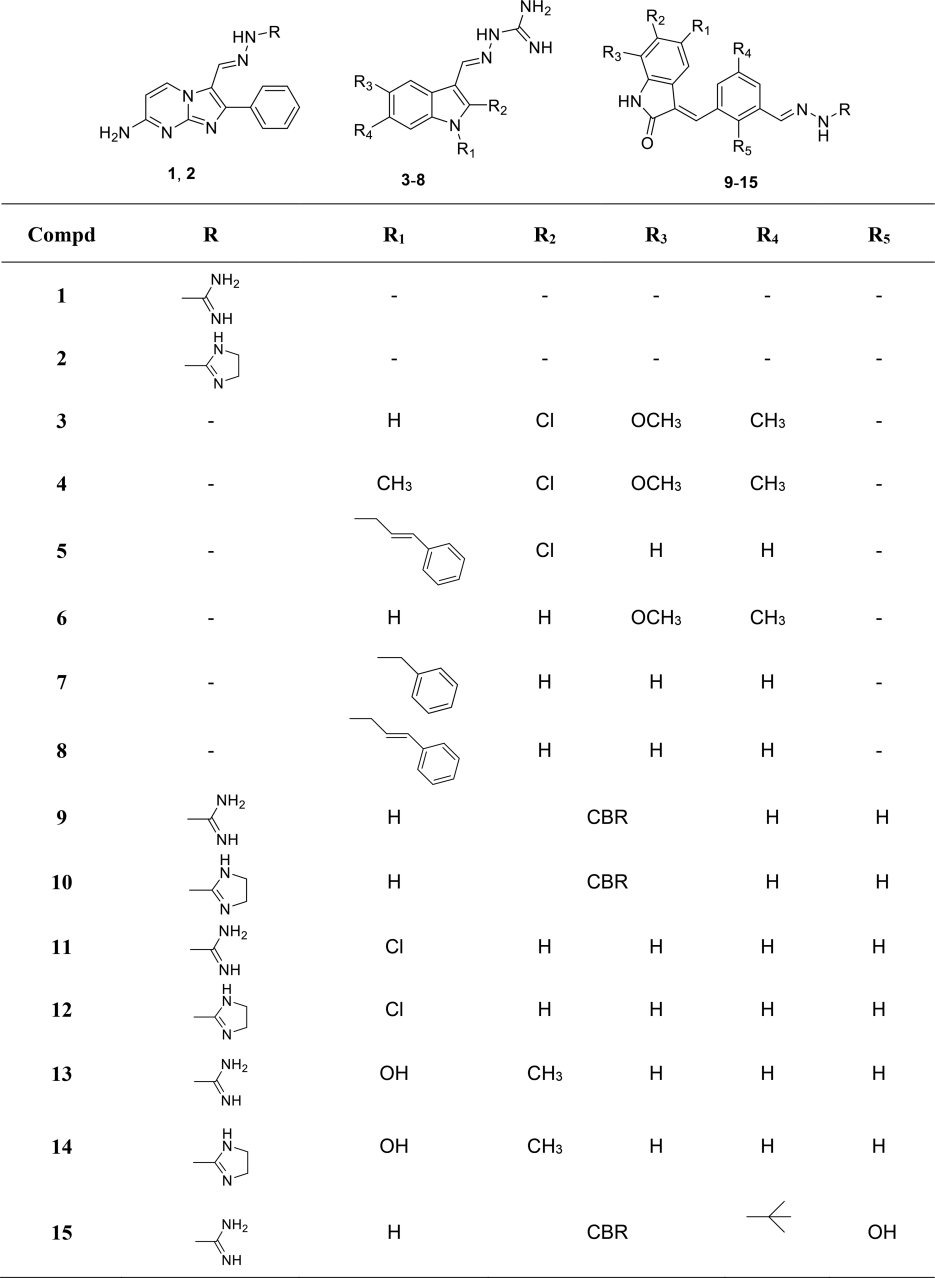
Chemical structures of the new hydrazone derivatives synthesized in this study; “CBR” stands for “condensed benzene ring”.

**Figure 2:**
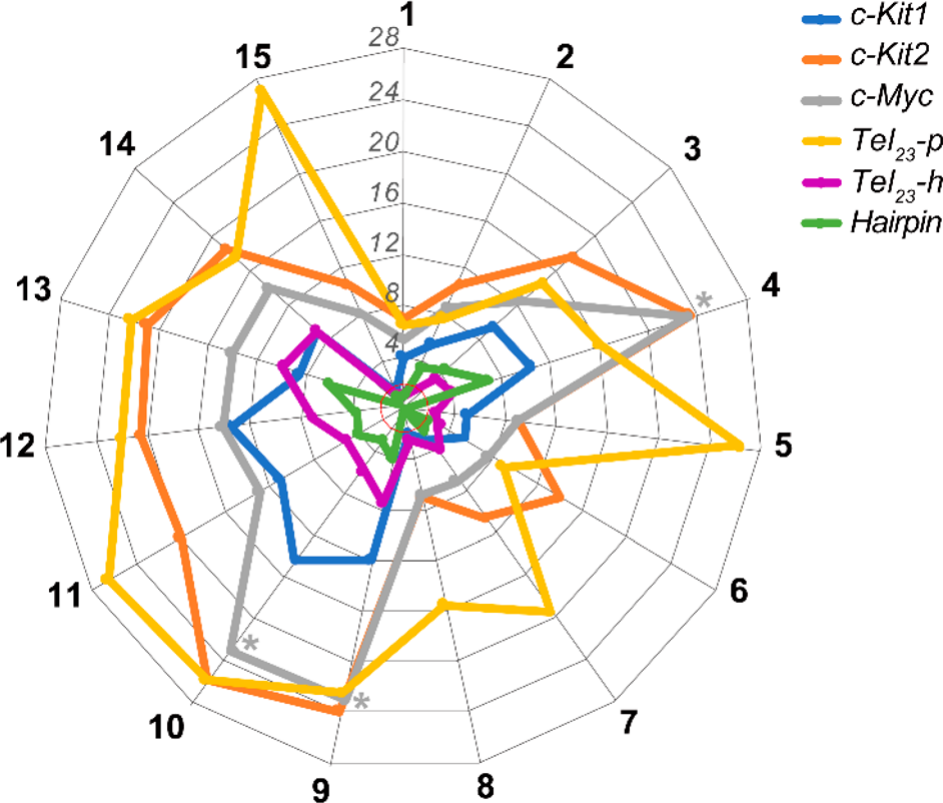
Spider chart showing the ligand-induced thermal stabilization of G4 and duplex DNAs measured by CD melting experiments. Δ*T*_m_ values are plotted for each sequence (Supporting Information (PDF), Table S1). The gray asterisks indicate Δ*T*_m_ values that were not accurately determinable, since these compounds increase the thermal stability of *c-Myc* G4 to values at which it was not possible to determine the *T*_m_.

Interestingly, certain hydrazone analogues of the lead compound FG turned out to be potent G4 ligands with high selectivity over duplex DNA and a preference for one G4 topology over others.^20^ In particular, decreasing the number of positively charged side chains on the molecule led to a significant benefit in terms of selectivity as the only monohydrazone of the series proved to be the most selective, being able to significantly stabilize *in vitro* only the *c-Myc* G4.^20^ Thus, inspired by our former results and with the aim of developing more potent and selective G4 binders, we designed new monohydrazone analogues in which the positively charged chain is represented by the iminoguanidine or a more rigid frame (Figure 1). These compounds are endowed with a simplified core as compared to the previous ones, specifically represented by an imidazopyrimidine (Figure 1, **1** and **2**) or an indole nucleus (Figure 1, **3**–**8**). Furthermore, since some of us also investigated the G4 binders formed by an aromatic core linked with two indolinone moieties and found that only one indolinone was involved in the interaction with the target,^21^ we additionally designed a number of monohydrazone analogues linked to a benzene ring which in turn is substituted with an indolinone unit (Figure 1, **9**–**15**).

Here, we therefore report on the synthesis and biophysical and biological characterizations of these new G4 binding compounds. The results show that the compounds have a higher selectivity of binding to certain G4s while being able to increase both G4 and R loop levels in human cancer cells and to trigger the formation of micronuclei, opening to investigations on the target specificity of genome instability induction.

## Results and Discussion

### Synthesis of Compounds **1**–**15**

**Scheme 1:**
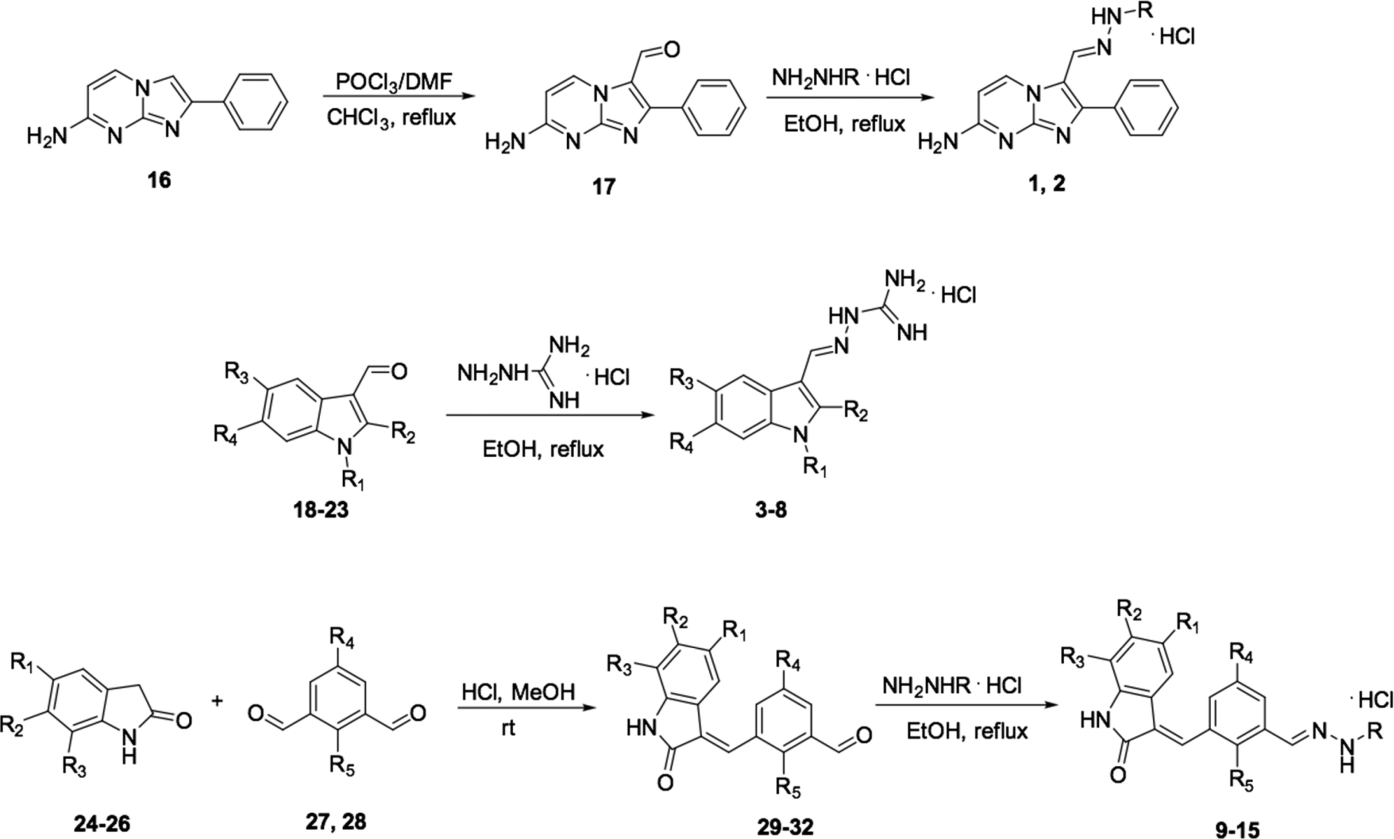
Synthetic Routes to Hydrazone Derivatives **1**–**15**

The designed hydrazones **1**–**15** (Figure 1 and Scheme 1) were prepared by the reaction between an aldehyde (**17**–**23**, **29**–**32**) and aminoguanidine hydrochloride or 2-hydrazino-2-imidazoline hydrobromide (Scheme 1) and were obtained as hydrochlorides or hydrobromides, as previously reported.^20^

The new starting aldehyde **17** was prepared by means of a Vilsmeier reaction on compound **16.** The monoformyl derivatives **29**–**32** were obtained by means of a Knoevenagel reaction between indolinones **24**–**26** and the appropriate bis-aldehyde **27** or **28**, performed at room temperature in order to promote the reaction of only one formyl group. The reaction led to the *E* isomer, as previously described,^22^ and was confirmed by performing an NOE experiment on compound **32**. Indeed, the irradiation of the methine bridge proton (7.73 ppm) gave NOE signals at 8.07 ppm (phenyl proton) and 1.34 ppm (*tert*-butyl group); no correlation was observed with the proton at position 4 of the indole system, as was expected in the case of the *E* configuration.

The starting compounds **16**, **18**–**24**, and **26** were prepared according to the literature (Experimental Section), whereas the 5-chloro-2-indolinone **25** and the bis-aldehydes **27** and **28** are commercially available (Table 1).

**Table 1:**
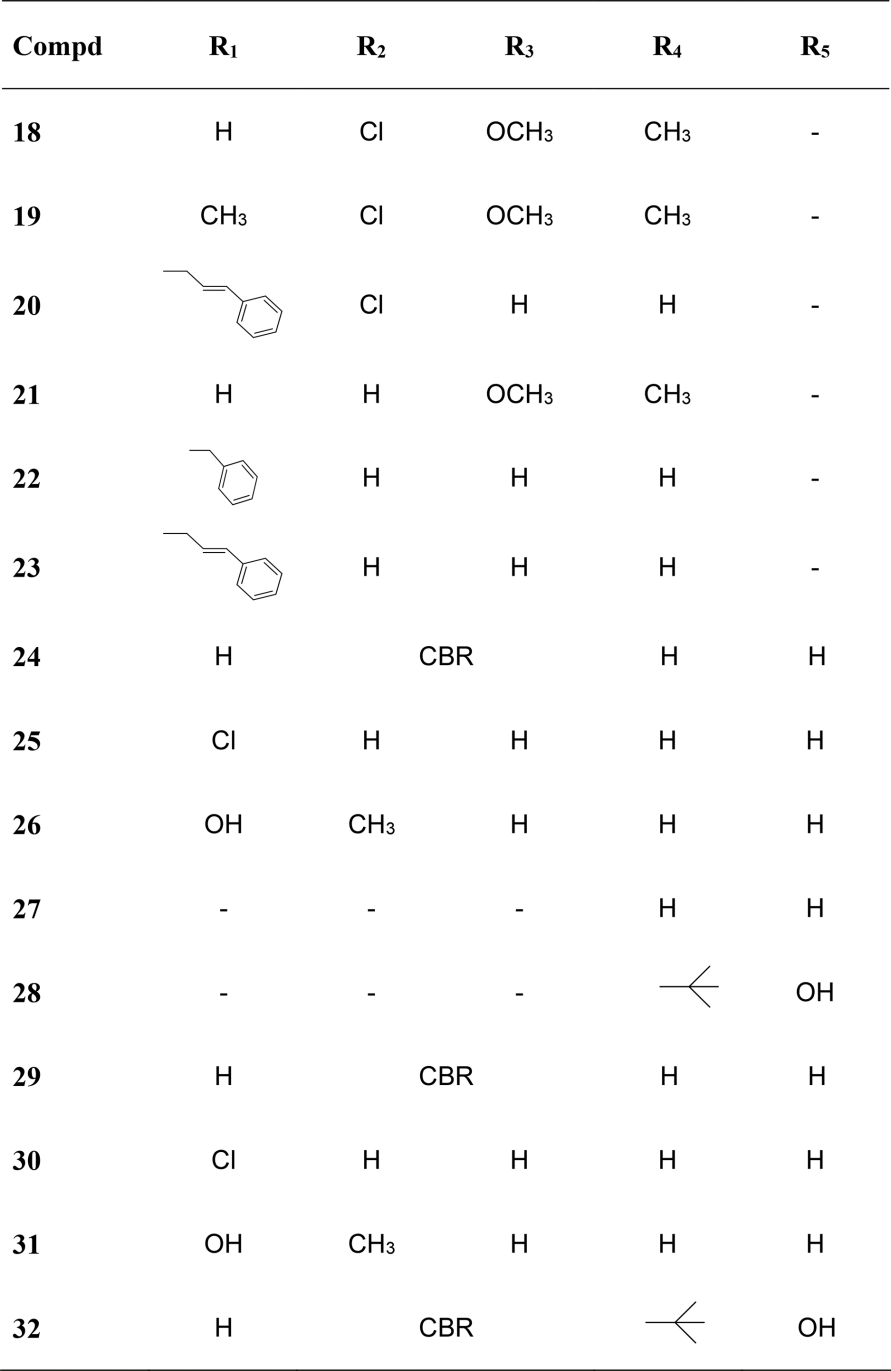
List of the Starting Compounds’ Substituents^a^

a “CBR” stands for “condensed benzene ring”.

### Circular Dichroism Studies

Compounds **1**–**15** were preliminarily screened for their ability to stabilize G4s by using a circular dichroism (CD) melting assay.^23^ Several diverse G4-forming sequences that are able to form parallel, antiparallel, and hybrid G4 structures were selected for this study. In particular, two G4-forming sequences from the nuclease hypersensitive region of the c-KIT promoter (*c-Kit1* and *c-Kit2*) and one from the c-MYC promoter (*c-Myc*) were used, along with a 23-mer truncation of the human telomeric sequence (*Tel*_*23*_). The latter can adopt different G4 topologies depending on the selected experimental conditions,^24^ as it folds into the so-called [3+1] hybrid conformation in diluted K^+^-containing solutions and into a parallel G4 conformation under the cell-mimicking molecular crowding conditions.^25^ Thus, we prepared two distinct *Tel*_*23*_ samples under different experimental conditions in order to promote either the hybrid or the parallel (hereafter referred to as *Tel*_*23*_*-h* or *Tel*_*23*_*-p*, respectively) G4 structure (Experimental Section).

First, CD spectra were collected to verify the folding of each G4 sample. *Tel*_*23*_*-p*, *c-Kit1*, *c-Kit2*, and *c-Myc* showed a positive band at 264 nm and a negative one around 240 nm (Supporting Information (PDF), Figure S1), which are characteristic bands of the parallel-stranded G4 topologies.^26,27^ However, *Tel*_*23*_*-p* exhibited a shoulder at about 290 nm in the CD spectrum, which implies that it is not a pure parallel G4 population under these conditions (Supporting Information (PDF), Figure S1). On the other hand, *Tel*_*23*_*-h* exhibited a positive band at 289 with a shoulder at around 268 nm and a weak negative band at 240 nm (Supporting Information (PDF), Figure S1), which are consistent with the presence of a hybrid structure as a major conformation.^28^

Additional CD experiments were performed in order to verify the capability of compounds **1**–**15** to alter the native folding topology of these G4s. DNA/ligand mixtures were prepared by adding ligands (10 mol equiv) to the native G4 structures for this purpose. No relevant differences in the CD profiles were detected for any of the analyzed G4s (Supporting Information (PDF), Figure S1), suggesting a general preservation of each G4 architecture upon ligand addition. The stabilizing properties of **1**–**15** were then evaluated by CD melting experiments measuring the ligand-induced change in the melting temperature (Δ*T*_m_) of the G4s. Results of these experiments, shown in Figure 2 and summarized in Table S1 (Supporting Information (PDF)), clearly indicate a good G4-stabilizing effect for all the investigated ligands except for compound **1**. In addition, most of them exhibited a preference for the parallel G4s over the hybrid *Tel*_*23*_*-h*. In particular, the highest thermal stabilization effects were observed for the *Tel*_*23*_*-p* and *c-Kit2* G4s.

Since the selectivity for the G4 structure over duplex DNA is another of the most important features for a lead G4-targeting compound, we also investigated their ability to stabilize a 20-mer hairpin-duplex DNA consisting of two self-complementary 8-mer sequences connected by a TTTT loop (hereafter referred to as *Hairpin*). CD spectra of such DNA are characterized by a positive band centered at ∼280 nm and a negative one at 250 nm (Supporting Information (PDF), Figure S2), which are characteristic values for a duplex DNA. These bands were not significantly altered upon the addition of compounds **1**–**15**. CD melting results recorded for *Hairpin* in the presence of **1**–**15** indicated a generally weak, but in some cases significant, increase in duplex stability (Figure 2 and Table S1, Supporting Information (PDF)). Consequently, all of the compounds shown to appreciably enhance the stability of *Hairpin* (Δ*T*_m_ ≥ 2 °C; i.e., **2**–**4**, **6**, **7**, **9**–**13**) were not further considered because of their modest selectivity.

Thus, since we were mainly interested in finding ligands with a great ability to selectively stabilize the G4 over the duplex and with a high degree of specificity for a G4 topology, further biophysical and biological investigations were conducted only on compound **15**. Indeed, this compound showed a preference for *Tel*_*23*_*-p*, *c-Kit2,* and *c-Myc* G4s (all forming parallel G4 conformations), while no significant thermal stabilization for the hybrid *Tel*_*23*_*-h* G4 was observed (Δ*T*_m_ < 2 °C).

### Fluorescence Intercalator Displacement (FID) Assay

**Figure 3:**
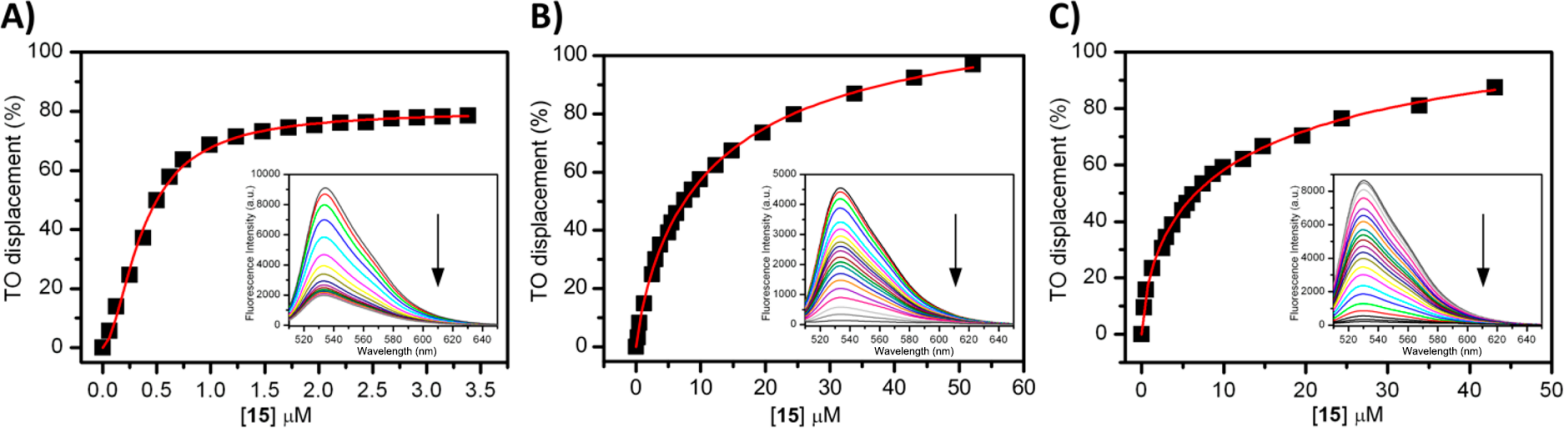
FID plots of TO-displacement titrations of compound **15** in the presence of (A) *Tel*_*23*_*-p* G4, (B) *c-Kit2* G4, and (C) *Hairpin* DNA.

To gain insight into the affinity of compound **15** for G4s, FID experiments were performed. Briefly, this assay is based on the displacement of an “on/off” fluorescent dye, i.e., thiazole orange (TO) dye, from the DNA upon the addition of increasing amounts of a candidate ligand.^29,30^ TO is almost nonfluorescent when free in solution; however, it becomes intensely fluorescent when bound to DNA. Hence, a ligand-induced TO displacement leads to a decrease in the fluorescence that can be monitored as a function of the ligand concentration, thus enabling the determination of their relative binding affinity to the structure under examination. Here, since *Tel*_*23*_*-p* and *c-Kit2* turned out to be the most thermally stabilized DNA structures among the investigated ones, TO displacement by **15** was investigated for these G4s (Figure 3). Dose-response curves were obtained by plotting the percentage of TO displacement versus the concentration of **15**, and the concentrations at which 50% displacement occurred (DC_50_) were calculated. For the parallel telomeric G4 a concentration of 0.48 (±0.02) μM of **15** was required to displace 50% TO, indicating a strong affinity of such a ligand for this G4 motif. On the other hand, a DC_50_ value of 7.80 (±0.03) μM was obtained for the interaction of **15** with *c-Kit2* G4, thus showing once again that this ligand has a clear preference for *Tel*_*23*_*-p* over *c-Kit2* G4. Indeed, these results agree with those obtained by CD melting, which show a ligand-induced thermal stabilization noticeably higher for *Tel*_*23*_*-p* G4 than *c-Kit2* G4 (Supporting Information (PDF), Table S1). Although the reason for this preference is not clear, we suppose that it can be attributed to a different binding mode of the ligand to the two G4s. Given the polycyclic aromatic nature of **15**, it is reasonable that it interacts via π–π stacking with the 5′ and/or 3′ G-tetrad(s) of a G4. This hypothesis is also in agreement with the greater ability of **15** to significantly stabilize the parallel G4 topology, in which the external G-tetrads are more prone to such a type of interaction, with respect to the hybrid [3+1] G4 conformation adopted by *Tel*_*23*_*-h*. However, the additional interactions of **15** with G4′ loops and grooves cannot be excluded, which could explain the difference in the binding affinity of **15** for the diverse parallel G4 structures. Indeed, although both *Tel*_*23*_*-p* and *c-Kit2* form parallel G4 structures, they differ in the length and base composition of the loops, with which the ligand most likely interacts by establishing additional electrostatic interactions. This hypothesis is also supported by the difference in the FID curves observed for the two G4s. In fact, the curve reaches 100% TO displacement for *c-Kit2* G4, suggesting that a strict competition occurs in this case with the probe. On the other hand, the probe is displaced by up to 80% in the case of *Tel*_*23*_*-p*, although its DC_50_ is lower as compared to *c-Kit2* G4. In the latter case, TO displacement might result from both direct and indirect competition. Finally, an FID assay was also performed by using the 20-mer hairpin-duplex DNA, from which a DC_50_ value of 8.57 (±0.05) μM was determined. This indicates that **15** also interacts with the duplex DNA, although with an affinity lower than that for the G4s.

### FRET-Melting Studies

**Figure 4:**
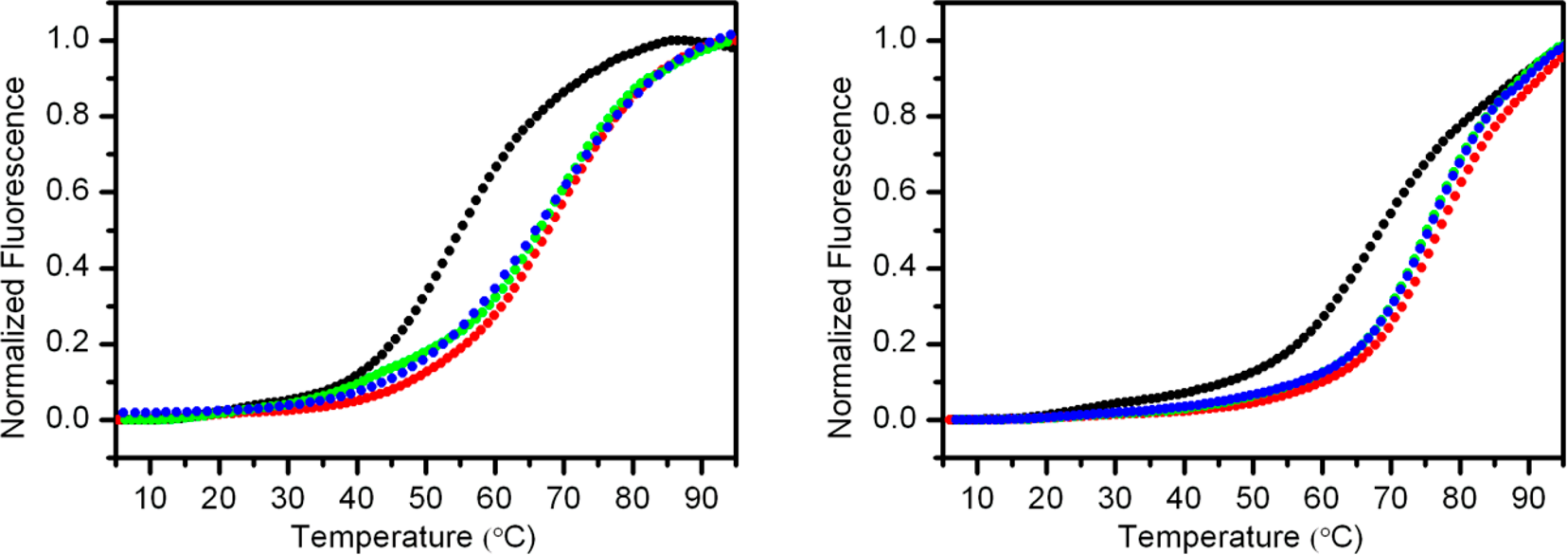
FRET-melting curves for *F-Tel*_*21*_*-T-p* (left panel) and *F-c-kit2-T* (right panel). Experiments were carried out by using 0.2 μM G4-forming oligonucleotides in the absence (black circles) and presence of **15** (2 μM, red circles). Experiments in the presence of **15** were also performed by adding a large excess of *ds12* duplex (5 and 10 μM, green and blue circles, respectively).

The G4 stabilizing properties of **15** were further investigated by the FRET (Förster resonance energy transfer) melting assay. This assay employs dual labeled oligonucleotides, with FAM (F) and TAMRA (T) being the most used FRET partners. When the oligonucleotide is folded, FAM and TAMRA are in close proximity; thus the fluorescence emission of FAM is minimal. During the G4 unfolding process, the relative distance and orientation of the probes significantly change and the large difference in the fluorescence emission of the folded and unfolded G4 is exploited to obtain well-resolved melting curves.^31^ Thus, the FRET-melting assay provides an assessment of the stabilization effect produced by ligand binding on a G4 structure by measuring the difference in the melting temperature (Δ*T*_m_) in the presence and absence of a ligand. Interestingly, since the targeted G4 is labeled, it is possible to evaluate the ligand selectivity by adding great amounts of unlabeled DNA competitors without interfering with the fluorescence signal. The *F-Tel*_*21*_*-T* and *F-c-Kit2-T* G4-forming oligonucleotides (0.2 μM single-stranded DNA) were used in this assay. *F-Tel*_*21*_*-T* G4 was prepared under experimental conditions so as to promote the formation of the corresponding parallel G4 conformation (referred to as *F-Tel*_*21*_*-T-p*), which was further confirmed by means of CD experiments (Supporting Information (PDF), Figure S3). FRET experiments were performed in the absence and presence of 10 mol equiv of **15** (2 μM), and the results are shown in Figure 4 and Table 2. It should be pointed out that the results of the FRET-melting experiments cannot be directly compared with those obtained from CD melting studies because of differences in DNA sequences and experimental conditions. In particular, the presence of FAM and TAMRA probes on a G4-forming sequence may affect its structural stability (the probes may stabilize or destabilize a G4 structure) as well as its interaction with ligands. Indeed, some artifacts may occur when compounds interact with the fluorescent probes rather than only with the DNA.^32^ Thus, we analyzed the FRET spectra of the labeled G4-forming DNA in the absence and presence of **15** (Supporting Information (PDF), Figure S4). In principle, compounds that interact with the fluorophores may affect the emission properties of the probes and decrease the intensity of the bands at 580 nm of TAMRA (if the G4 is structured) or at 522 nm of FAM (if the DNA is unstructured). Interestingly, we observed that **15** induced a decrease of the band intensity at 580 nm (for both *F-Tel*_*21*_*-T-p* and *F-c-kit2-T* G4s), suggesting that it may also interact with the fluorophores. However, results of the FRET experiments are in good qualitative agreement with those obtained from CD-melting studies, although lower Δ*T*_m_ values were observed. Again, this could be ascribed to the presence of the probes, which could partially hamper the ligand interaction with the external G-tetrads that, as previously hypothesized, may represent the binding site for **15**.

**Table 2:** Melting Temperature (*T*_m_) Values Obtained by FRET-Melting Experiments for *F-Tel*_*21*_*-T-p* and *F-c-kit2-T* in the Presence of **15** (10 mol equiv) without or with Large Excesses of *ds12* Duplex

	*T*_m_ (°C)			
G4	no ligand	**15**	**15** + 25-fold excess of duplex	**15** + 50-fold excess of duplex
*F-Tel*_*21*_*-T-p*	55.4 ± 0.2	68.6 ± 0.5	67.7 ± 0.5	68.1 ± 0.5
*F-c-kit2-T*	69.7 ± 0.3	77.9 ± 0.5	76.4 ± 0.5	76.5 ± 0.5

Moreover, to check the selectivity of **15** for G4s, competition FRET-melting experiments were carried out in the presence of a large excess of a duplex DNA (*ds12*, at either 5 or 10 μM) (Figure 4). The results clearly indicated that the G4-stabilizing effects of **15** are only slightly affected by the presence of the duplex competitor, thus indicating that this compound is a highly selective G4 ligand.

### Compound **15** Stabilizes G4 Structures in Living Cancer Cells

**Figure 5:**
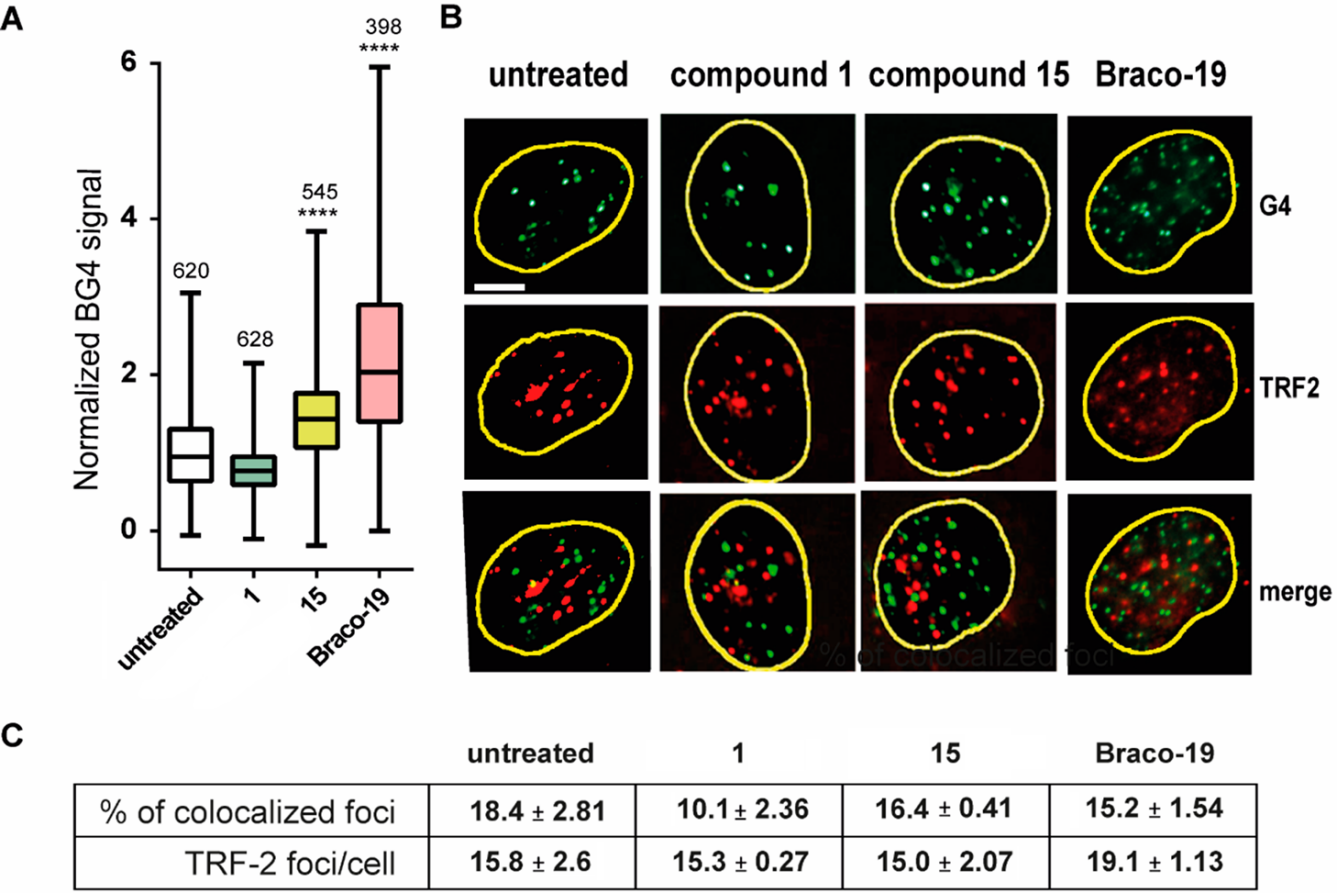
G-Quadruplex stabilization was induced by the newly synthesized G4 binders after a long treatment time in U2OS cancer cells. (A) Quantification of BG4 foci in U2OS cells after 24 h of treatment with **1** (10 μM), **15** (2 μM), and Braco-19 (10 μM). Values are the mean ± SE of at least three biological replicates. The significance has been evaluated by the Kologorov–Smirnov parametric test: * *p* < 0.05; **p > 0.01; ***p > 0.001; ****p < 0.0001 by GraphPad software. The numbers above the box plot indicate the cells nuclei analyzed. (**B**) Representative immunofluorescence images obtained by co-staining U2OS cells treated with the compounds (as previously described) along with BG4 and anti-TRF2 antibodies. The scale bar is 10 μm. (C) Quantification of TRF2 foci colocalizing with BG4. Values are the mean ± SE of two biological replicates.

We then investigated the cellular effects of compound **15** in comparison to **1**, which was chosen as a control because it shows a weak G4 binding activity *in vitro* (see above). First, we measured their cell killing potency and found that **15** was cytotoxic at the micromolar range, whereas **1** was essentially inactive in both the human U2OS osteosarcoma and the HeLa cervical carcinoma cell lines (Table 3). Next, we determined the ability of the two compounds to stabilize G4 structures in human U2OS cancer cells by means of immunofluorescence microscopy (IF) using the BG4 antibody, which selectively recognizes G4 structures.^33^ In this assay we also tested Braco-19, a well-known G4 binder and telomerase inhibitor,^34,35^ as a reference binder. As reported in Figure 5A and B, 24 h treatments with compound **15** (2 μΜ) or Braco-19 (10 μΜ) increased the number of G4 foci in U2OS cells, whereas compound **1** (10 μΜ) was not able to increase the number of G4 foci (BG4 total fluorescence quantification and BG4 foci counting raw data are reported in Supporting Information (PDF), Figure S5). The results thus indicate that compound **15** can likely bind and stabilize G4 structures in the nuclear chromatin of cancer cells without significantly changing the size of the BG4 foci (Supporting Information (PDF), Figure S6), whereas compound **1** is inactive. The levels of the increased number of G4 foci show that Braco-19 and **15** had comparable effects even though the latter was used at a 5-fold lower concentration (2 μΜ) than Braco-19 (10 μΜ) (Figure 5A). Thus, compound **15** is at least as effective as Braco-19 in stimulating G4 foci in U2OS cells.

**Table 3:** Cytotoxic Activity of Newly Synthesized G4 Binders in Human U2OS and HeLa Cell Lines after 1 and 24 h of Treatment Followed by 48 h of Recovery in a Drug-Free Medium^a^

	IC_50_ (μM)			
	U2OS cell line		HeLa cell line	
	1 h of treatment	24 h of treatment	1 h of treatment	24 h of treatment
compound **1**	>100	>50	>100	>50
compound **15**	12.51 ± 1.12	2.06 ± 1.23	3.94 ± 1.17	0.75 ± 1.19

a Concentrations killing 50% of cells (IC_50_) are shown as the mean ± SE of two independent experiments performed in triplicate.

Next, we wondered whether the number of G4 foci was preferentially increased by compound **15** at telomeres, as it showed a higher binding activity toward *Tel*_*23*_*-p* G4 than the other studied G4s. Thus, we co-stained U2OS cells with BG4 and an antibody against TRF2, a specific telomeric DNA binding factor. Surprisingly, BG4 foci scarcely overlapped with TRF2-specific IF signals in cells treated with compound **1**, compound **15**, or Braco-19 (Figure 5A and C); the percentage of colocalized foci is less than 20% both in the treated and in the untreated cells. The colocalization of foci in the absence of treatment is 15%, as previously reported by G. Biffi and co-authors,^33^ and it is not influenced by the treatment with compound **15** or Braco-19. Differently, BG4 and TRF2 colocalization for compound **1** decreases from 18.4 ± 2.81 in untreated cells to 10.1 ± 2.36 in treated cells (Figure 5A and C). These results thus show that the majority of G4s stabilized by either compound **15** or Braco-19 are not located at the telomeric regions in human cancer cells. Although Braco-19 was shown to be a telomerase inhibitor *in vitro*(34,35) and compound **15** had a preferential *in vitro* stabilization of telomeric G4s (Figure 2), they do not show a telomeric specificity in G4 stabilization in living cells.

### Compound **15** Induces an Increase of R Loop Levels in Human Cancer Cells

Recently, G4 formation has been shown to be closely linked to R loop structures in human cancer cells.^17^ G4s were previously shown to form in the displaced strand of an R loop, forming a G loop, depending on a high transcription rate and negative supercoiling.^36^ The presence of G4s and R loops in the same genomic fragment is consistent with the notion that both G4s and R loops are favored by the G-richness of the displaced DNA strand and the negative torsional tension, which are common features of active gene promoters.^37^ G loops were then demonstrated to form at the genomic sites of active transcription in human cancer cells upon treatment with well-known G4 binders, such as pyridostatin and FG.^17^ Interestingly, DNA damage and genome instability induced by G4 binders are dependent on R loop formation in cancer cells silenced for the BRCA2 gene.^17^

**Figure 6:**
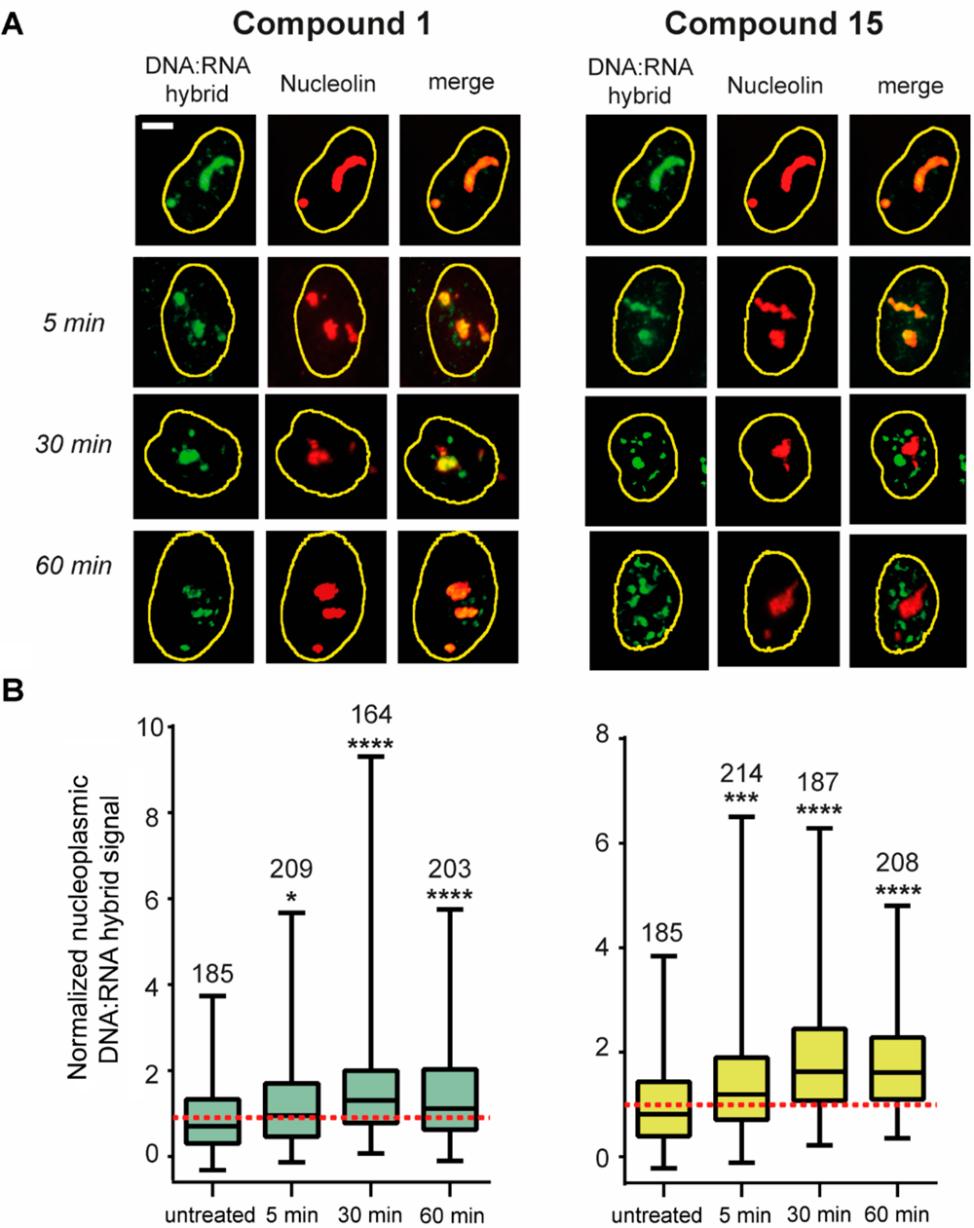
Increased DNA:RNA hybrid and G4 levels induced by G4 binders at short treatment times in U2OS cancer cells. (A) The kinetics of DNA:RNA hybrid induction upon treatment with **1** and **15** (10 μM) was determined by labeling the DNA:RNA hybrid and nucleolin with S9.6 (green) and AB22758 (Cell signaling) (red). The scale bar is 10 μm. (B) Nucleoplasmic DNA:RNA hybrid fluorescence quantification. Statistical significance has been calculated in comparison with untreated cells by the Kolmogorov–Smirnov parametric test: *p < 0.05; **p > 0.01; ***p > 0.001; ****p < 0.0001 by GraphPad software. The graphs show two biological replicates. The numbers above the box plot indicate the cell nuclei analyzed.

Thus, we wondered whether compound **15** can increase the number of R loops in living cells and investigated its effects on R loop levels after a short time of treatment (5, 30, and 60 min). In Figure 6, we report representative images of IF microscopy and nucleoplasmic DNA:RNA hybrid levels for compounds **1** and **15**. Nuclear DNA:RNA hybrid levels were detected with the S9.6 antibody, as previously described and validated.^17^ We quantify the DNA:RNA signal (green) restricted to the nucleoplasmic region by using the nucleolin staining (red) to visualize the nucleolus. The nucleolar S9.6 fluorescence is then subtracted from the total nuclear S9.6 fluorescence to measure only the R loop level in the nucleoplasmic region. Data are finally normalized to the mean of untreated cells. The time course data show that **15** can promote some increase of the nucleoplasmic hybrid signal after 30 min of treatment, which is maintained up to 60 min (Figure 6). R loop levels induced by compound **1** are lower than those of compound **15**. Therefore, the results show that **15** can affect nuclear R loops like other G4 binders reported previously.^17^ It is worth noting that compound **15** promotes an R loop increase at later times (30 min) than two other G4 binders, pyridostatin and FG (2 min).^17^ This may be due to a retarded pharmacokinetic of **15** in comparison to pyridostatin and FG. Consistently, compound **15** weakly stabilizes the G4s in the cells following short treatment times (Supporting Information (PDF), Figure S7).

### Compound **15** Can Trigger DNA Damage and Genome Instability in Human Cancer Cells

**Figure 7:**
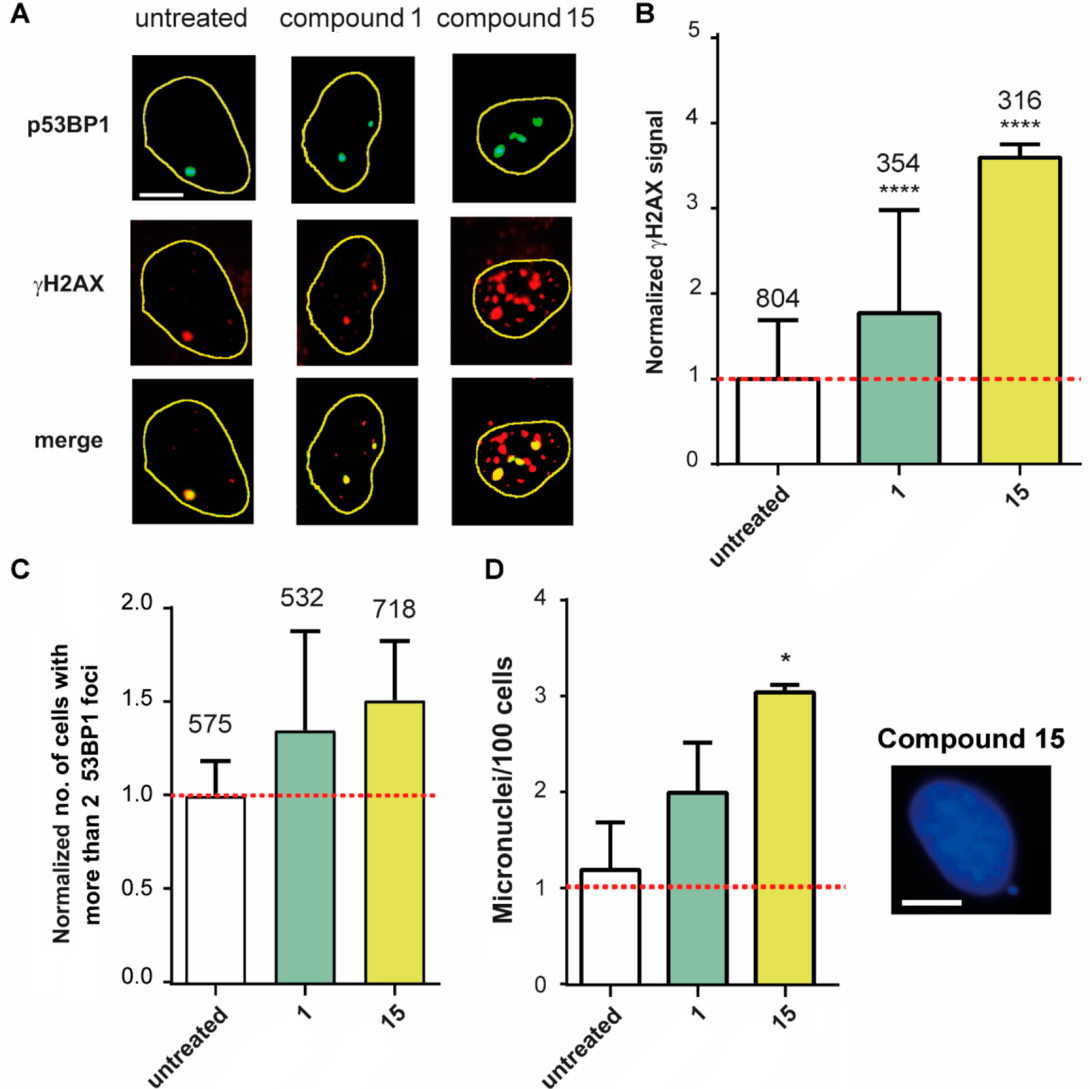
DNA damage and the formation of micronuclei by G4 binders in U2OS cancer cells. (A) Immunofluorescence microscopy images were obtained after 4 h of treatment with compounds **1** and **15** co-labeling p53BP1 (green) and γH2AX (red) foci in the U2OS cell line. (B) Graphs show the γH2AX signal quantification of three biological replicates. Asterisks indicate the statistical significance in comparison with untreated cells as calculated by the Kolmogorov–Smirnov parametric test: *p < 0.05; **p > 0.01; ***p > 0.001; ****p < 0.0001. Numbers above the box plot indicate the cell nuclei analyzed. (C) The graph shows the normalized number of cells with more than two 53BP1 foci of three biological replicates. Numbers above plot indicate cell nuclei analyzed. (D) Micronuclei induced by compounds **1** and **15** after 24 h of treatment in U2OS cells. Statistical significance was calculated by a multiple *t* test: *p < 0.05; **p > 0.01; ***p > 0.001; ****p < 0.0001. A representative image of a micronucleus induced by **15** is reported to the right. The scale bar is 10 μm.

Next, we investigated the biological consequences of the action of **15** in human cancer cells. As said above, compound **1** is only a weak G4 binder and does not elicit a cytotoxic response in cancer cell lines, whereas **15** is a good G4 binder and shows cell killing activity at micromolar ranges (Table 3). We then examined the induction of DNA damage and genome instability by the studied agents. DNA damage induced by compounds **1** and **15** after 4 h of treatment in U2OS cancer cells was determined by IF microscopy of specific DNA damage markers. As reported in Figure 7 (panels A and B), compound **15** causes a striking increase in the number of S139-phosphorylated histone H2AX (γH2AX) foci, a hallmark of a DNA double-strand break (DSB) and a DNA damage response activation, whereas compound **1** is less effective (see also Supporting Information (PDF), Figure S8). Histone H2AX is phosphorylated by several DNA damage checkpoint kinases at the genomic regions around the damage site in order to recruit specific DNA repair factors.^38^ In addition, we investigated a distinct DNA damage marker, 53BP1 (p53 binding protein 1), which is a DNA repair factor promoting DSB repair through the nonhomologous end-joining (NHEJ) pathway.^39^ We also performed co-staining experiments of phosphorylated 53BP1 and γH2AX in cells treated with the studied agents (Figure 7). The results show that compound **15** increases the number of both phosphorylated 53BP1 and γH2AX foci and that all phosphorylated 53BP1 foci colocalize with γH2AX foci. Thus, the results show that **15**, but much less **1**, can induce DSB after 4 h in human cancer cells with high efficiency.

We then wondered whether the DNA damage induced by compound **15** would lead to genome instability. To this end, we measured the formation of micronuclei that represents a hallmark of genome instability. Micronuclei are caused by error-prone DSB repair pathways and impaired chromosome segregation at mitosis.^40^ Microscope observations of U2OS cells treated with the studied agents showed that **15** increases the number of micronuclei after 24 h of treatment (Figure 7D). The number of micronuclei per 100 cells is increased 3-fold in **15**-treated cells relative to untreated cells, whereas compound **1** showed a lower increase. Thus, **15** can induce DSB that can be repaired by error-prone pathways leading to the generation of micronuclei in surviving cells.

## Conclusions

In this study, we focused on the investigation of a number of new hydrazone-containing compounds, designed as analogues of a promising lead^41^ which was recently demonstrated to stabilize G4s and simultaneously increase R loop levels in human cancer cells.^17^ Results of the biophysical characterization of their interaction with a number of G4 structures indicated compound **15** as the most promising of the series, since it showed a good selectivity for G4 over duplex DNA along with a distinct preference for the parallel G4 conformation adopted by the human telomeric sequence as opposed to other *in vitro* tested G4s. Despite the *in vitro* preference exhibited by **15** for the telomeric *Tel*_*23*_*-p* G4, biological results indicated that the majority of G4s stabilized by such compound are not located at the telomeric regions in human cancer cells. However, although unexpected this behavior is not unusual for G4 ligands, since Braco-19, which is a well-known telomerase inhibitor *in vitro,*(34,35) did not show telomeric specificity in G4 stabilization in cells. Indeed, the existence of *Tel*_*23*_*-p*-like G4 structures in a cell genome cannot be excluded, albeit it is not easy to predict their number. Interestingly, our results evidenced that compound **15** significantly stabilizes both G4s and R loops in cancer cells while triggering cell death and the formation of micronuclei, a clear sign of genome instability. As previously demonstrated, G4 binder-induced DNA damage can lead to either cell death or the formation of micronuclei in the surviving cells,^17^ and present data show that compound **15** induces DNA damage that prevalently results in cell killing activity (observed after 24 h of treatment and 48 h of recovery). Our findings raise the possibility that *in vivo* target selectivity of G4 binders may modulate the biological activity of the compound, either cell killing or the formation of micronuclei. In future investigations, it will be interesting to establish the biological role of the sequence selectivity of G4 binders.

## Experimental Section

### Chemical Synthesis

All the compounds prepared have a purity of at least 98% as determined by combustion analysis. The melting points are uncorrected. Reaction progress was monitored by TLC plates that were pre-coated with silica gel 60 F254 (Sigma-Aldrich, Milan, Italy) and visualized by UV (254 nm). Flash and gravity column chromatography were performed on a Kieselgel 60 (Merck); the eluent was a mixture of petroleum ether/acetone in various proportions. The ^1^H NMR and ^13^C NMR spectra were recorded on a Varian MR 400 MHz (ATB PFG probe) instrument (Agilent, Palo Alto, CA, USA); the chemical shift (referenced to the solvent signal) is expressed in δ (ppm). Multiplicities are quoted as s (singlet), d (doublet), t (triplet), and m (multiplet), with coupling constants defined as *J* given in Hz (abbreviations: pym = pyrimidine, ph = phenyl, ind = indole). Compounds were named relying on the naming algorithm developed by the CambridgeSoft Corporation (PerkinElmer, Milan, Italy) and used in the Chem-BioDraw Ultra 14.0 software (PerkinElmer, Milan, Italy). All solvents and reagents, unless otherwise stated, were supplied by Aldrich Chemical Co. Ltd. (Milan, Italy) and were used without further purification.

The 5-chloro-2-indolinone **25** and the bis-aldehydes **27** and **28** are commercially available. The following compounds were prepared according to the literature: **16**,^42^**18**,^43^**19**,^44^**20**,^45^**21**,^46^**22**,^47^**23**,^48^**24**,^49^ and **26**.^50^

### Synthesis of 7-Amino-2-phenylimidazo[1,2-*a*]pyrimidine-3-carbaldehyde (**17**)

The Vilsmeier reagent was prepared at 0–5 °C by dropping POCl_3_ (54 mmol) into a stirred solution of DMF (65 mmol) in CHCl_3_ (5 mL). Compound **16** (5 mmol) was suspended in CHCl_3_ (20 mL), and the mixture thus obtained was dropped into the Vilsmeier reagent while maintaining the stirring and cooling. The reaction mixture was kept for 3 h at room temperature and then under reflux for 5 h. The chloroform was removed under reduced pressure; the resulting oil was poured onto ice, and the suspension thus obtained was refluxed for 1 h. After cooling, the precipitate was collected by filtration and crystallized from ethanol to obtain aldehyde **17**. Yield: 95%. Mp: 255–257 °C. ^1^H NMR (DMSO*d*_6_): δ 6.53 (1H, d, pym, *J* = 7.4), 7.52 (5H, m, 3Hph + NH_2_), 7.83 (2H, m, ph), 9.19 (1H, d, pym, *J* = 7.4), 9.73 (1H, s, CHO). Anal. Calcd for C_13_H_10_N_4_O (MW 238.25): C, 65.54; H, 4.23; N, 23.52. Found: C, 65.51; H, 4.22; N, 23.51.

### General Procedure for the Synthesis of Aldehydes **29**–**32**

The appropriate bis-aldehyde (5.0 mmol) was dissolved in methanol (30 mL) and treated with the appropriate indolinone (5.0 mmol) and 37% HCl (2.0 mL). The reaction mixture was kept stirred at room temperature for 24 h. The precipitate thus obtained was collected by filtration, and the expected monoformyl derivative was isolated by flash chromatography. The eluent was petroleum ether/acetone (8:2).

#### (*E*)-3-((2-Oxo-1,2-dihydro-3*H*-benzo[*g*]indol-3-ylidene)methyl)benzaldehyde (**29**)

Yield: 27%. Mp: 241–243 °C. ^1^H NMR (DMSO*d*_6_): δ 7.52 (2H, m, ind), 7.59 (1H, d, ind, *J* = 8.2), 7.73 (1H, t, ph, *J* = 7.6), 7.91 (1H, d, ind, *J* = 8.2), 7.92 (1H, m, ind), 7.99 (1H, dt, ph, *J* = 7.6, *J* = 1.2), 8.02 (1H, s, CH), 8.13 (1H, m, ind), 8.76 (1H, dt, ph, *J* = 7.6, *J* = 1.2), 8.87 (1H, d, ph, *J* = 1.2), 10.08 (1H, s, CHO), 11.41 (1H, s, NH). Anal. Calcd for C_20_H_13_NO_2_ (MW 299.33): C, 80.25; H, 4.38; N, 4.68. Found: C, 80.28; H, 4.40; N, 4.70.

#### (*E*)-3-((5-Chloro-2-oxoindolin-3-ylidene)methyl)benzaldehyde (**30**)

Yield: 56%. Mp: 246–247 °C. ^1^H NMR (DMSO*d*_6_): δ 6.84 (1H, d, ind-7, *J* = 8.0), 7.27 (1H, dd, ind-6, *J* = 8.0, *J* = 2.0), 7.71 (1H, t, ph, *J* = 7.6), 7.88 (1H, d, ind-4, *J* = 2.0), 7.99 (1H, dt, ph, *J* = 7.6, *J* = 1.4), 8.06 (1H, s, CH), 8.66 (1H, dt, ph, *J* = 7.6, *J* = 1.4), 8.82 (1H, d, ph, *J* = 1.4), 10.06 (1H, s, CHO), 10.79 (1H, s, NH). Anal. Calcd for C_16_H_10_ClNO_2_ (MW 283.71): C, 67.74; H, 3.55; N, 4.94. Found: C, 67.71; H, 3.58; N, 4.91.

#### (*E*)-3-((5-Hydroxy-6-methyl-2-oxoindolin-3-ylidene)methyl)benzaldehyde (**31**)

Yield: 57%. Mp: 272–274 °C. ^1^H NMR (DMSO*d*_6_): δ 2.09 (3H, s, CH_3_), 6.59 (1H, s, ind), 6.99 (1H, s, ind), 7.53 (1H, s, CH), 7.74 (1H, t, ph, *J* = 7.6), 7.95 (1H, d, ph, *J* = 7.6), 7.98 (1H, d, ph, *J* = 7.6), 8.20 (1H, s, ph), 8.85 (1H, s, OH), 10.07 (1H, s, CHO), 10.25 (1H, s, NH). Anal. Calcd for C_17_H_13_NO_3_ (MW 279.29): C, 73.11; H, 4.69; N, 5.02. Found: C, 73.15; H, 4.71; N, 5.05.

#### (*E*)-5-(*tert*-Butyl)-2-hydroxy-3-((2-oxo-1,2-dihydro-3H-benzo[*g*]indol-3-ylidene)methyl)benzaldehyde (**32**)

Yield: 20%. Mp: 196–198 °C. ^1^H NMR (DMSO*d*_6_): δ 1.34 (9H, s, 3CH_3_), 7.39 (1H, d, ind, *J* = 8.8), 7.45 (1H, d, ind, *J* = 8.8), 7.53 (2H, m, ind), 7.73 (1H, s, CH), 7.87 (1H, m, ind), 7.95 (1H, d, ph, *J* = 2.4), 8.07 (1H, d, ph, *J* = 2.4), 8.14 (1H, m, ind), 10.18 (1H, s, CHO), 11.15 (1H, s, OH), 11.40 (1H, s, NH). Anal. Calcd for C_24_H_21_NO_3_ (MW 371.43): C, 77.61; H, 5.70; N, 3.77. Found: C, 77.58; H, 5.71; N, 3.75.

### General Procedure for the Synthesis of Hydrazones **1**–**15**

The appropriate aldehyde (5 mmol) was dissolved in ethanol and treated with 1 equiv of either aminoguanidine hydrogencarbonate suspended in ethanol and treated with hydrochloridric acid in order to achieve a solution (to obtain compounds **1**, **3**–**9**, **11**, **13**, **15**) or 2-hydrazino-2-imidazoline hydrobromide solubilized in ethanol (to obtain compounds **2**, **10**, **12**, **14**). The reaction mixture was refluxed for 5–30 h according to a TLC test. The solvent was partially evaporated under reduced pressure. The resulting precipitate was collected by filtration and crystallized from ethanol/ethyl ether.

#### (*E*)-2-((7-Amino-2-phenylimidazo[1,2-*a*]pyrimidin-3-yl)methylene)hydrazine-1-carboximidamide Hydrochloride (**1**)

Yield: 35%. Mp: 260–262 °C. ^1^H NMR (DMSO*d*_6_): δ 6.68 (1H, d, pym, *J* = 7.4), 7.57 (3H, m, ph), 7.70 (2H, m, ph), 7.96 (6H, broad, NH), 8.40 (1H, s, CH), 9.37 (1H, d, pym, *J* = 7.4), 12.04 (1H, s, NH). ^13^C NMR (DMSO*d*_6_): δ 102.30, 112.47, 128.96, 129.09, 129.65, 137.31, 137.95, 148.70, 154.62, 159.11, 160.53. Anal. Calcd for C_14_H_14_N_8_·HCl (MW 330.78): C, 50.84; H, 4.57; N, 33.88. Found: C, 50.81; H, 4.56; N, 33.86.

#### (*E*)-3-((2-(4,5-Dihydro-1*H*-imidazol-2-yl)hydrazono)methyl)-2-phenylimidazo[1,2-*a*]pyrimidin-7-amine Hydrobromide (**2**)

Yield: 35%. Mp: 280–282 °C. ^1^H NMR (DMSO*d*_6_): δ 3.78 (4H, s, 2CH_2_), 6.79 (1H, d, pym, *J* = 7.8), 7.63 (3H, m, ph), 7.68 (2H, m, ph), 8.37 (1H, s, CH), 8.43 (2H, s, NH_2_), 8.60 (2H, broad, NH), 9.38 (1H, d, pym, *J* = 7.8), 12.34 (1H, s, NH). ^13^C NMR (DMSO*d*_6_): δ 42.75, 103.40, 112.82, 129.21, 129.24, 130.51, 137.45, 138.34, 146.83, 156.76, 161.25. Anal. Calcd for C_16_H_16_N_8_·HBr (MW 401.27): C, 47.89; H, 4.27; N, 27.93. Found: C, 47.91; H, 4.27; N, 27.94.

#### (*E*)-2-((2-Chloro-5-methoxy-6-methyl-1*H*-indol-3-yl)methylene)hydrazine-1-carboximidamide Hydrochloride (**3**)

Yield: 45%. Mp: 120–123 °C. ^1^H NMR (DMSO*d*_6_): δ 2.24 (3H, s, CH_3_), 3.87 (3H, s, OCH_3_), 7.15 (1H, s, ind), 7.56 (1H, s, ind), 7.58 (4H, broad, NH), 8.33 (1H, s, CH), 11.89 (1H, s, NH), 12.44 (1H, s, NH). ^13^C NMR (DMSO*d*_6_): δ 16.80, 55.51, 101.88, 106.03, 112.57, 122.23, 122.96, 126.60, 129.43, 142.34, 153.61, 154.88. Anal. Calcd for C_12_H_14_ClN_5_O·HCl (MW 316.19): C, 45.58; H, 4.78; N, 22.15. Found: C, 45.61; H, 4.77; N, 22.18.

#### (*E*)-2-((2-Chloro-5-methoxy-1,6-dimethyl-1*H*-indol-3-yl)methylene)hydrazine-1-carboximidamide Hydrochloride (**4**)

Yield: 78%. Mp: 282–284 °C. ^1^H NMR (DMSO*d*_6_): 2.27 (3H, s, CH_3_), 3.74 (3H, s, CH_3_), 3.88 (3H, s, OCH_3_), 7.38 (1H, s, ind), 7.56 (4H, broad, NH), 7.59 (1H, s, ind), 8.35 (1H, s, CH), 11.91 (1H, s, NH). ^13^C NMR (DMSO*d*_6_): δ 16.93, 30.30, 55.54, 101.88, 105.84, 111.75, 121.37, 123.07, 128.45, 130.57, 142.33, 153.93, 154.91. Anal. Calcd for C_13_H_16_ClN_5_O·HCl (MW 330.21): C, 47.28; H, 5.19; N, 21.21. Found: C, 47.32; H, 5.23; N, 21.18.

#### (*E*)-2-((2-Chloro-1-cinnamyl-1*H*-indol-3-yl)methylene)hydrazine-1-carboximidamide Hydrochloride (**5**)

Yield: 55%. Mp: 128–130 °C. ^1^H NMR (DMSO*d*_6_): δ 5.11 (2H, d, CH_2_, *J* = 4.4), 6.40 (1H, dt, CH_2_CH=CH, *J* = 4.4, *J* = 16.0), 6.46 (1H, d, CH_2_CH=CH, *J* = 16.0), 7.28 (5H, m), 7.38 (2H, d, ph, *J* = 8.0), 7.56 (4H, broad, NH), 7.65 (1H, d, ind, *J* = 8.0), 8.37 (1H, d, ind, *J* = 8.0), 8.41 (1H, s, CH), 11.91 (1H, s, NH). ^13^C NMR (DMSO*d*_6_): δ 45.31, 106.70, 110.53, 122.01, 122.10, 123.03, 123.57, 123.85, 126.41, 127.93, 128.62, 129.69, 131.87, 135.47, 135.70, 142.06, 154.76. Anal. Calcd for C_19_H_18_ClN_5_·HCl (MW 387.10): C, 58.77; H, 4.93; N, 18.04. Found: C, 58.80; H, 4.91; N, 18.00.

#### (*E*)-2-((5-Methoxy-6-methyl-1*H*-indol-3-yl)methylene)hydrazine-1-carboximidamide Hydrochloride (**6**)

Yield: 82%. Mp: 180–182 °C. ^1^H NMR (DMSO*d*_6_): δ 2.25 (3H, s, CH_3_), 3.87 (3H, s, OCH_3_), 7.22 (1H, s, ind), 7.46 (4H, broad, NH), 7.58 (1H, s, ind), 7.76 (1H, s, ind), 8.32 (1H, s, CH), 11.54 (1H, s, NHind), 11.60 (1H, s, NH). ^13^C NMR (DMSO*d*_6_): δ 16.96, 55.50, 102.17, 110.35, 113.08, 122.45, 122.51, 130.96, 131.60, 145.27, 153.24, 154.81. Anal. Calcd for C_12_H_15_N_5_O·HCl (MW 281.74): C, 51.16; H, 5.72; N, 24.86. Found: C, 51.19; H, 5.74; N, 24.88.

#### (*E*)-2-((1-Benzyl-5-methoxy-6-methyl-1*H*-indol-3-yl)methylene)hydrazine-1-carboximidamide Hydrochloride (**7**)

Yield: 40%. Mp: 198–200 °C. ^1^H NMR (DMSO*d*_6_): δ 5.48 (2H, s, CH_2_), 7.17 (1H, t, ind, *J* = 8.0), 7.24 (1H, t, ind, *J* = 8.0), 7.28 (5H, m, ph), 7.40 (4H, broad, NH), 7.53 (1H, d, ind, *J* = 8.0), 8.08 (1H, s, ind), 8.33 (1H, d, ind, *J* = 8.0), 8.36 (1H, s, CH), 11.74 (1H, s, NH). ^13^C NMR (DMSO*d*_6_): δ 49.39, 110.28, 110.65, 121.09, 122.66, 123.00, 124.51, 127.15, 127.59, 128.63, 134.73, 136.92, 137.38, 144.24, 154.81. Anal. Calcd for C_17_H_17_N_5_·HCl (MW 327.81): C, 62.29; H, 5.53; N, 21.36. Found: C, 62.32; H, 5.49; N, 21.39.

#### (*E*)-2-((1-Cinnamyl-1*H*-indol-3-yl)methylene)hydrazine-1-carboximidamide Hydrochloride (**8**)

Yield: 35%. Mp: 85–87 °C. ^1^H NMR (DMSO*d*_6_): δ 5.03 (2H, d, CH_2_, *J* = 5.8), 6.49 (1H, dt, CH_2_CH=CH, *J* = 5.8, *J* = 15.6), 6.60 (1H, d, CH_2_CH=CH, *J* = 15.6), 7.23 (5H, m), 7.30 (4H, broad, NH), 7.41 (2H, d, ph, *J* = 7.6), 7.60 (1H, d, ind, *J* = 8.0), 7.99 (1H, s, ind), 8.34 (1H, d, ind, *J* = 8.0), 8.35 (1H, s, CH), 11.71 (1H, s, NH). ^13^C NMR (DMSO*d*_6_): δ 47.94, 110.16, 110.59, 121.08, 122.66, 122.95, 124.50, 124.97, 126.42, 127.88, 128.64, 132.29, 134.33, 135.92, 136.95, 144.31, 154.78. Anal. Calcd for C_19_H_19_N_5_·HCl (MW 353.14): C, 64.49; H, 5.70; N, 19.79. Found: C, 64.52; H, 5.68; N, 19.81.

#### 2-((*E*)-3-((*E*)-(2-Oxo-1,2-dihydro-3H-benzo[*g*]indol-3-ylidene)methyl)benzylidene)hydrazine-1-carboximidamide Hydrochloride (**9**)

Yield: 43%. Mp: 265–267 °C. ^1^H NMR (DMSO*d*_6_): δ 7.39 (1H, d, ind, *J* = 8.4), 7.52 (2H, m, ind), 7.63 (1H, t, ph, *J* = 7.6), 7.73 (1H, s, ph), 7.82 (4H, broad, NH), 7.83 (1H, d, ph, *J* = 7.6), 7.84 (2H, m, ind), 7.98 (1H, d, ph, *J* = 7.6), 8.15 (1H, m, ind), 8.26 (2H, s, 2CH), 11.42 (1H, s, NHind), 11.99 (1H, s, NH). ^13^C NMR (DMSO*d*_6_): δ 115.12, 119.35, 120.01, 120.67, 122.69, 126.16, 127.21, 128.34, 128.40, 129.05, 129.14, 130.71, 134.05, 134.11, 134.65, 135.15, 140.37, 146.09, 150.59, 155.44, 169.58. Anal. Calcd for C_21_H_17_N_5_O·HCl (MW 391.86): C, 64.37; H, 4.63; N, 17.87. Found: C, 64.35; H, 4.60; N, 17.85.

#### 3-((*E*)-3-((*E*)-(2-(4,5-Dihydro-1*H*-imidazol-2-yl)hydrazineylidene)methyl)benzylidene)-1,3-dihydro-2H-benzo[*g*]indol-2-one Hydrobromide (**10**)

Yield: 38%. Mp: 278–280 °C. ^1^H NMR (DMSO*d*_6_): δ 3.76 (4H, s, 2CH_2_), 7.53 (2H, m, ind), 7.58 (1H, d, ind, *J* = 8.8), 7.60 (1H, t, ph, *J* = 7.6), 7.87 (1H, d, ind, *J* = 8.8), 7.91 (1H, m, ind), 7.92 (1H, s, ph), 8.01 (1H, d, ph, *J* = 7.6), 8.13 (1H, m, ind), 8.27 (1H, s, CH), 8.55 (1H, d, ph, *J* = 7.6), 8.61 (1H, s, CH), 8.72 (2H, broad, NH), 11.40 (1H, s, NHind), 12.34 (1H, s, NH). ^13^C NMR (DMSO*d*_6_): δ 42.77, 118.03, 118.86, 119.13, 120.97, 122.32, 126.03, 126.59, 128.11, 128.48, 128.57, 128.67, 131.61, 133.31, 133.80, 134.64, 135.80, 137.51, 147.67, 157.88, 167.89. Anal. Calcd for C_23_H_19_N_5_O·HBr (MW 462.35): C, 59.75; H, 4.36; N, 15.15. Found: C, 59.77; H, 4.33; N, 15.18.

#### 2-((*E*)-3-(((*E*)-5-Chloro-2-oxoindolin-3-ylidene)methyl)benzylidene)hydrazine-1-carboximidamide Hydrochloride (**11**)

Yield: 36%. Mp: 193–195 °C. ^1^H NMR (DMSO*d*_6_): δ 6.91 (1H, d, ind, *J* = 8.2), 7.30 (1H, d, ind, *J* = 8.2), 7.33 (1H, s, ind), 7.64 (1H, t, ph, *J* = 7.6), 7.74 (1H, s, ph), 7.77 (1H, d, ph, *J* = 7.6), 7.80 (4H, broad, NH), 7.97 (1H, d, ph, *J* = 7.6), 8.24 (2H, s, 2CH), 10.82 (1H, s, NHind), 11.90 (1H, s, NH). ^13^C NMR (DMSO*d*_6_): δ 111.59, 121.99, 122.39, 124.97, 127.62, 127.99, 129.10, 129.20, 129.82, 130.49, 134.22, 134.65, 136.86, 141.82, 145.82, 155.48, 168.09. Anal. Calcd for C_17_H_14_ClN_5_O·HCl (MW 376.24): C, 54.27; H, 4.02; N, 18.61. Found: C, 54.30; H, 3.98; N, 18.63.

#### 5-Chloro-3-((*E*)-3-((*E*)-(2-(4,5-dihydro-1*H*-imidazol-2-yl)hydrazineylidene)methyl)benzylidene)indolin-2-one Hydrobromide (**12**)

Yield: 28%. Mp: 162–165 °C. ^1^H NMR (DMSO*d*_6_): δ 3.75 (4H, s, 2CH_2_), 6.91 (1H, d, ind, *J* = 8.0), 7.30 (2H, m, ind), 7.66 (1H, t, ph, *J* = 7.6), 7.75 (1H, s, ph), 7.79 (1H, d, ph, *J* = 7.6), 7.95 (1H, d, ph, *J* = 7.6), 8.16 (1H, s, CH), 8.25 (1H, s, CH), 8.70 (2H, broad, NH), 10.81 (1H, s, NHind), 12.36 (1H, s, NH). ^13^C NMR (DMSO*d*_6_): δ 42.71, 111.63, 121.99, 122.36, 125.00, 127.71, 128.00, 128.90, 129.31, 129.89, 130.61, 134.10, 134.73, 136.76, 141.83, 147.05, 157.97, 168.07. Anal. Calcd for C_19_H_16_ClN_5_O·HBr (MW 446.73): C, 51.08; H, 3.84; N, 15.68. Found: C, 51.11; H, 3.81; N, 15.71.

#### 2-((*E*)-3-(((*E*)-5-Hydroxy-6-methyl-2-oxoindolin-3-ylidene)methyl)benzylidene)hydrazine-1-carboximidamide Hydrochloride (**13**)

Yield: 25%. Mp: 223–225 °C. ^1^H NMR (DMSO*d*_6_): δ 2.09 (3H, s, CH_3_), 6.59 (1H, s, ind), 7.05 (1H, s, ind), 7.49 (1H, s, CH), 7.59 (1H, t, ph, *J* = 7.6), 7.74 (1H, d, ph, *J* = 7.6), 7.78 (4H, broad, NH), 7.98 (1H, d, ph, *J* = 7.6), 8.12 (1H, s, ph), 8.23 (1H, s, CH), 8.88 (1H, s, OH), 10.25 (1H, s, NHind), 11.79 (1H, s, NH). ^13^C NMR (DMSO*d*_6_): δ 16.59, 109.44, 111.99, 118.71, 126.78, 128.10, 128.48, 129.13, 130.57, 132.45, 132.94, 134.07, 135.22, 135.61, 146.30, 149.79, 155.44, 168.80. Anal. Calcd for C_18_H_17_N_5_O_2_·HCl (MW 371.82): C, 58.14; H, 4.88; N, 18.84. Found: C, 58.17; H, 4.91; N, 18.87.

#### 3-((*E*)-3-((*E*)-(2-(4,5-Dihydro-1H-imidazol-2-yl)hydrazineylidene)methyl)benzylidene)-5-hydroxy-6-methylindolin-2-one Hydrobromide (**14**)

Yield: 23%. Mp: 200–202 °C. ^1^H NMR (DMSO*d*_6_): δ 2.09 (3H, s, CH_3_), 3.74 (4H, s, 2CH_2_), 6.59 (1H, s, ind), 7.02 (1H, s, ind), 7.49 (1H, s, CH), 7.61 (1H, t, ph, *J* = 7.6), 7.75 (1H, d, ph, *J* = 7.6), 7.93 (1H, d, ph, *J* = 7.6), 8.07 (1H, s, ph), 8.23 (1H, s, CH), 8.65 (2H, broad, NH), 8.83 (1H, s, OH), 10.24 (1H, s, NHind), 12.33 (1H, s, NH). ^13^C NMR (DMSO*d*_6_): δ 16.58, 42.73, 109.42, 112.00, 118.69, 126.83, 127.95, 128.43, 129.23, 130.64, 132.81, 133.92, 135.29, 135.63, 147.46, 149.76, 157.92, 168.75. Calcd for C_20_H_19_N_5_O_2_·HBr (MW 442.31): C, 54.31; H, 4.56; N, 15.83. Found: C, 54.28; H, 4.60; N, 15.86.

#### 2-((*E*)-5-(*tert*-Butyl)-2-hydroxy-3-((*E*)-(2-oxo-1,2-dihydro-3H-benzo[*g*]indol-3-ylidene)methyl)benzylidene)hydrazine-1-carboximidamide Hydrochloride (**15**)

Yield: 25%. Mp: 230–232 °C. ^1^H NMR (DMSO*d*_6_): δ 1.33 (9H, s, 3CH_3_), 7.39 (1H, d, ind, *J* = 8.4), 7.50 (1H, d, ind, *J* = 8.4), 7.52 (2H, m, ind), 7.76 (4H, broad, NH), 7.79 (1H, s, CH), 7.90 (3H, m, 2ph+ind), 8.15 (1H, m, ind), 8.53 (1H, s, CH), 9.86 (1H, s, OH), 11.38 (1H, s, NHind), 11.88 (1H, s, NH). ^13^C NMR (DMSO*d*_6_): δ 31.08, 34.19, 115.34, 119.38, 120.04, 120.27, 122.62, 123.02, 126.09, 127.03, 127.40, 128.42, 129.20, 131.46, 133.89, 140.03, 141.94, 146.42, 152.84, 155.04, 169.57. Anal. Calcd for C_25_H_25_N_5_O_2_·HCl (MW 463.96): C, 64.72; H, 5.65; N, 15.09. Found: C, 64.75; H, 5.66; N, 15.13.

### Oligonucleotide Synthesis and Sample Preparation

DNA sequences were synthesized on an ABI 394 DNA/RNA synthesizer (Applied Biosystem) using standard β-cyanoethyl phosphoramidite solid phase chemistry at the 5 μmol synthesis scale. After detachment from the support and deprotection using a concentrated ammonia aqueous solution at 55 °C for 12 h, DNAs were purified by high-performance liquid chromatography (HPLC) on a Nucleogel SAX column (Macherey-Nagel, 1000-8/46), as previously reported.^51^ The fractions of the oligomers were collected and successively desalted by Sep-pak cartridges (C-18). The isolated oligonucleotides were proved to be >98% pure by NMR. In particular, the following oligonucleotides were synthesized and used for the CD experiments: the *c-Kit1* and *c-Kit2* sequences [d(AGGGAGGGCGCTGGGAGGAGGG) and d(CGGGCGGGCGCTAGGGAGGGT), respectively] from the c-KIT oncogene promoter, the c-MYC promoter sequence d(TGAGGGTGGGTAGGGTGGGTAA) (*c-Myc*), the 23-mer truncation of the human telomeric sequence d[TTAGG-G(TTAG-GG)_3_TT] (*Tel*_*23*_), the 20-mer hairpin duplex-forming sequence d(CGAATTCGTTTTCGAATTCG) (*Hairpin*), and the self-complementary duplex-forming Dickerson dodecamer d(CGCGAATTCGCG) (*ds12*). The oligonucleotide concentration was measured by UV adsorption at 90 °C using the appropriate molar extinction coefficient values, ε (λ = 260 nm), calculated by the nearest neighbor model.^52^ Samples were prepared in 20 mM potassium phosphate buffer (pH 7.0) containing 5 mM KCl and annealed by heating at 90 °C for 5 min, followed by a slow cooling to room temperature overnight. Parallel arrangement of the telomeric sequence (*Tel*_*23*_*-p*) was prepared by performing the annealing at high DNA concentration conditions, as previously described.^51^ After annealing, the concentrated DNA solution was kept at 4 °C for 24 h before dilution to the desired concentration. CD spectral variations were monitored over time to verify that the dilution did not alter the species in solution.

### CD Experiments

Circular dichroism (CD) experiments were performed on a Jasco J-815 spectropolarimeter equipped with a PTC-423*S*/15 Peltier temperature controller. All of the spectra were recorded at 20 °C in the wavelength range of 230–360 nm and averaged over three scans. The scan rate was set to 100 nm/min, with a 1 s response time and 1 nm bandwidth. The buffer baseline was subtracted from each spectrum. For CD experiments, 15 μM G4s and 30 μM *Hairpin* were used unless otherwise stated. CD spectra of DNA/ligand mixtures were obtained by adding 10 mol equiv of ligands (stock solutions of ligands were 10 mM in DMSO). CD melting experiments were carried out in the 20–100 °C temperature range at a 1 °C/min heating rate by following changes of the CD signal at the wavelengths of the maximum CD intensity (i.e., 264 nm for *c-Kit1*, *c-Kit2*, *c-Myc*, and *Tel*_*23*_*-p*; 287 nm for *Tel*_*23*_*-h*; 280 nm for *Hairpin* DNA). CD melting experiments were performed in the absence and presence of ligands (10 mol equiv) added to the folded DNA structures. The melting temperatures (*T*_m_) were determined from a curve fit using Origin 7.0 software. Δ*T*_m_ values were determined as the difference in the melting temperature of DNA structures with and without ligands. All experiments were performed in triplicate, and the values reported are the average of the three measurements.

### FID Assay

FID experiments were performed at 20 °C on a FP-8300 spectrofluorimeter (Jasco) equipped with a Peltier temperature controller accessory (Jasco PCT-818). A sealed quartz cuvette with a path length of 1 cm was used. The assay was designed as follows: 0.25 μM pre-folded DNA target was mixed with thiazole orange (0.50 μM for G4s, 0.75 μM for double-stranded DNA).^53^ Each ligand addition (from 0.5 to 10 equiv) was followed by a 3 min equilibration time, after which the fluorescence spectrum was recorded. Measurements were made with excitation at 495 nm and emission from 510 to 650 nm, with both excitation and emission slits set at 5 nm. The percentage of displacement was calculated as follows: TO displacement (%) = 100 – [(*F*/*F*_0_) × 100], where *F* stands for the intensity of the fluorescence emission signal at 543 nm of TO bound to the DNA after each ligand addition and *F*_0_ without added ligand. The percentage of displacement was then plotted as a function of the concentration of added ligand. DC_50_ values were designed as the required concentration to displace 50% TO from each investigated DNA.

### FRET-Melting Experiments

FRET experiments were carried out on a FP-8300 spectrofluorometer (Jasco) equipped with a Peltier temperature controller system (Jasco PCT-818). The experiments were performed by using the dual labeled G-quadruplex-forming sequences *F-Tel*_*21*_*-T* (FAM-^5′^GGGTTAGGGTTAGGGTTAGGG^3′^-TAMRA) and *F-c-kit2-T* (FAM-^5′^CGGGCGGGCGCTAGGGAGGGT^3′^-TAMRA).^53^*F-Tel*_*21*_*-T* and *F-c-kit2-T* were purchased from Biomers (Germany) and used without further purification. Oligonucleotides were dissolved in 20 mM potassium phosphate buffer (pH 7.0) containing 5 mM KCl. A parallel arrangement of *F-Tel*_*21*_*-T* (referred to as *F-Tel*_*21*_*-T-p*) was prepared at a high DNA concentration (10 mM) as reported above, while *F-c-kit2-T* was prepared at 1 mM. The samples were annealed by heating to 90 °C for 5 min, followed by cooling to room temperature overnight and storage at 4 °C for 24 h before the acquisition of data. Experiments were performed in sealed quartz cuvettes with a path length of 1 cm by using 0.2 μM G4-forming oligonucleotides, the ligand at 2 μM, and the double-stranded DNA competitor (*ds12*) at 0, 5, and 10 μM final concentrations. In addition, a blank with no compound or competitor was also analyzed. Measurements were made with excitation at 492 nm and detection at 522 nm with both excitation and emission slits set at 5 nm. FRET melting was monitored at 1 °C/min over the range 5–95 °C. Emission of FAM was normalized between 0 and 1. Final analysis of the data was carried out using Origin 7.0 software.

### Cell Lines and Drugs

The human osteosarcoma cell lines U2OS and HeLa were grown in monolayer cultures in Dulbecco’s Modified Eagle Medium (DMEM) supplemented with 10% fetal bovine serum (FBS) (Gibco) and 1% l-glutamine (Gibco) in a humidified incubator at 37 °C and 5% CO_2_. Cell line identity is routinely checked by genotyping (BMR genomics). Compounds used in this study were dissolved in dimethyl sulfoxide (Sigma-Aldrich #472301) at 10 mM concentration, stored in aliquots at −20 °C, and diluted at the correct concentration for treatment immediately prior to use.

### MTT Cell Proliferation Assay

U2OS and HeLa cells (25 × 10^4^) were seeded in a 24-well plate. Twenty-four hours after seeding, the cells were treated with the compound at the indicated concentration. After 1 or 24 h of treatment, agents were removed and the cells were further cultured in a completely drug-free medium for 48 h. A thiazolyl blue tetrazolium bromide (MTT) (Merck #2128) solution was then added to each well and incubated for 1 h at 37 °C. Next, the medium was removed and 300 μL of dimethyl sulfoxide was added and incubated for 1 h at room temperature. Then, 100 μL of the solution was put in a 96-well plate, and absorbance at 595 nm was measured using a multiplate reader. The linear regression parameters were determined to calculate the IC_50_ (GraphPad Prism 4.0, GraphPad Software Inc.).

### Immunofluorescence Microscopy

U2OS cells (2 × 10^5^) were seeded in a 35 mm dish on coverslips.

#### Visualization of Micronuclei

U2OS cells were fixed with 4% paraformaldehyde for 15 min, permeabilized with 0.5% Triton X-100 for 15 min at room temperature, then washed three times in PBS, and incubated with 2 μg/μL DAPI (Merck #D9542) for 20 min. The cover glasses were mounted with Mowiol 488 (Merck #81381).

#### G4 and TRF2 Co-staining Immunofluorescence

Cells were treated with 10 μM compound **1** and Braco-19 and 2 μM compound **15** for 24 h, or else as indicated. Cells were pre-fixed with a solution of 50% DMEM and 50% cold methanol/acetic acid (3:1) and then fixed with methanol/acetic acid (3:1) for 10 min at room temperature. Cells were permeabilized in 0.1% Triton X-100 (MerckMillipore) and blocked in 2% milk/PBS for 1 h at room temperature. Immunofluorescence was performed as described previously.^17^ The antibodies were BG4 (for G4 structures), anti-FLAG (Cell Signaling Technology #2368), and anti-rabbit Alexa 488-conjugated (Invitrogen). The BG4 antibody was obtained by transfection of the BG4 plasmid (kindly provided by S. Balasubramanian) in BL21 *Escherichia coli* cells. Then, BG4 protein expression was induced by the autoinduction method as described.^33^ BG4 was purified by using silica based resin (Protino Ni-IDA) precharged with Ni^2+^ ions and eluted with 250 mM imidazole/PBS pH 8.0. The eluted antibody was concentrated with Amicon Ultra-15 centrifugal filter units (Millipore), and imidazole was finally removed by buffer exchange with PBS pH 8.0 using Amicon Ultra-15 centrifugal filter units. The nuclei were stained with DAPI (Merck #D9542), and coverslips were mounted with Mowiol 4-88 (Sigma-Aldrich). The fluorescence signal was determined using ImageJ software. For the BG4 and TRF2 co-staining, cells have been fixed, permeabilized, and blocked as previously described,^17^ and co-incubated with 2 μg of BG4 and anti-TRF2 antibodies diluted to 1:500 (Abcam #13579) for 2 h at room temperature. Next, the cells have been incubated with the anti-FLAG antibody (Cell Signaling Technology #2368) for 1 h and then co-stained with the Alexa Fluor 488 anti-rabbit IgG (Life technologies #A11008) and the Alexa Fluor 594 anti-mouse IgG (Life technologies #A11032). For nuclear staining, cells were finally incubated with 2 μg/μL of DAPI for 20 min. The cover glasses were mounted with Mowiol 488.

#### S9.6 Immunofluorescence

Twenty-four hours after seeding, cells were treated as described in the figure caption, fixed with cold methanol at room temperature, and permeabilized with acetone on ice. After three washes with cold PBS, cells were blocked with 3% BSA, 0.1% Tween-20, and SSC 4X for 1 h. Then, the cells were incubated with a 5 μg/well S9.6 antibody. S9.6 has been purified from murine HB-8730 hybridoma cells as fully described elsewhere.^17^ Then, 1:1000 anti-Nucleolin (Abcam #ab22758) antibodies were diluted in 3% BSA, 0.1% Tween-20, and SSC 4X for 1 h at RT. Cells were then incubated at RT with 1:1000 Alexa Fluor 594 anti-mouse IgG (Life technologies #A11032) and 1:1000 Alexa Fluor 488 anti-rabbit IgG (Life technologies #A11008) in 3% BSA, 0.1% Tween-20, and SSC 4X for 1 h. After each step the cells were washed three times for 5 min with SSC 4X. For nuclear staining, cells were incubated with 2 μg/μL DAPI for 20 min, and the cover glasses were mounted with Mowiol 488.

#### 53BP1 and γH2AX Co-staining Immunofluorescence

Cells were treated with 10 μM Braco-19, compound **1**, or compound **15**. After 4 h of treatment, the cells were pre-extracted for 3 min at room temperature with a CSK buffer (10 mM PIPES pH 6.8, 100 mM NaCl, 300 mM sucrose, 3 mM MgCl_2_, 0.5% Triton X-100) and a Halt Protease Inhibitor cocktail (ThermoFisher #87785) and fixed in 2% paraformaldehyde for 15 min. Cells were stained with 1:500 anti-53BP1 (Cell signaling #S1981) and 1:500 of the anti-γH2AX antibody (Millipore #05-636) diluted in 5% BSA for 1 h at RT, followed by the incubation with 1:1000 of the secondary antibodies Alexa Fluor 488 anti-rabbit IgG (Life technologies #A11008) and Alexa Fluor 488 anti-mouse IgG (Life technologies #A11011). After each antibody incubation, cells were washed three times with PBS for 5 min. For nuclear staining, the cells were incubated with 2 μg/μL DAPI for 20 min, and the cover glasses were mounted with Mowiol 488. The immunofluorescence acquisition has been performed by a fluorescence microscope (Eclipse TE 2000-S; Nikon, Tokyo, Japan) equipped with an AxioCam MRm (Zeiss, Oberkochen, Germany) digital camera. Quantification of the fluorescence signal and foci has been performed by using the ImageJ software. For the DNA:RNA hybrid signal, we quantify the nucleoplasmic signal by subtracting the nucleolar signal, as visualized with nucleolin staining, from total nuclear fluorescence. Single cell fluorescence, or the number of foci per cell, has been normalized to the mean of the respective control sample (the untreated cells).

## Supporting Information Available

^1^H and ^13^C NMR spectra of compounds **1**–**15**, table of ligand-induced thermal stabilization of DNA molecules, CD and fluorescence spectra of DNA molecules, and quantification of levels of BG4 foci and of γH2AX (PDF)
Molecular formula strings (CSV)

## Abbreviations Used

G4: G-quadruplex
CBR: condensed benzene ring
NOE: nuclear Overhauser effect
DMF: dimethylformamide
CD: circular dichroism
FID: fluorescence intercalator displacement
TO: thiazole orange
FRET: Förster resonance energy transfer
FAM: fluorescein
TAMRA: carboxytetramethylrhodamine
IF: immunofluorescence
DSB: double-strand break
NHEJ: nonhomologous end joining
DMEM: Dulbecco’s Modified Eagle Medium
FBS: fetal bovine serum
DAPI: 4′,6-diamidino-2-phenylindole
PBS: phosphate buffered saline
BSA: bovine serum albumin
PIPES: 1,4-piperazinediethanesulfonic acid

## References

[ref1] Grand C. L.; Powell T. J.; Nagle R. B.; Bearss D. J.; Tye D.; Gleason-Guzman M.; Hurley L. H. Mutations in the G-Quadruplex Silencer Element and Their Relationship to c-MYC Overexpression, NM23 Repression, and Therapeutic Rescue. Proc. Natl. Acad. Sci. U. S. A. 2004, 101 (16), 6140–6145. 10.1073/pnas.0400460101. 15079086 PMC395936

[ref2] Siddiqui-Jain A.; Grand C. L.; Bearss D. J.; Hurley L. H. Direct Evidence for a G-Quadruplex in a Promoter Region and Its Targeting with a Small Molecule to Repress c-MYC Transcription. Proc. Natl. Acad. Sci. U. S. A. 2002, 99 (18), 11593–11598. 10.1073/pnas.182256799. 12195017 PMC129314

[ref3] Hänsel-Hertsch R.; Di Antonio M.; Balasubramanian S. DNA G-Quadruplexes in the Human Genome: Detection, Functions and Therapeutic Potential. Nat. Rev. Mol. Cell Biol. 2017, 18 (5), 279–284. 10.1038/nrm.2017.3. 28225080

[ref4] Li Y.; Syed J.; Suzuki Y.; Asamitsu S.; Shioda N.; Wada T.; Sugiyama H. Effect of ATRX and G-Quadruplex Formation by the VNTR Sequence on α-Globin Gene Expression. ChemBioChem 2016, 17 (10), 928–935. 10.1002/cbic.201500655. 26991472

[ref5] Whitehouse I.; Owen-Hughes T. ATRX: Put Me on Repeat. Cell 2010, 143 (3), 335–336. 10.1016/j.cell.2010.10.021. 21029854

[ref6] Valton A.-L.; Hassan-Zadeh V.; Lema I.; Boggetto N.; Alberti P.; Saintome C.; Riou J.-F.; Prioleau M.-N. G4Motifs Affect Origin Positioning and Efficiency in Two Vertebrate Replicators. EMBO J. 2014, 33 (7), 732–746. 10.1002/embj.201387506. 24521668 PMC4000090

[ref7] Rodriguez R.; Miller K. M.; Forment J. V.; Bradshaw C. R.; Nikan M.; Britton S.; Oelschlaegel T.; Xhemalce B.; Balasubramanian S.; Jackson S. P. Small-Molecule–Induced DNA Damage Identifies Alternative DNA Structures in Human Genes. Nat. Chem. Biol. 2012, 8 (3), 301–310. 10.1038/nchembio.780. 22306580 PMC3433707

[ref8] Paeschke K.; Bochman M. L.; Garcia P. D.; Cejka P.; Friedman K. L.; Kowalczykowski S. C.; Zakian V. A. Pif1 Family Helicases Suppress Genome Instability at G-Quadruplex Motifs. Nature 2013, 497 (7450), 458–462. 10.1038/nature12149. 23657261 PMC3680789

[ref9] Paeschke K.; Capra J. A.; Zakian V. A. DNA Replication through G-Quadruplex Motifs Is Promoted by the Saccharomyces Cerevisiae Pif1 DNA Helicase. Cell 2011, 145 (5), 678–691. 10.1016/j.cell.2011.04.015. 21620135 PMC3129610

[ref10] Lopes J.; Piazza A.; Bermejo R.; Kriegsman B.; Colosio A.; Teulade-Fichou M.-P.; Foiani M.; Nicolas A. G-Quadruplex-Induced Instability during Leading-Strand Replication. EMBO J. 2011, 30 (19), 4033–4046. 10.1038/emboj.2011.316. 21873979 PMC3209785

[ref11] Sarkies P.; Reams C.; Simpson L. J.; Sale J. E. Epigenetic Instability Due to Defective Replication of Structured DNA. Mol. Cell 2010, 40 (5), 703–713. 10.1016/j.molcel.2010.11.009. 21145480 PMC3145961

[ref12] Schiavone D.; Jozwiakowski S. K.; Romanello M.; Guilbaud G.; Guilliam T. A.; Bailey L. J.; Sale J. E.; Doherty A. J. PrimPol Is Required for Replicative Tolerance of G Quadruplexes in Vertebrate Cells. Mol. Cell 2016, 61 (1), 161–169. 10.1016/j.molcel.2015.10.038. 26626482 PMC4712188

[ref13] Spiegel J.; Adhikari S.; Balasubramanian S. The Structure and Function of DNA G-Quadruplexes. Trends Chem. 2019, 1–14. 10.1016/j.trechm.2019.07.002. PMC7472594 32923997

[ref14] Pelliccia S.; Amato J.; Capasso D.; Di Gaetano S.; Massarotti A.; Piccolo M.; Irace C.; Tron G. C.; Pagano B.; Randazzo A.; Novellino E.; Giustiniano M. Bio-Inspired Dual-Selective BCL-2/c-MYC G-Quadruplex Binders: Design, Synthesis, and Anticancer Activity of Drug-like Imidazo[2,1-i]purine Derivatives. J. Med. Chem. 2019, 10.1021/acs.jmedchem.9b00262. 31241946

[ref15] Cimino-Reale G.; Zaffaroni N.; Folini M. Emerging Role of G-Quadruplex DNA as Target in Anticancer Therapy. Curr. Pharm. Des. 2017, 22 (44), 6612–6624. 10.2174/1381612822666160831101031. 27587203

[ref16] van Wietmarschen N.; Merzouk S.; Halsema N.; Spierings D. C. J.; Guryev V.; Lansdorp P. M. BLM Helicase Suppresses Recombination at G-Quadruplex Motifs in Transcribed Genes. Nat. Commun. 2018, 9 (1), 271 10.1038/s41467-017-02760-1. 29348659 PMC5773480

[ref17] De Magis A.; Manzo S. G.; Russo M.; Marinello J.; Morigi R.; Sordet O.; Capranico G. DNA Damage and Genome Instability by G-Quadruplex Ligands Are Mediated by R Loops in Human Cancer Cells. Proc. Natl. Acad. Sci. U. S. A. 2019, 116 (3), 816–825. 10.1073/pnas.1810409116. 30591567 PMC6338839

[ref18] Chédin F. Nascent Connections: R-Loops and Chromatin Patterning. Trends Genet. 2016, 32 (12), 828–838. 10.1016/j.tig.2016.10.002. 27793359 PMC5123964

[ref19] Aguilera A.; Gómez-González B. DNA–RNA Hybrids: The Risks of DNA Breakage during Transcription. Nat. Struct. Mol. Biol. 2017, 24 (5), 439–443. 10.1038/nsmb.3395. 28471430

[ref20] Amato J.; Morigi R.; Pagano B.; Pagano A.; Ohnmacht S.; De Magis A.; Tiang Y. P.; Capranico G.; Locatelli A.; Graziadio A.; Leoni A.; Rambaldi M.; Novellino E.; Neidle S.; Randazzo A. Toward the Development of Specific G-Quadruplex Binders: Synthesis, Biophysical, and Biological Studies of New Hydrazone Derivatives. J. Med. Chem. 2016, 59 (12), 5706–5720. 10.1021/acs.jmedchem.6b00129. 27223049

[ref21] Amato J.; Iaccarino N.; Pagano B.; Morigi R.; Locatelli A.; Leoni A.; Rambaldi M.; Zizza P.; Biroccio A.; Novellino E.; Randazzo A. Bis-Indole Derivatives with Antitumor Activity Turn out to Be Specific Ligands of Human Telomeric G-Quadruplex. Front. Chem. 2014, 2, 54 10.3389/fchem.2014.00054. 25105115 PMC4109613

[ref22] Andreani A.; Burnelli S.; Granaiola M.; Leoni A.; Locatelli A.; Morigi R.; Rambaldi M.; Varoli L.; Landi L.; Prata C.; Sega F. V. D.; Caliceti C.; Shoemaker R. H. Antitumor Activity and COMPARE Analysis of Bis-Indole Derivatives. Bioorg. Med. Chem. 2010, 18 (9), 3004–3011. 10.1016/j.bmc.2010.03.063. 20395150

[ref23] Pagano B.; Cosconati S.; Gabelica V.; Petraccone L.; De Tito S.; Marinelli L.; La Pietra V.; Saverio di Leva F.; Lauri I.; Trotta R.; Novellino E.; Giancola C.; Randazzo A. State-of-the-Art Methodologies for the Discovery and Characterization of DNA G-Quadruplex Binders. Curr. Pharm. Des. 2012, 18 (14), 1880–1899. 10.2174/138161212799958332. 22376104

[ref24] Dai J.; Carver M.; Yang D. Polymorphism of Human Telomeric Quadruplex Structures. Biochimie 2008, 90 (8), 1172–1183. 10.1016/j.biochi.2008.02.026. 18373984 PMC2556180

[ref25] Xue Y.; Kan Z.; Wang Q.; Yao Y.; Liu J.; Hao Y.; Tan Z. Human Telomeric DNA Forms Parallel-Stranded Intramolecular G-Quadruplex in K + Solution under Molecular Crowding Condition. J. Am. Chem. Soc. 2007, 129 (36), 11185–11191. 10.1021/ja0730462. 17705383

[ref26] Masiero S.; Trotta R.; Pieraccini S.; De Tito S.; Perone R.; Randazzo A.; Spada G. P. A Non-Empirical Chromophoric Interpretation of CD Spectra of DNA G-Quadruplex Structures. Org. Biomol. Chem. 2010, 8 (12), 2683–2692. 10.1039/c003428b. 20440429

[ref27] Karsisiotis A. I.; Hessari N. M. A.; Novellino E.; Spada G. P.; Randazzo A.; Webba Da Silva M. Topological Characterization of Nucleic Acid G-Quadruplexes by UV Absorption and Circular Dichroism. Angew. Chem., Int. Ed. 2011, 50 (45), 10645–10648. 10.1002/anie.201105193. 21928459

[ref28] Luu K. N.; Phan A. T.; Kuryavyi V.; Lacroix L.; Patel D. J. Structure of the Human Telomere in K+ Solution: An Intramolecular (3 + 1) G-Quadruplex Scaffold. J. Am. Chem. Soc. 2006, 128 (30), 9963–9970. 10.1021/ja062791w. 16866556 PMC4692383

[ref29] Monchaud D.; Allain C.; Teulade-Fichou M.-P. Development of a Fluorescent Intercalator Displacement Assay (G4-FID) for Establishing Quadruplex-DNA Affinity and Selectivity of Putative Ligands. Bioorg. Med. Chem. Lett. 2006, 16 (18), 4842–4845. 10.1016/j.bmcl.2006.06.067. 16837195

[ref30] Largy E.; Hamon F.; Teulade-Fichou M.-P. Development of a High-Throughput G4-FID Assay for Screening and Evaluation of Small Molecules Binding Quadruplex Nucleic Acid Structures. Anal. Bioanal. Chem. 2011, 400 (10), 3419–3427. 10.1007/s00216-011-5018-z. 21528379

[ref31] De Cian A.; Guittat L.; Kaiser M.; Saccà B.; Amrane S.; Bourdoncle A.; Alberti P.; Teulade-Fichou M. P.; Lacroix L.; Mergny J. L. Fluorescence-Based Melting Assays for Studying Quadruplex Ligands. Methods 2007, 42 (2), 183–195. 10.1016/j.ymeth.2006.10.004 10.1016/j.ymeth.2006.10.004. 17472900

[ref32] Pagano A.; Iaccarino N.; Abdelhamid M. A. S.; Brancaccio D.; Garzarella E. U.; Di Porzio A.; Novellino E.; Waller Z. A. E.; Pagano B.; Amato J.; Randazzo A. Common G-Quadruplex Binding Agents Found to Interact With i-Motif-Forming DNA: Unexpected Multi-Target-Directed Compounds. Front. Chem. 2018, 6, 281 10.3389/fchem.2018.00281. 30137743 PMC6066642

[ref33] Biffi G.; Tannahill D.; McCafferty J.; Balasubramanian S. Quantitative Visualization of DNA G-Quadruplex Structures in Human Cells. Nat. Chem. 2013, 5 (3), 182–186. 10.1038/nchem.1548. 23422559 PMC3622242

[ref34] Gowan S. M.; Harrison J. R.; Patterson L.; Valenti M.; Read M. a; Neidle S.; Kelland L. R. A G-Quadruplex-Interactive Potent Small-Molecule Inhibitor of Telomerase Exhibiting in Vitro and in Vivo Antitumor Activity. Mol. Pharmacol. 2002, 61 (5), 1154–1162. 10.1124/mol.61.5.1154. 11961134

[ref35] Burger A. M.; Dai F.; Schultes C. M.; Reszka A. P.; Moore M. J.; Double J. A.; Neidle S. The G-Quadruplex-Interactive Molecule BRACO-19 Inhibits Tumor Growth, Consistent with Telomere Targeting and Interference with Telomerase Function. Cancer Res. 2005, 65 (4), 1489–1496. 10.1158/0008-5472.CAN-04-2910. 15735037

[ref36] Duquette M. L. Intracellular Transcription of G-Rich DNAs Induces Formation of G-Loops, Novel Structures Containing G4 DNA. Genes Dev. 2004, 18 (13), 1618–1629. 10.1101/gad.1200804. 15231739 PMC443523

[ref37] Santos-Pereira J. M.; Aguilera A. R Loops: New Modulators of Genome Dynamics and Function. Nat. Rev. Genet. 2015, 16 (10), 583–597. 10.1038/nrg3961. 26370899

[ref38] Kuo L. J.; Yang L. X. γ-H2AX- A Novel Biomarker for DNA Double-Strand Breaks. In Vivo (Brooklyn). 2008, 22 (3), 305–310. 18610740

[ref39] Chapman J. R.; Sossick A. J.; Boulton S. J.; Jackson S. P. BRCA1-Associated Exclusion of 53BP1 from DNA Damage Sites Underlies Temporal Control of DNA Repair. J. Cell Sci. 2012, 125 (15), 3529–3534. 10.1242/jcs.105353. 22553214 PMC3445322

[ref40] Hatch E. M.; Fischer A. H.; Deerinck T. J.; Hetzer M. W. Catastrophic Nuclear Envelope Collapse in Cancer Cell Micronuclei. Cell 2013, 154 (1), 47–60. 10.1016/j.cell.2013.06.007. 23827674 PMC3749778

[ref41] Sparapani S.; Bellini S.; Gunaratnam M.; Haider S. M.; Andreani A.; Rambaldi M.; Locatelli A.; Morigi R.; Granaiola M.; Varoli L.; Burnelli S.; Leoni A.; Neidle S. Bis-Guanylhydrazone Diimidazo[1,2-a:1,2-c]Pyrimidine as a Novel and Specific G-Quadruplex Binding Motif. Chem. Commun. 2010, 46 (31), 5680–5682. 10.1039/c0cc00020e. 20582382

[ref42] Andreani A.; Granaiola M.; Leoni A.; Locatelli A.; Morigi R.; Rambaldi M.; Giorgi G.; Salvini L. Cancer Fighting Cancer: Synthesis of the New Heterocyclic Systemdiimidazo-[1,2-a:1,2-c]-Pyrimidine. Arkivoc 2002, (xi), 32–38. 10.3998/ark.5550190.0003.b04.

[ref43] Andreani A.; Rambaldi M.; Bonazzi D.; Greci L.; Andreani F. Study on Compounds with Potential Antitumor Activity. III. Hydrazonic Derivatives of 5-Substituted 2-Chloro-3-Formyl-6-Methylindole. *Farm. Ed*. Sci. 1979, 10 (24), 132–138. 10.1002/chin.197924211. 553830

[ref44] Andreani A.; Burnelli S.; Granaiola M.; Leoni A.; Locatelli A.; Morigi R.; Rambaldi M.; Varoli L.; Calonghi N.; Cappadone C.; Farruggia G.; Zini M.; Stefanelli C.; Masotti L. Substituted E-3-(2-Chloro-3-Indolylmethylene)1,3-Dihydroindol-2-Ones with Antitumor Activity. Effect on the Cell Cycle and Apoptosis. J. Med. Chem. 2007, 50 (14), 3167–3172. 10.1021/jm070235m. 17559205

[ref45] Lu S. C.; Zhang X. X.; Shi Z. J.; Ren Y. W.; Li B.; Zhang W. Intramolecular Photochemical Cross-Coupling Reactions of 3-Acyl-2-Haloindoles and 2-Chloropyrrole-3-Carbaldehydes with Substituted Benzenes. Adv. Synth. Catal. 2009, 351 (17), 2839–2844. 10.1002/adsc.200900540.

[ref46] Allen G. R.; Binovi L. J.; Weiss M. J. The Mitomycin Antibiotics. Synthetic Studies. XVI.1 the Utilization of 5-Methoxy-4-Nitro-3-Indolecarboxaldehydes for the Synthesis of Related 4, 7-Indoloquinones2. J. Med. Chem. 1967, 10 (1), 7–13. 10.1021/jm00313a002. 6031708

[ref47] Ramya P. V. S.; Guntuku L.; Angapelly S.; Digwal C. S.; Lakshmi U. J.; Sigalapalli D. K.; Babu B. N.; Naidu V. G. M.; Kamal A. Synthesis and Biological Evaluation of Curcumin Inspired Imidazo[1,2-a]Pyridine Analogues as Tubulin Polymerization Inhibitors. Eur. J. Med. Chem. 2018, 143, 216–231. 10.1016/j.ejmech.2017.11.010. 29174816

[ref48] Stanley L. M.; Hartwig J. F. Iridium-Catalyzed Regio- and Enantioselective N-Allylation of Indoles. Angew. Chem., Int. Ed. 2009, 48 (42), 7841–7844. 10.1002/anie.200904338. PMC2819323 19760689

[ref49] Mayer F.; Oppenheimer T. Über Naphthyl-Essigsäuren. 3. Abhandlung: 1-Nitronaphthyl-2-Brenztraubensäure Und 1-Nitronaphthyl-2-Essigsäure. Ber. Dtsch. Chem. Ges. 1918, 51 (2), 1239–1245. 10.1002/cber.19180510210.

[ref50] Andreani A.; Granaiola M.; Leoni A.; Locatelli A.; Morigi R.; Rambaldi M.; Garaliene V. Synthesis and Antitumor Activity of 1,5,6-Substituted E −3-(2-Chloro-3-Indolylmethylene)-1,3-Dihydroindol-2-Ones 1. J. Med. Chem. 2002, 45 (12), 2666–2669. 10.1021/jm011123c. 12036377

[ref51] Pagano B.; Amato J.; Iaccarino N.; Cingolani C.; Zizza P.; Biroccio A.; Novellino E.; Randazzo A. Looking for Efficient G-Quadruplex Ligands: Evidence for Selective Stabilizing Properties and Telomere Damage by Drug-like Molecules. ChemMedChem 2015, 10 (4), 640–649. 10.1002/cmdc.201402552. 25694275

[ref52] Cantor C. R.; Warshaw M. M.; Shapiro H. Oligonucleotide Interactions. III. Circular Dichroism Studies of the Conformation of Deoxyoligonucleolides. Biopolymers 1970, 9 (9), 1059–1077. 10.1002/bip.1970.360090909. 5449435

[ref53] Giancola C.; Pagano B. Energetics of ligand binding to G- quadruplexes. Top. Curr. Chem. 2012, 330, 211–242. 10.1007/128_2012_347. 22851158

