# Supplementary material for: Monohydrazone Based G-Quadruplex Selective Ligands Induce DNA Damage and Genome Instability in Human Cancer Cells
Source: J Med Chem. 2020 Mar 6;63(6):3090–103. doi: 10.1021/acs.jmedchem.9b01866 (PMC7997572; doi:10.1021/acs.jmedchem.9b01866)
Supplement: Supplementary file 1 — jm9b01866_si_001.pdf [file jm9b01866_si_001.pdf]

Supporting Information

Mono-hydrazone based G-quadruplex selective ligands induce DNA damage and genome instability in human cancer cells

Jussara Amato,[†,#] Giulia Miglietta,[‡,#] Rita Morigi,*[,‡,#] Nunzia Iaccarino,[†] Alessandra Locatelli,[‡] Alberto Leoni,[‡] Ettore Novellino,[†] Bruno Pagano,[†] Giovanni Capranico,*[,‡,$] and Antonio Randazzo*[,†,$]

[†]*Department of Pharmacy, University of Naples Federico II, via D. Montesano 49, 80131 Naples, Italy*

[‡]*Department of Pharmacy and Biotechnology, Alma Mater Studiorum - University of Bologna, 40126 Bologna, Italy*

[#]These authors contributed equally.

[$]Co-last Authors.

*Corresponding Authors.

R.M.: phone: +39 051 2099712; e-mail: rita.morigi@unibo.it.

G.C.: phone: +39 051 2091209; e-mail: giovanni.capranico@unibo.it.

A.R.: phone: +39 081 678514; e-mail: antonio.randazzo@unina.it.

Table of contents

No.	Content	Page
1	^{1}H and ^{13}C NMR spectra of the final compounds **1-15**.	S3 - S17
2	**Table S1**. Ligand-induced thermal stabilization (ΔT_m) of G4 and duplex DNA molecules measured by CD melting experiments.	S18
3	**Figure S1**. Circular dichroism spectra of the investigated G4s (15 μM) in the absence and presence of 10 mol. equiv. of compounds **1-15**.	S19
4	**Figure S2**. Circular dichroism spectra of the hairpin-duplex DNA (*Hairpin*) (30 μM) in the absence and presence of 10 mol. equiv. of compounds **1-15**.	S20
5	**Figure S3**. Circular dichroism spectrum of *F-Tel$_{21}$-T* (1 μM) in 20 mM potassium phosphate buffer (pH 7.0) containing 5 mM KCl, prepared by dilution from the highly concentrated (10 mM) DNA solution in annealing.	S20
6	**Figure S4**. Fluorescence spectra of *F-Tel21-T-p* (left panel) and *F-ckit2-T* (right panel) alone at 5 °C (blue line) and 100 °C (dashed blue line), and in the presence of 10 mol. equiv. of compound **15** at 5° C (green line).	S20
7	**Figure S5**. G4 stabilization induced by ligands at 24 h of treatment in U2OS cancer cells. (**A**) Raw data of BG4 fluorescence quantification after 24 h of treatment with compounds **1** (10 μM) and **15** (2 μM) in two biological replicates. (**B**) Raw data of BG4 foci quantification after 24 h of treatment with compounds **1** (10 μM), **15** (2 μM) and Braco-19 (10 μM) in U2OS cells in two biological replicates.	S21
8	**Figure S6**. G4 stabilization by 24 h of treatment with compound **15**. Representative images of BG4 foci in untreated cells (top panel) and in cell after 24 h of treatment with **15** (bottom panel). Scale bar is 10 μm.	S22
9	**Figure S7**. BG4 foci levels quantification in human U2OS cells induced by compounds **1** and **15** after short time of treatments. BG4 foci quantification with **1** (10 μM) and **15** (10 μM) after 5, 30 and 60 min of treatment. Values are means +/- SEM of two biological replicates. Significance has been evaluated by Kologorov-Smirnov parametric test: * $p<0.05$; ** $p>0.01$; *** $p>0.001$; **** $p<0.0001$ with GraphPad software. Numbers above box plot indicate cells nuclei analyzed. Graphs shows three biological replicates.	S22
10	**Figure S8**. γH2AX fluorescence induced after 4 h of treatment with compounds **1** and **15** (10 μM) in U2OS cancer cells. Each of the 3 graphs shows a biological replicate.	S23

^{1}H and ^{13}C NMR spectra of the final compounds 1-15.

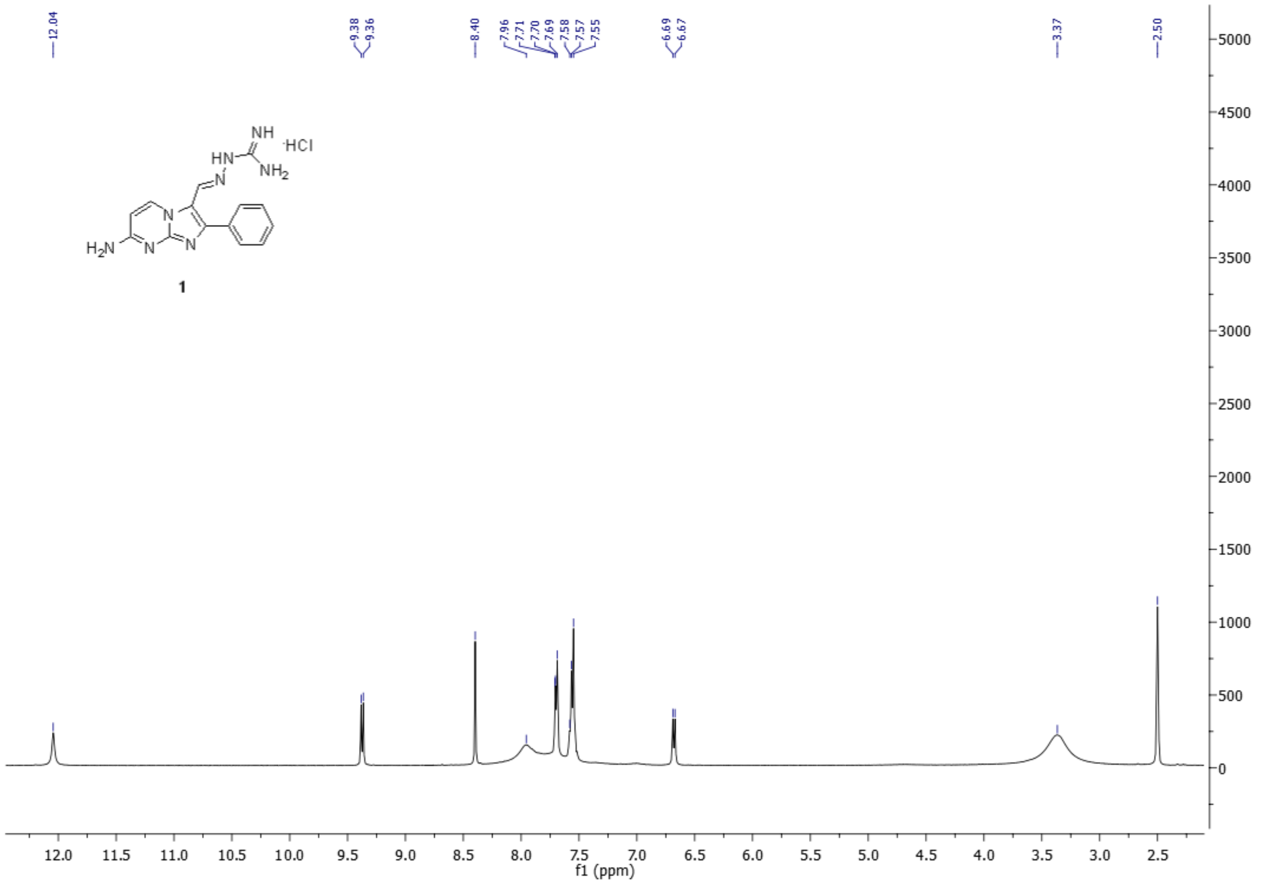

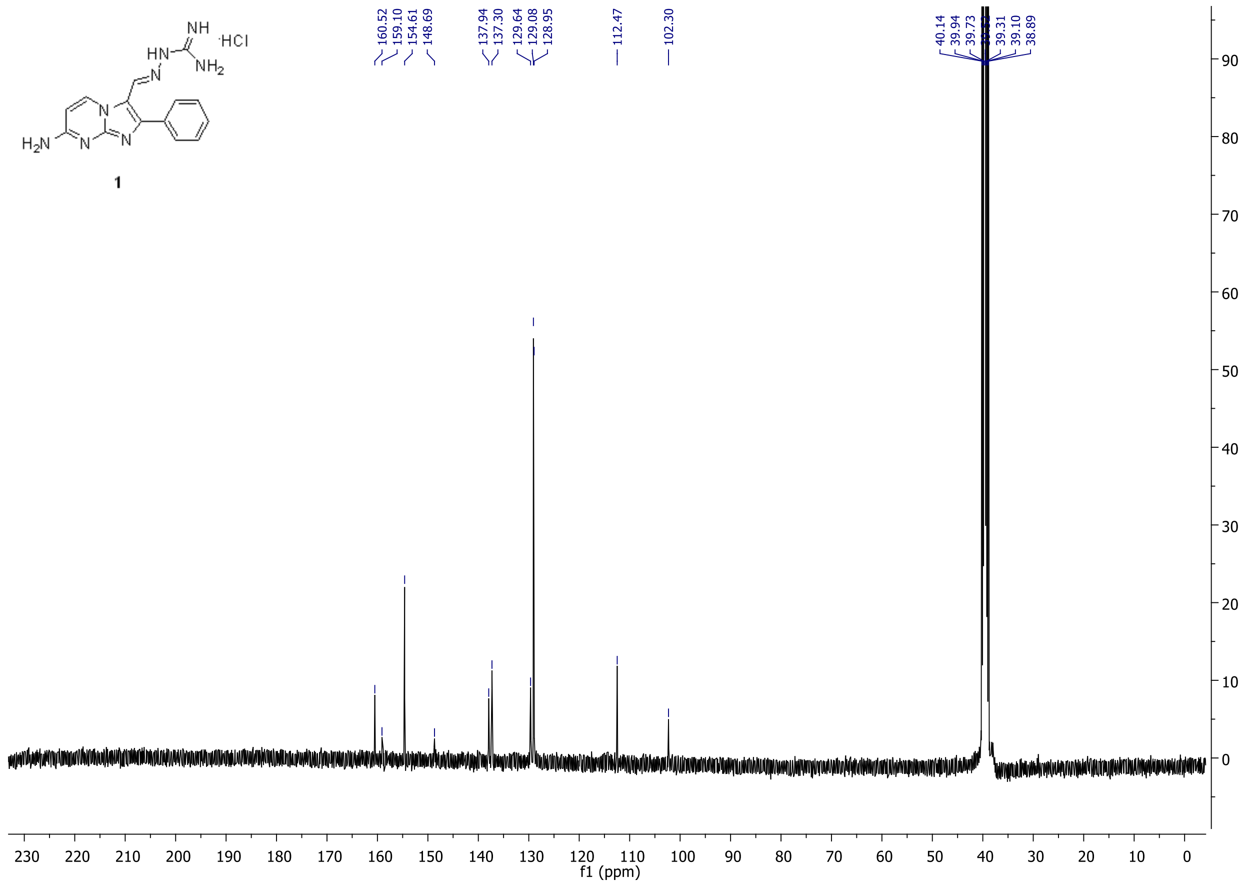

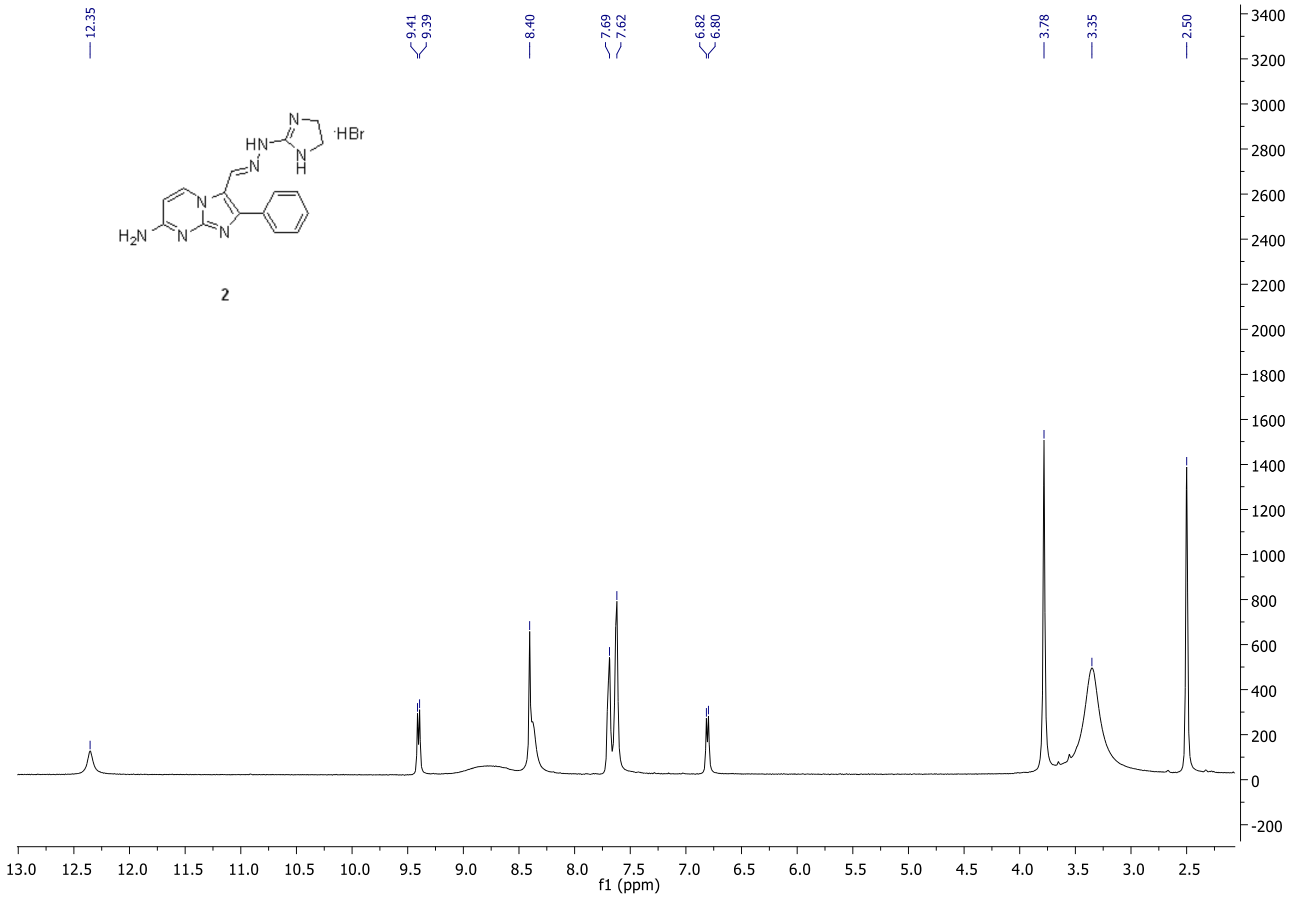
12.35
9.41
9.39
8.40
7.69
7.62
6.82
6.80
3.78
3.35
2.50
HN
N
H
·HBr
H2N
2
f1 (ppm)

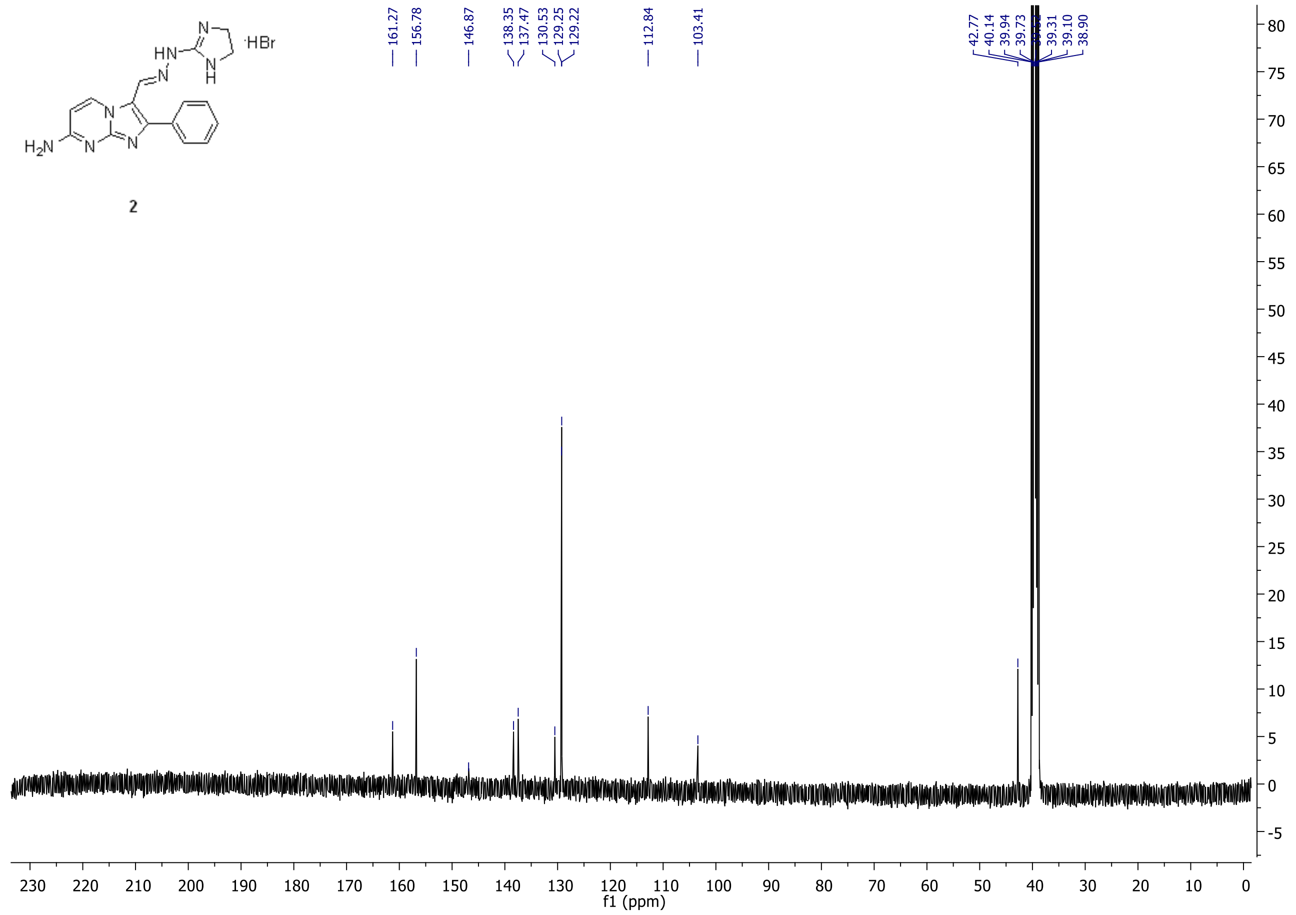
161.27
156.78
146.87
138.35
137.47
130.53
129.25
129.22
112.84
103.41
42.77
40.14
39.94
39.73
39.31
39.10
38.90
·HBr
H2N
2
f1 (ppm)

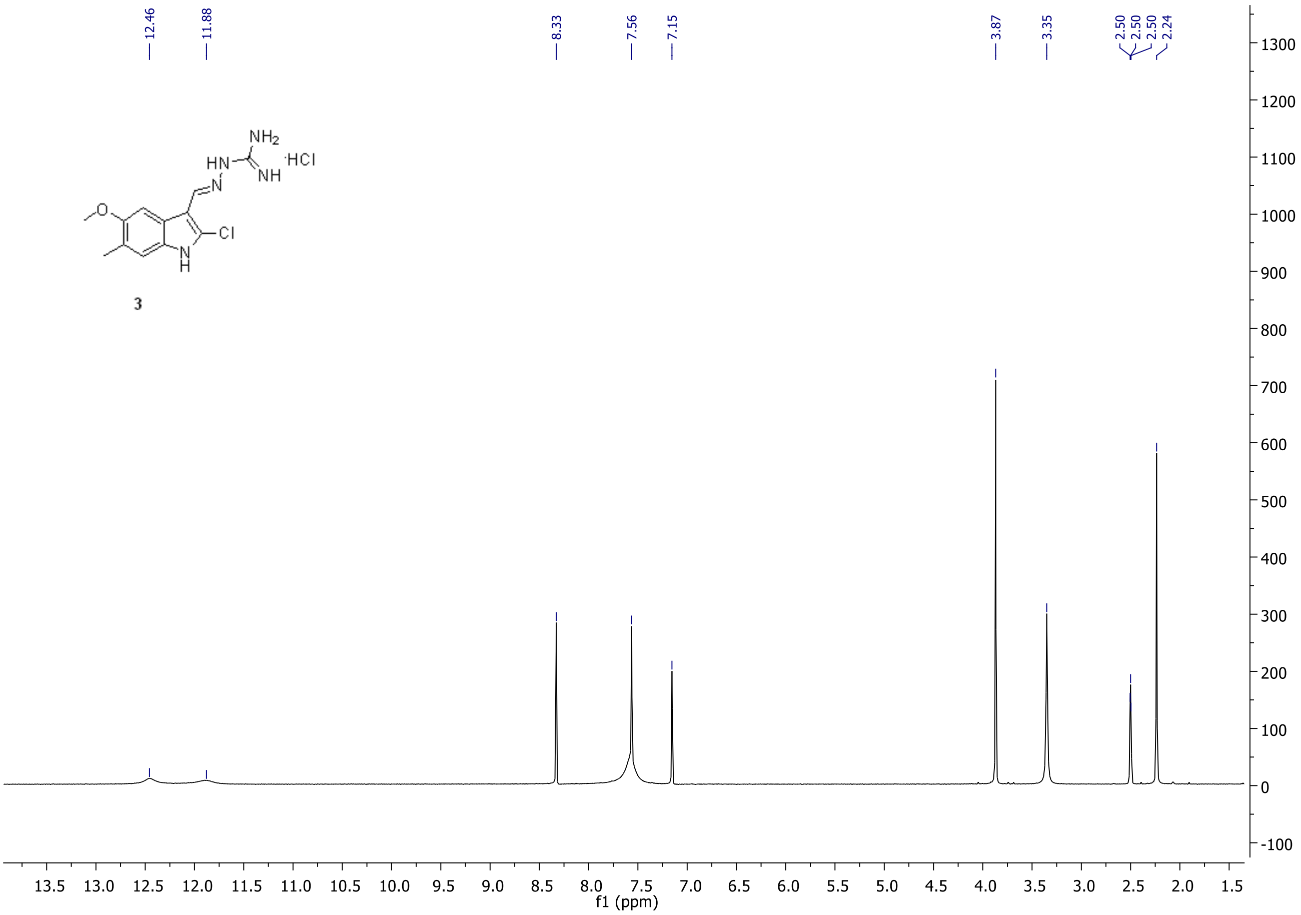
12.46
11.88
8.33
7.56
7.15
3.87
3.35
2.50
2.50
2.50
2.24
NH2
HN
·HCl
NH
N
O
Cl
N
H
3
1300
1200
1100
1000
900
800
700
600
500
400
300
200
100
0
-100
13.5 13.0 12.5 12.0 11.5 11.0 10.5 10.0 9.5 9.0 8.5 8.0 7.5 7.0 6.5 6.0 5.5 5.0 4.5 4.0 3.5 3.0 2.5 2.0 1.5
f1 (ppm)

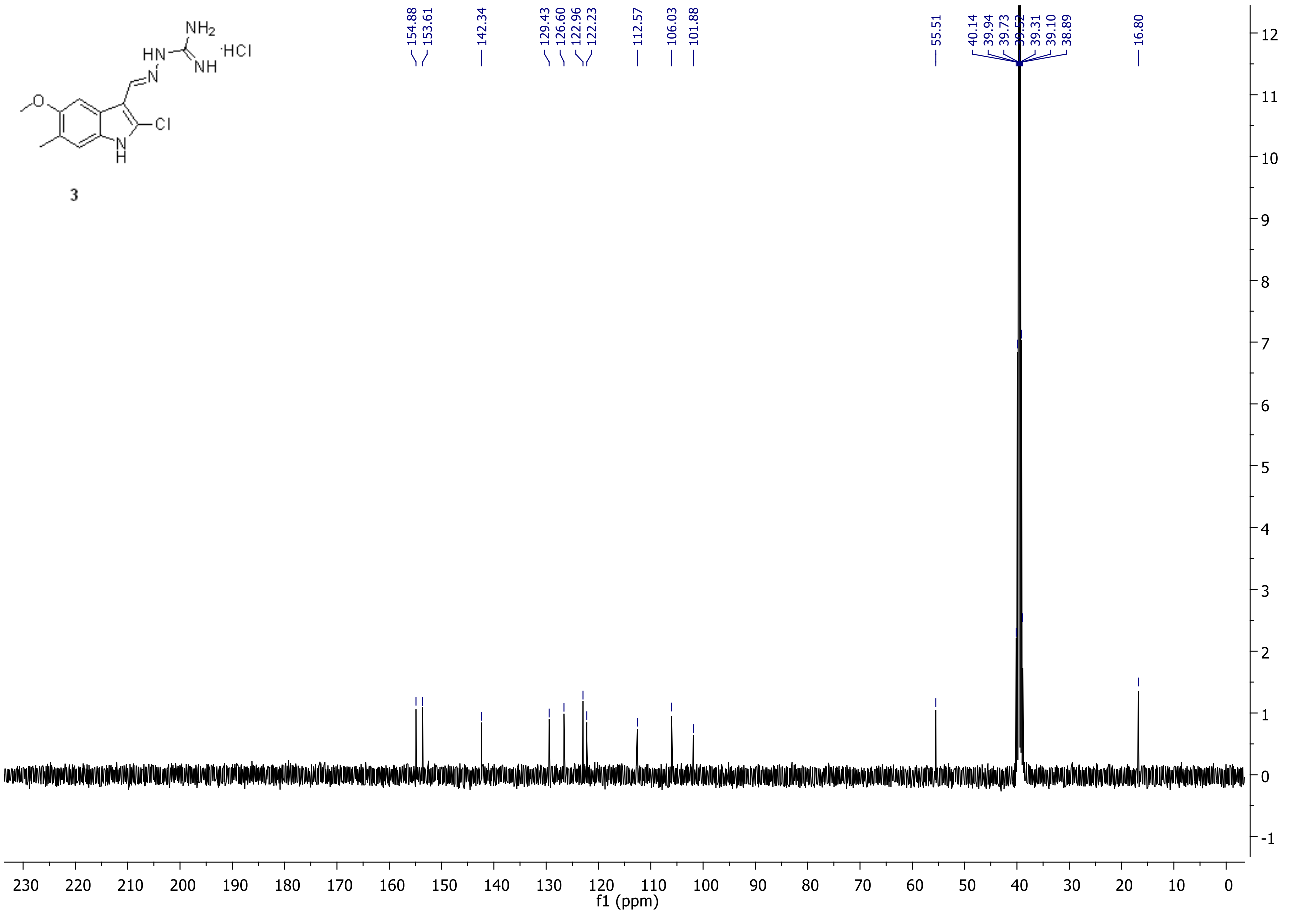
154.88
153.61
142.34
129.43
126.60
122.96
122.23
112.57
106.03
101.88
55.51
40.14
39.94
39.73
39.52
39.31
39.10
38.89
16.80
NH2
HN
·HCl
NH
N
O
Cl
N
H
3
12
11
10
9
8
7
6
5
4
3
2
1
0
-1
230 220 210 200 190 180 170 160 150 140 130 120 110 100 90 80 70 60 50 40 30 20 10 0
f1 (ppm)

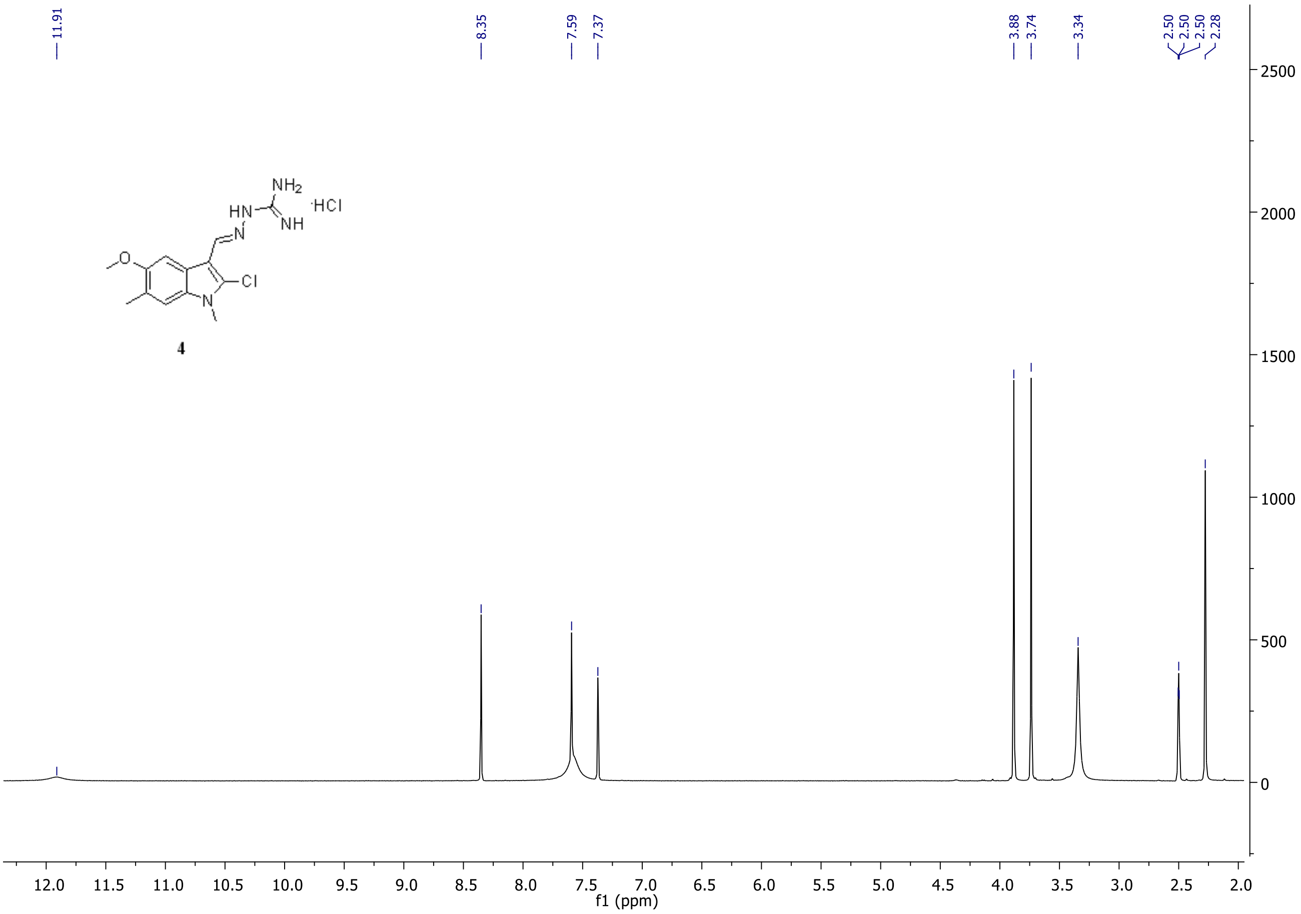
11.91
8.35
7.59
7.37
3.88
3.74
3.34
2.50
2.50
2.50
2.28
NH2
HN
N
NH
·HCl
O
Cl
N
4
f1 (ppm)

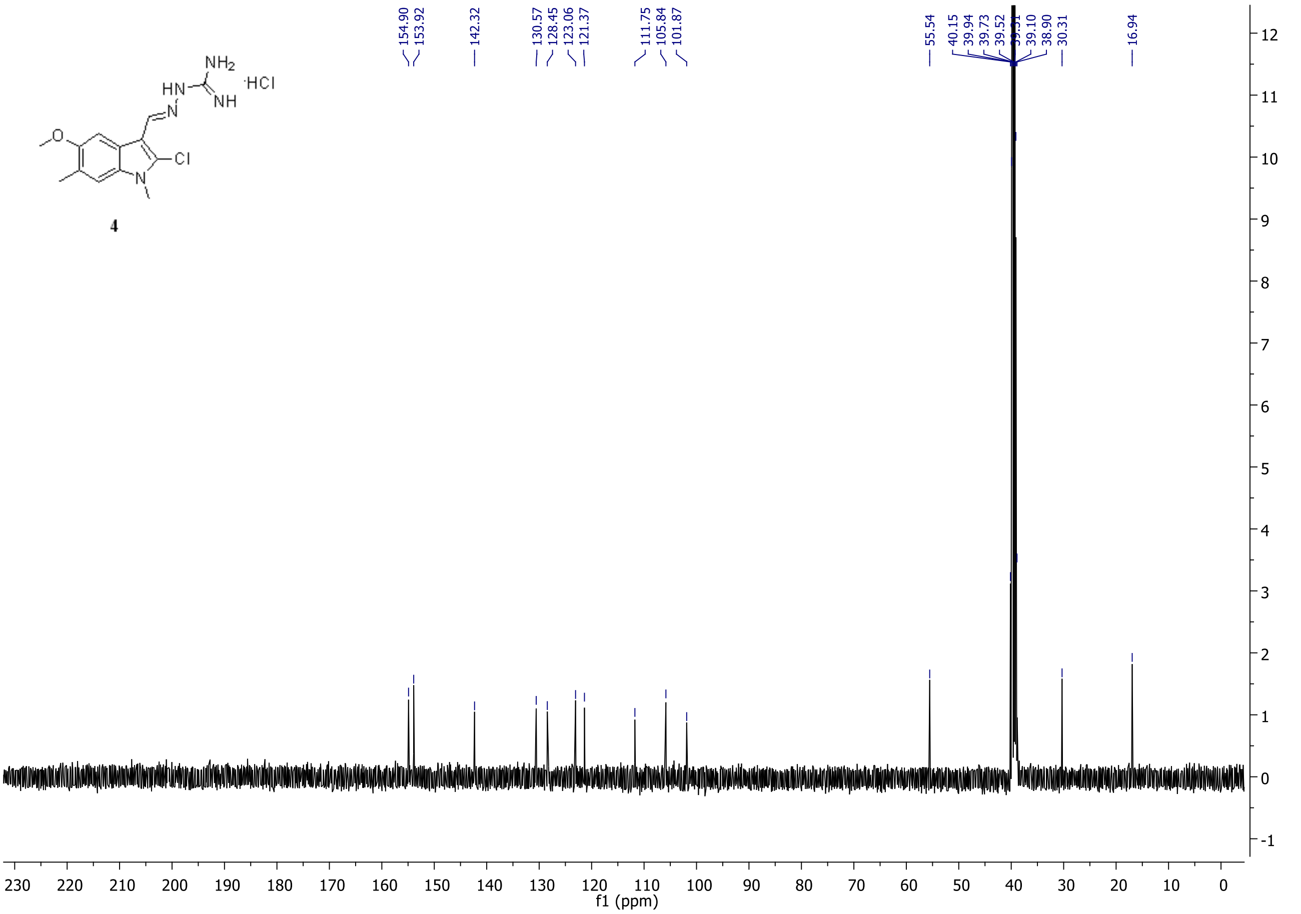
154.90
153.92
142.32
130.57
128.45
123.06
121.37
111.75
105.84
101.87
55.54
40.15
39.94
39.73
39.52
39.10
38.90
30.31
16.94
NH2
HN
N
NH
·HCl
O
Cl
N
4
f1 (ppm)

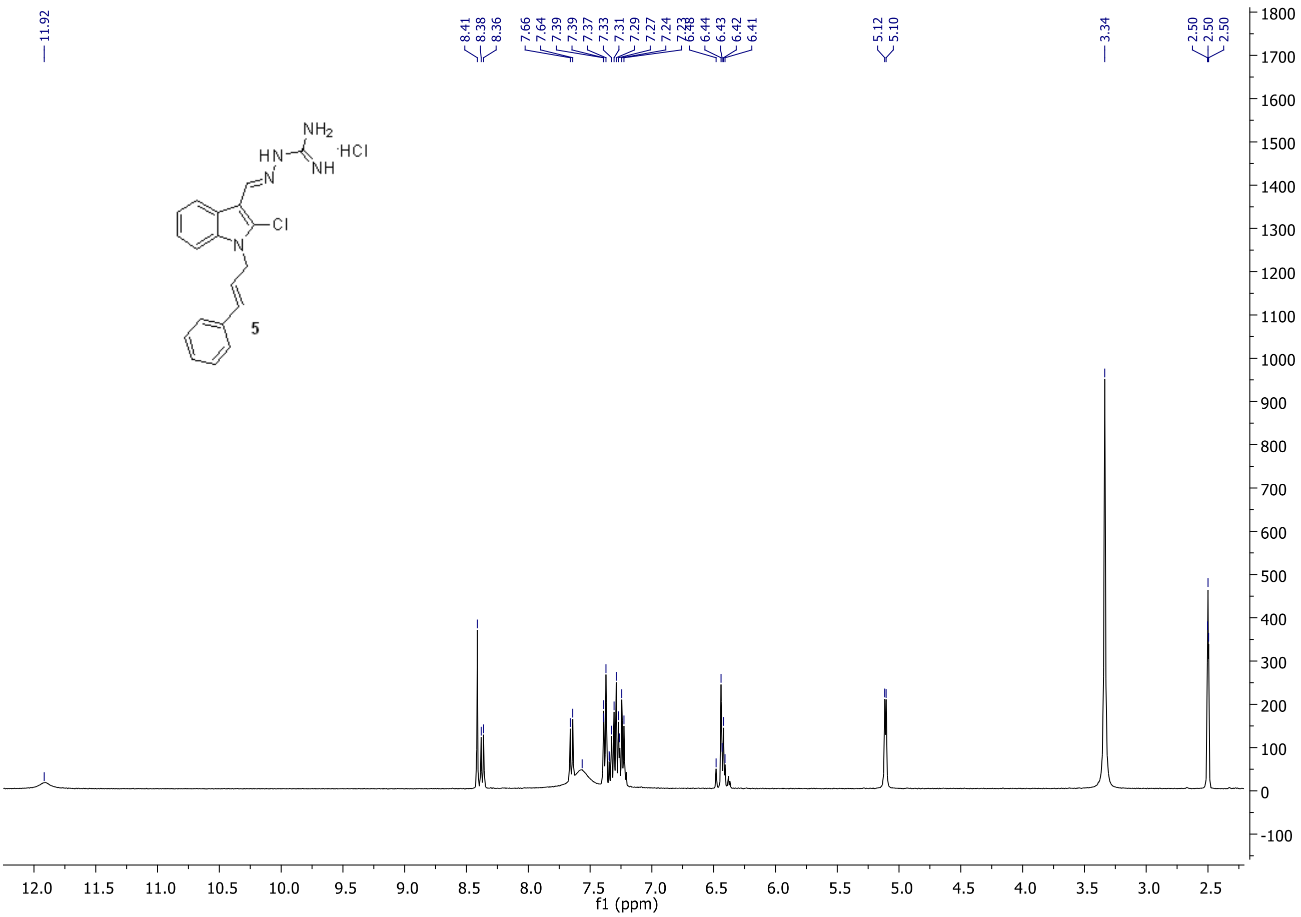

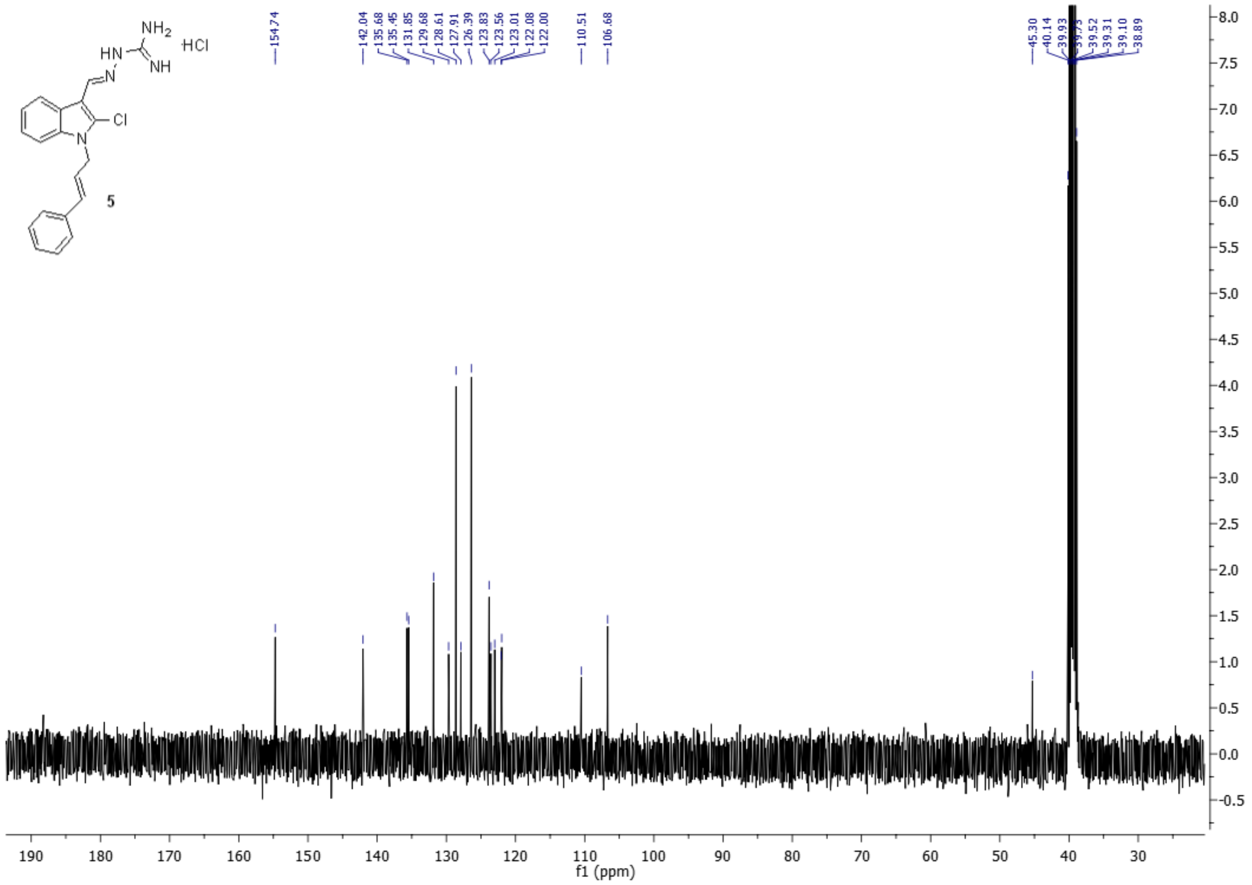

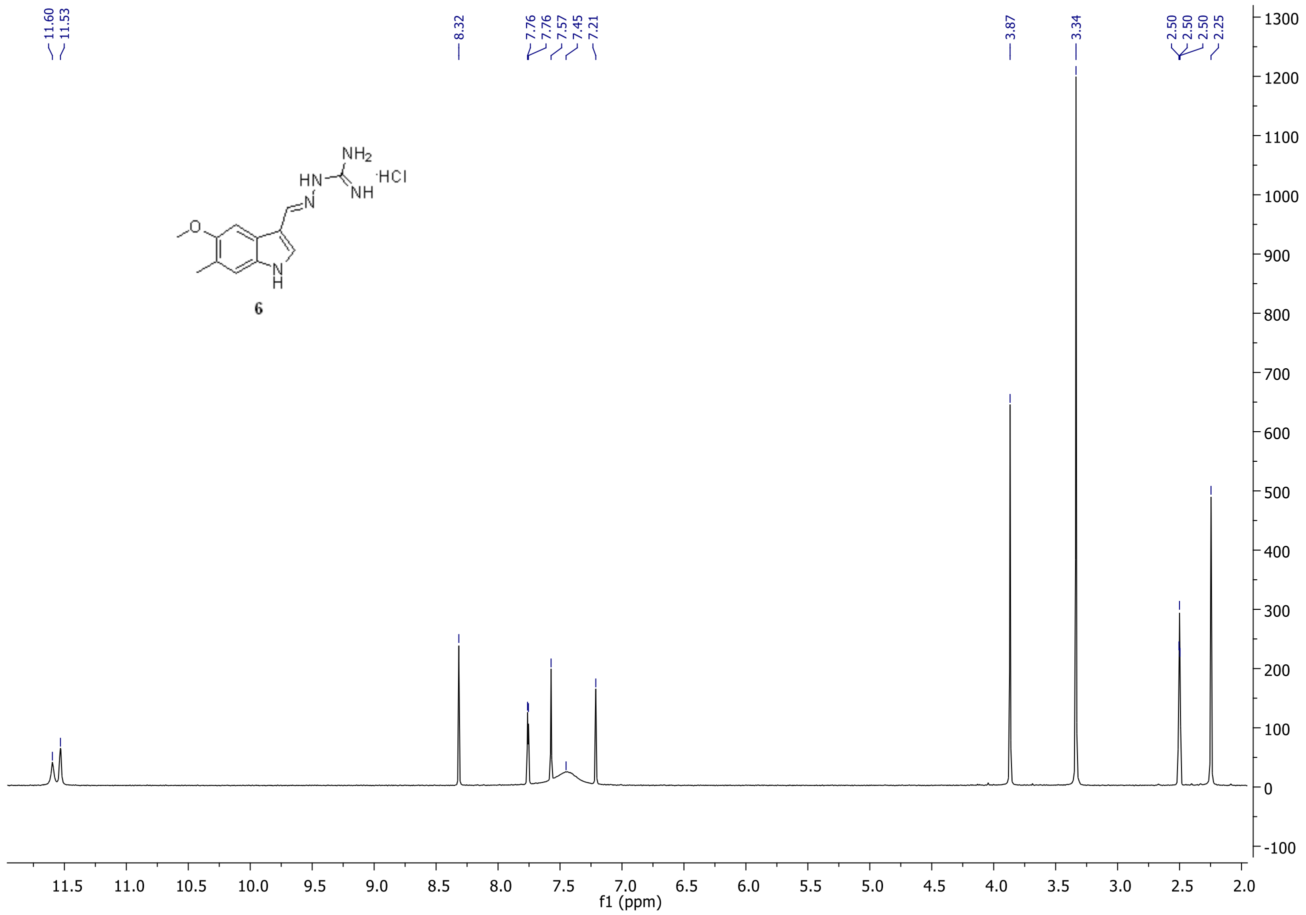
11.60
11.53
8.32
7.76
7.76
7.57
7.45
7.21
3.87
3.34
2.50
2.50
2.50
2.25
NH2
HN
N
NH
·HCl
O
N
H
6
f1 (ppm)

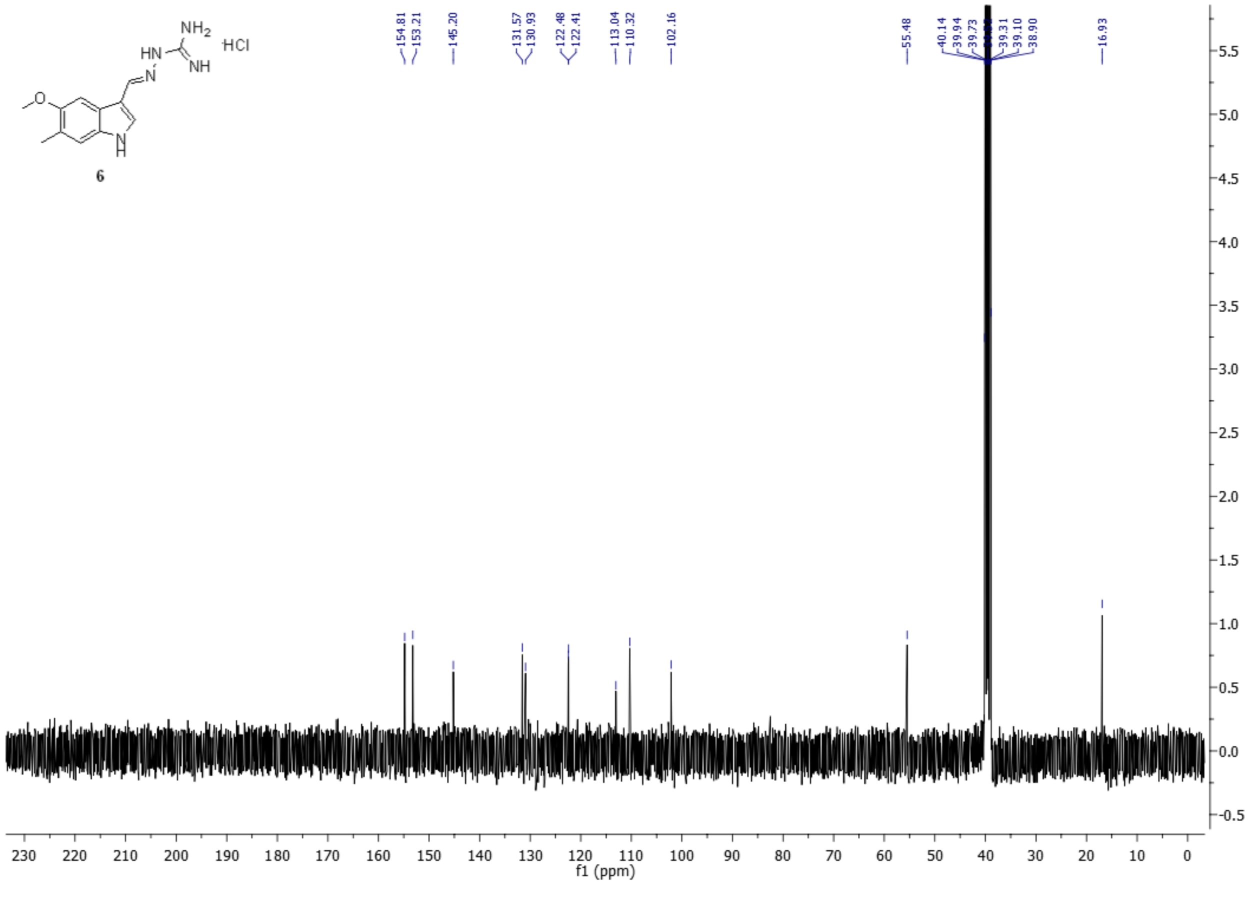
154.81
153.21
145.20
131.57
130.93
122.48
122.41
113.04
110.32
102.16
55.48
40.14
39.94
39.73
39.31
39.10
38.90
16.93
NH2
HN
N
NH
·HCl
O
N
H
6
f1 (ppm)

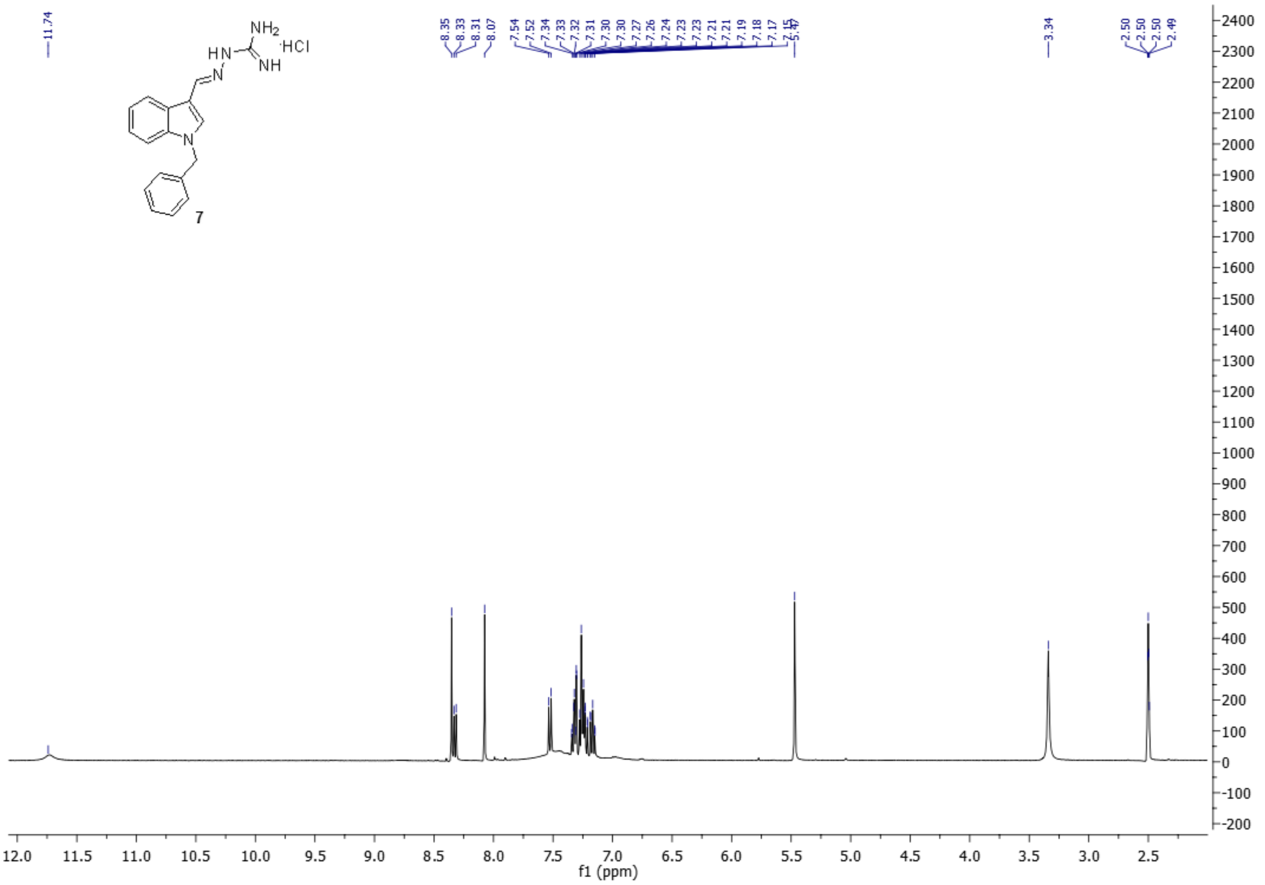
NH2
HN
HCl
NH
N
7
11.74
8.35 8.33 8.31 8.07 7.54 7.52 7.34 7.33 7.32 7.31 7.30 7.30 7.27 7.26 7.24 7.23 7.23 7.21 7.21 7.19 7.18 7.17 7.15 5.47
3.34
2.50 2.50 2.50 2.49
f1 (ppm)

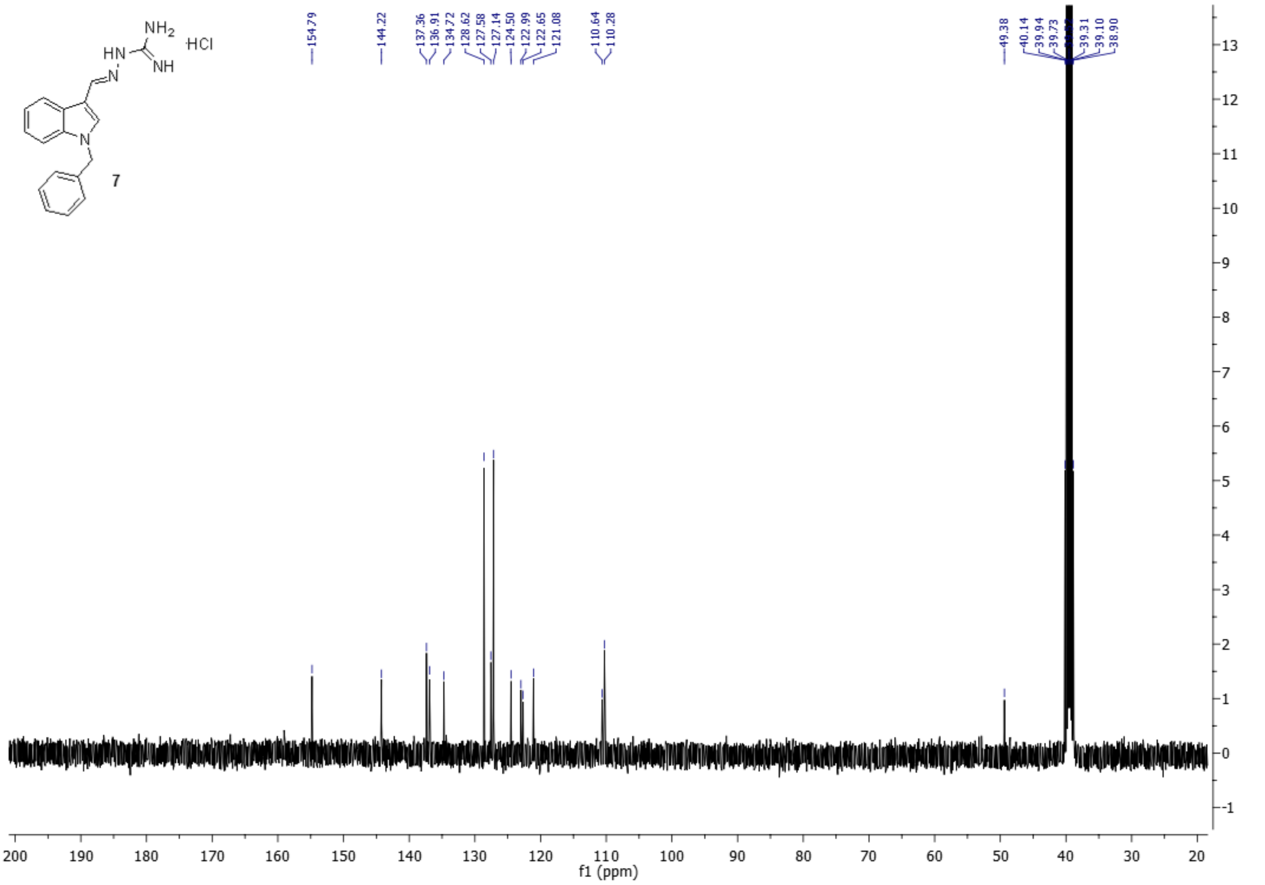
NH2
HN
HCl
NH
N
7
154.79
144.22
137.36 136.91 134.72 128.62 127.58 127.14 124.50 122.99 122.65 121.08
110.64 110.28
49.38
40.14 39.94 39.73 39.31 39.10 38.90
f1 (ppm)

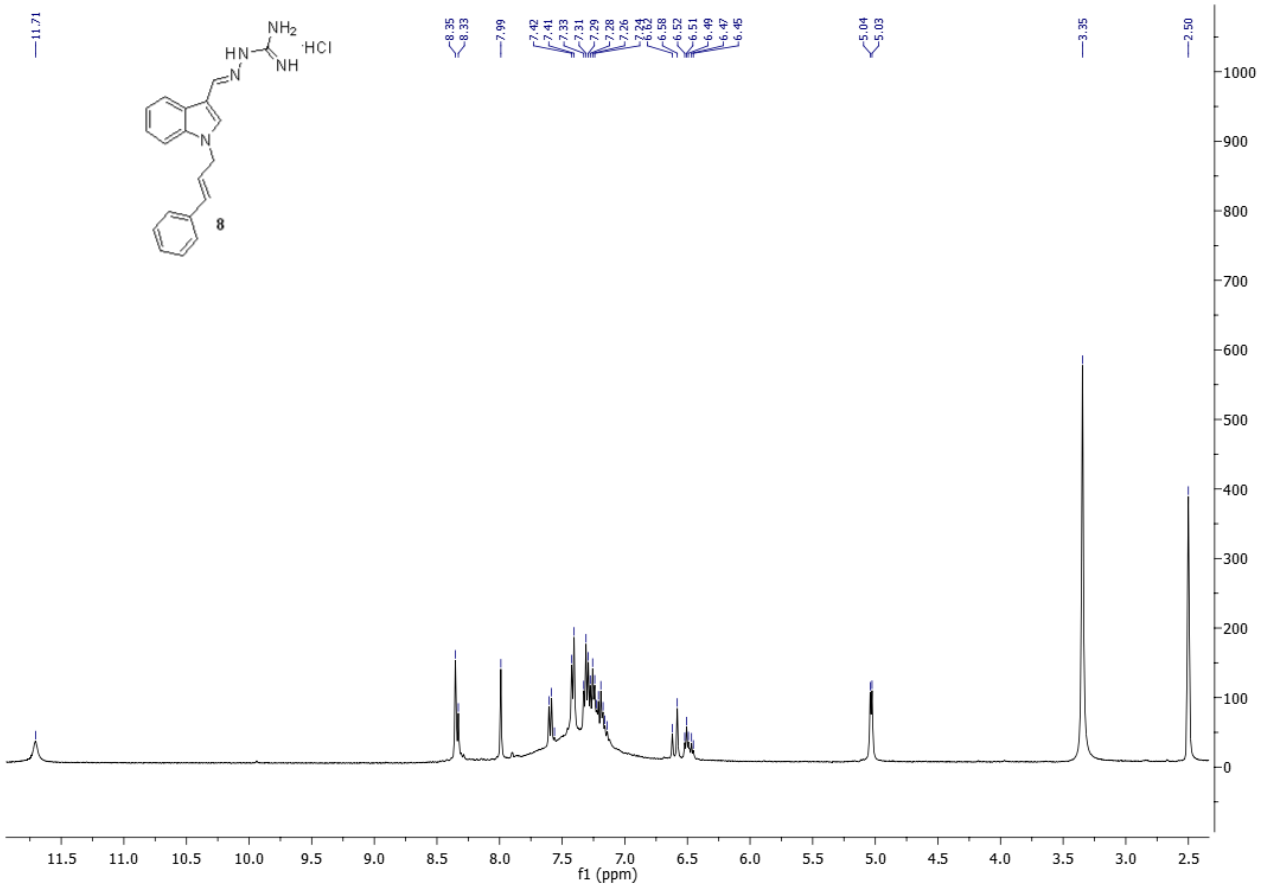
11.71
8.35 8.33 7.99 7.42 7.41 7.33 7.31 7.29 7.28 7.26 7.24 6.62 6.58 6.52 6.51 6.49 6.47 6.45
5.04 5.03
3.35
2.50
HCl
8
f1 (ppm)

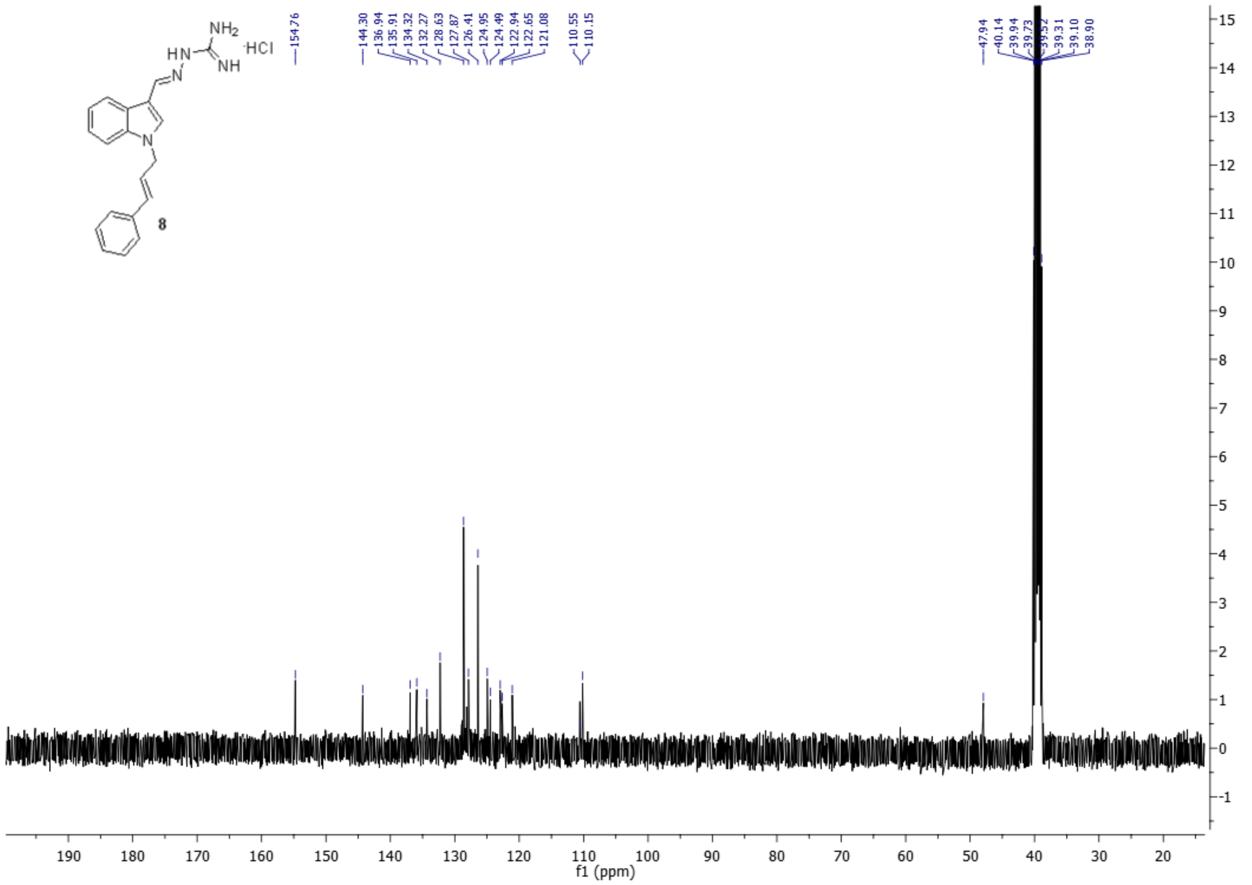
154.76
144.30 136.94 135.91 134.32 132.27 128.63 127.87 126.41 124.95 124.49 122.94 122.65 121.08
110.55 110.15
47.94 40.14 39.94 39.73 39.52 39.31 39.10 38.90
HCl
8
f1 (ppm)

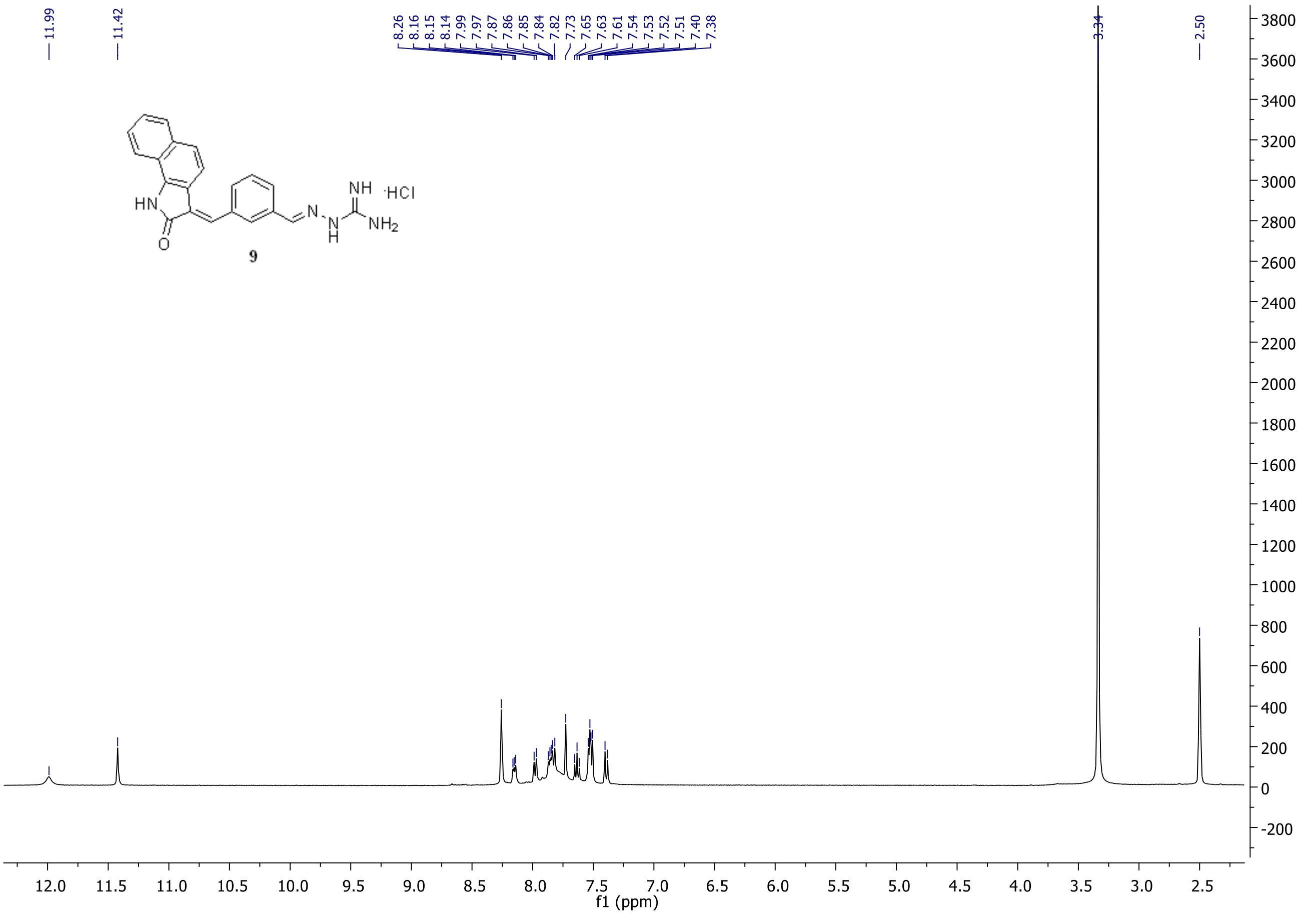
11.99
11.42
8.26 8.16 8.15 8.14 7.99 7.97 7.87 7.86 7.85 7.84 7.82 7.73 7.65 7.63 7.61 7.54 7.53 7.52 7.51 7.40 7.38
3.34
2.50
HN
O
NH
·HCl
N
N
H
NH2
9
f1 (ppm)

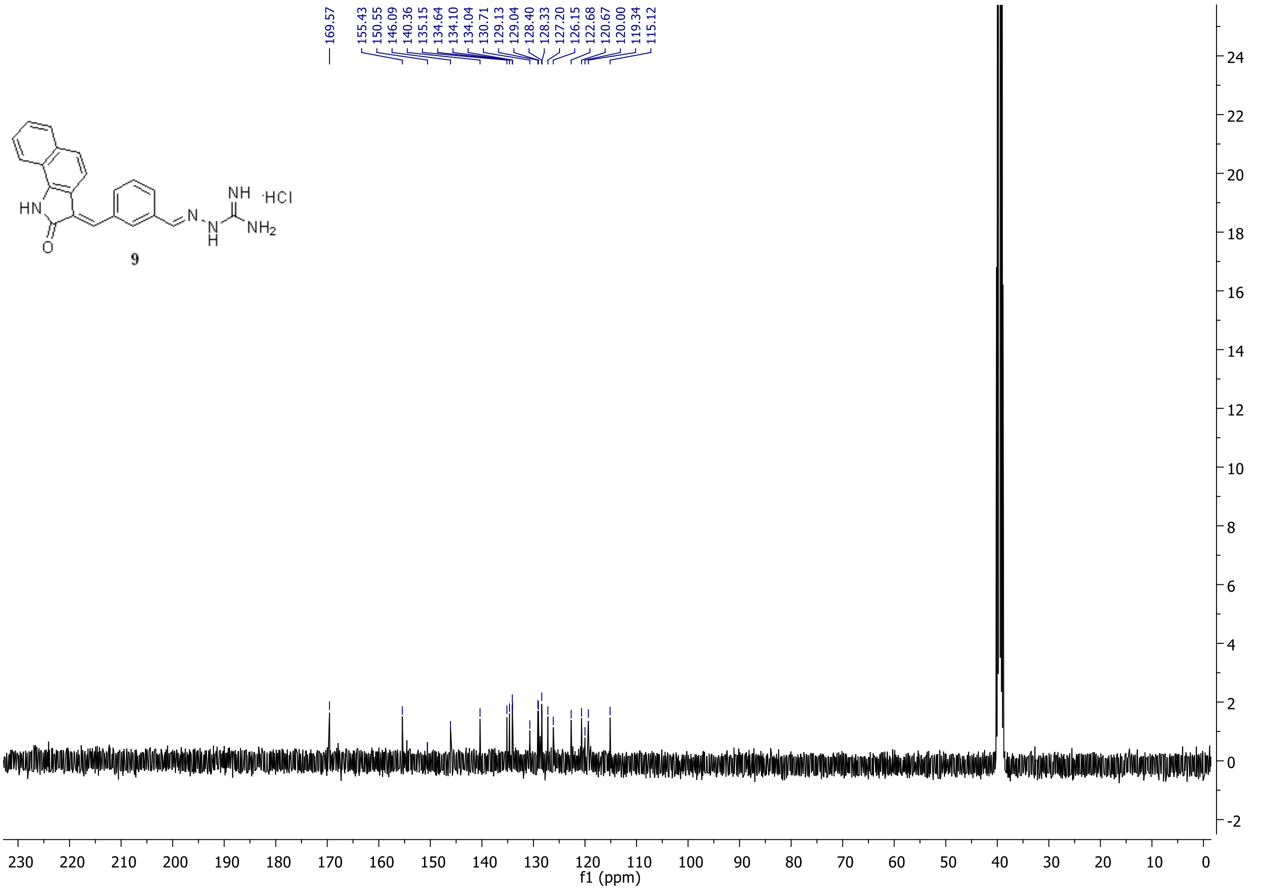
169.57
155.43 150.55 146.09 140.36 135.15 134.64 134.10 134.04 130.71 129.13 129.04 128.40 128.33 127.20 126.15 122.68 120.67 120.00 119.34 115.12
HN
O
NH
·HCl
N
N
H
NH2
9
f1 (ppm)

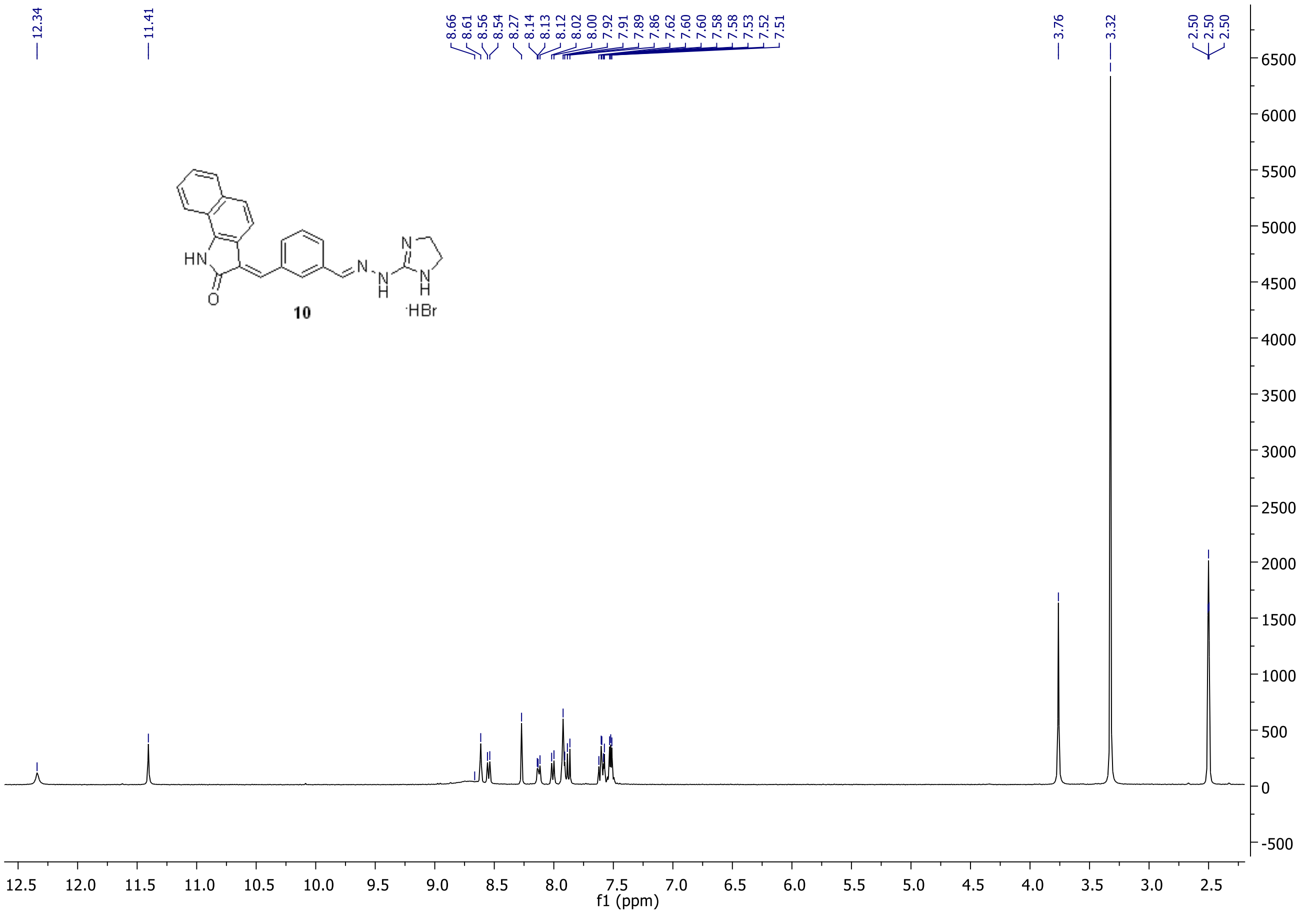
12.34
11.41
8.66 8.61 8.56 8.54 8.27 8.14 8.13 8.12 8.02 8.00 7.92 7.91 7.89 7.86 7.62 7.60 7.60 7.58 7.53 7.52 7.51
3.76
3.32
2.50 2.50 2.50
HN
O
N
N
H
N
H
10
·HBr
6500 6000 5500 5000 4500 4000 3500 3000 2500 2000 1500 1000 500 0 -500
12.5 12.0 11.5 11.0 10.5 10.0 9.5 9.0 8.5 8.0 7.5 7.0 6.5 6.0 5.5 5.0 4.5 4.0 3.5 3.0 2.5
f1 (ppm)

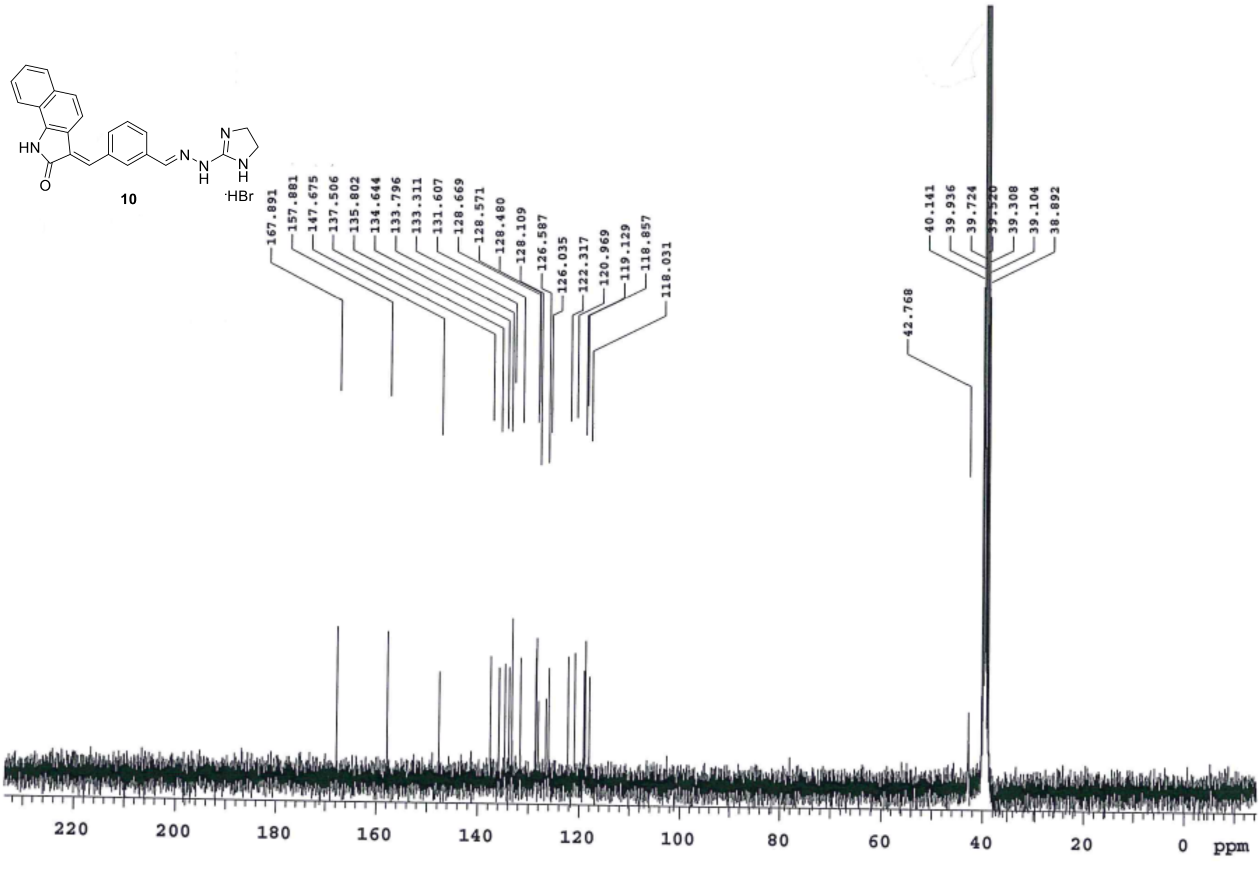
HN
O
N
N
H
N
H
10
·HBr
167.891 157.881 147.675 137.506 135.802 134.644 133.796 133.311 131.607 128.669 128.571 128.480 128.109 126.587 126.035 122.317 120.969 119.129 118.857 118.031
40.141 39.936 39.724 39.520 39.308 39.104 38.892
42.768
220 200 180 160 140 120 100 80 60 40 20 0 ppm

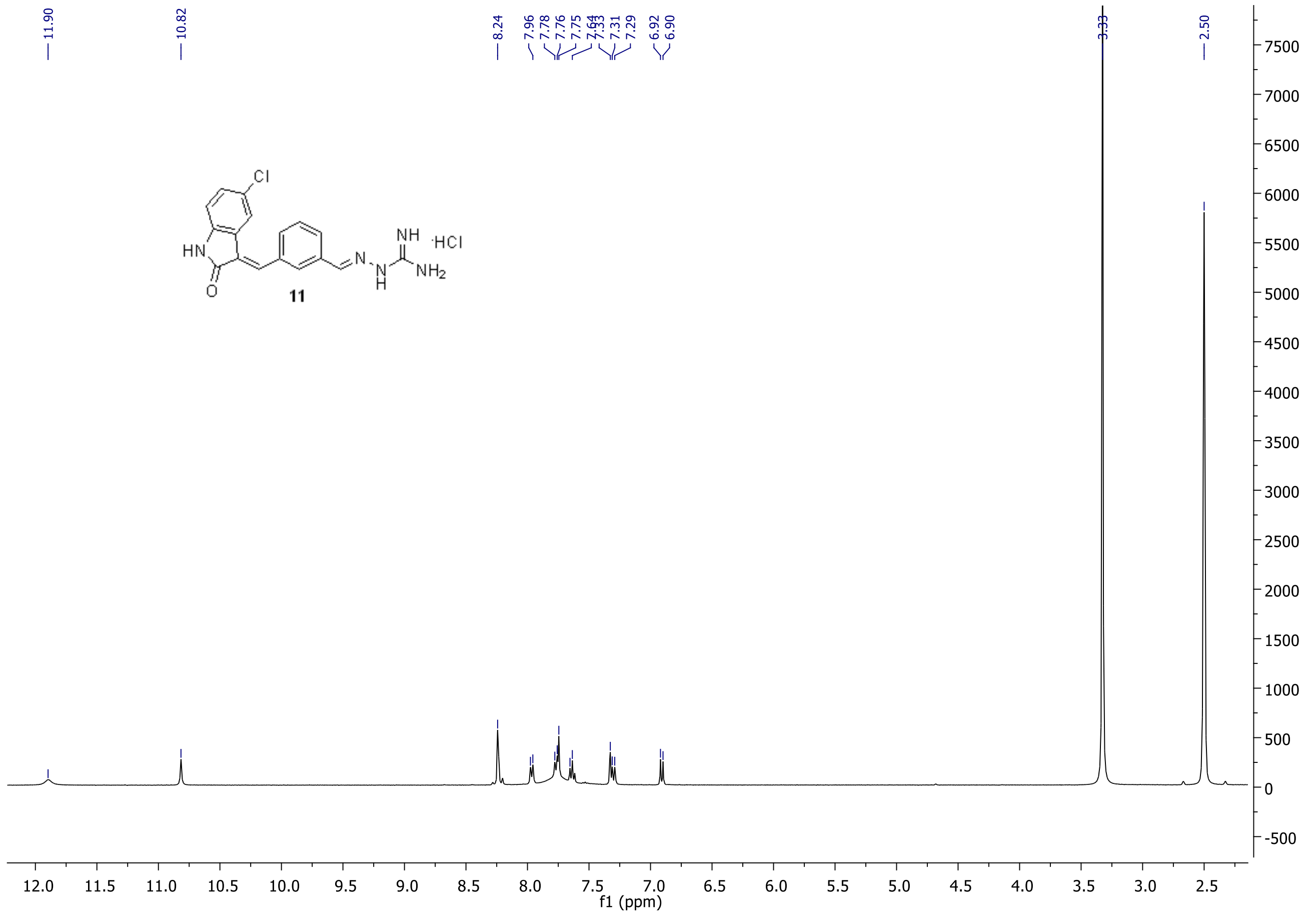
11.90
10.82
8.24
7.96
7.78
7.76
7.75
7.31
7.29
6.92
6.90
3.33
2.50
Cl
HN
O
NH
·HCl
N
N
H
NH2
11
f1 (ppm)

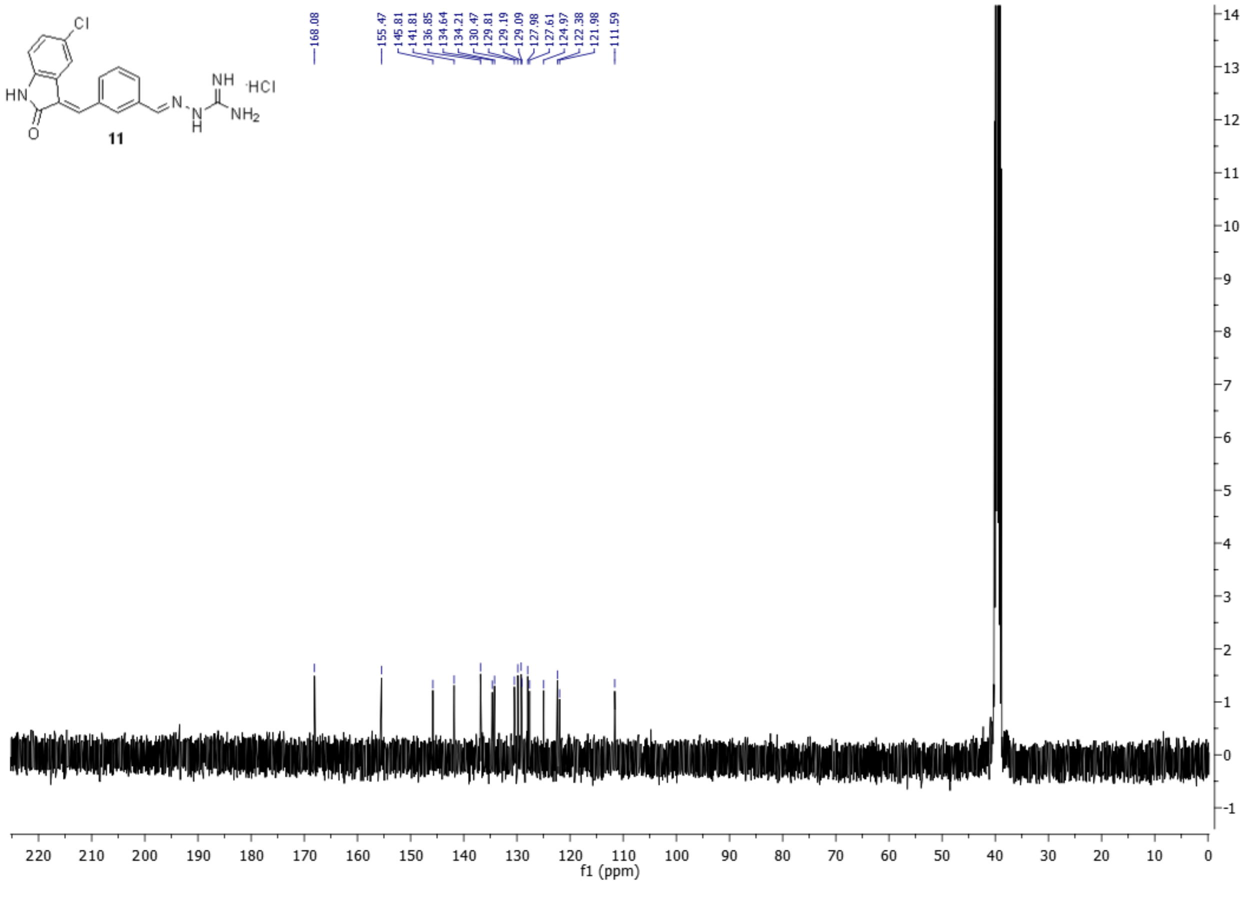
168.08
155.47
145.81
141.81
136.85
134.64
134.21
130.47
129.81
129.19
129.09
127.98
127.61
124.97
122.38
121.98
111.59
Cl
HN
O
NH
·HCl
N
N
H
NH2
11
f1 (ppm)

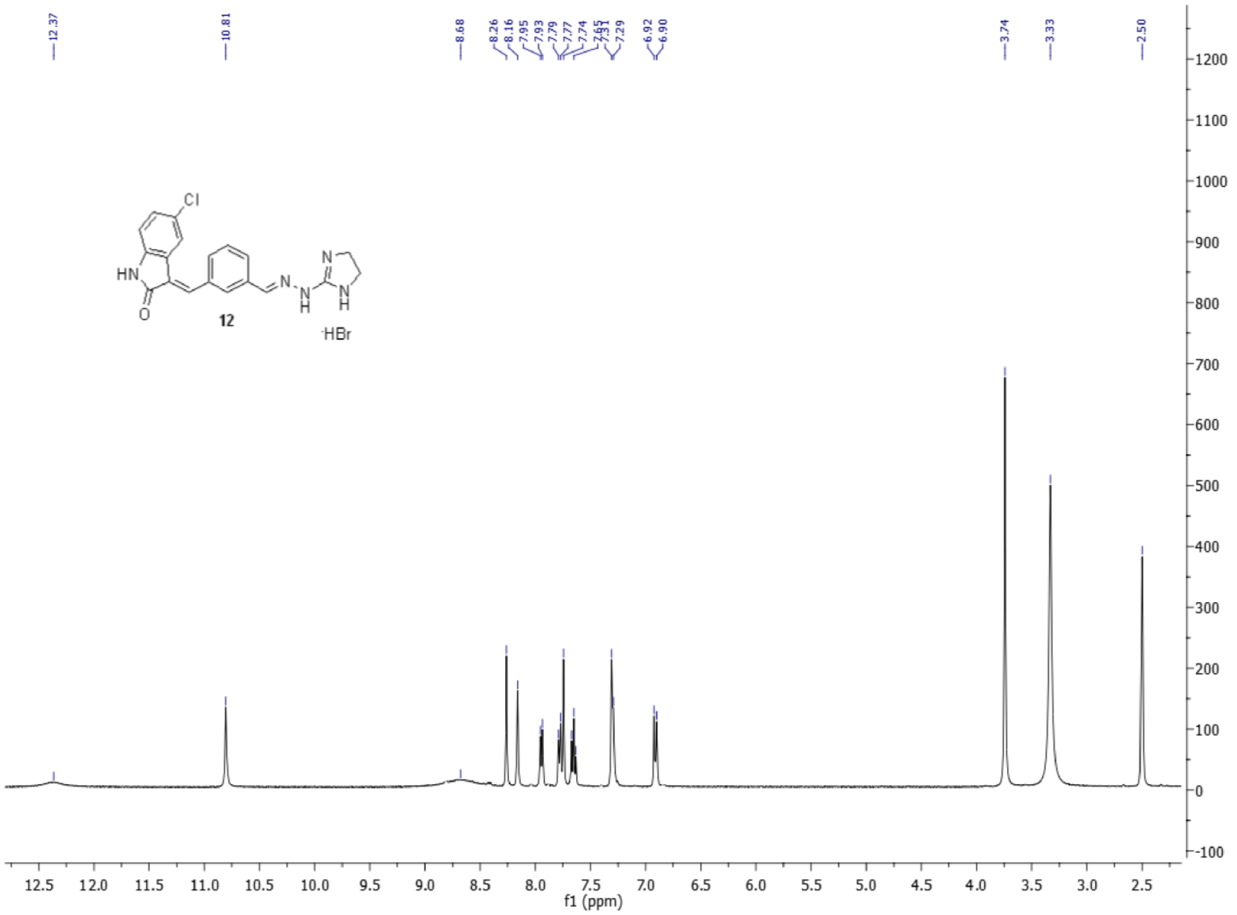
12.37
10.81
8.68
8.26
8.16
7.95
7.93
7.79
7.77
7.74
7.31
7.29
6.92
6.90
3.74
3.33
2.50
Cl
HN
O
N
N
H
N
H
12
·HBr
f1 (ppm)

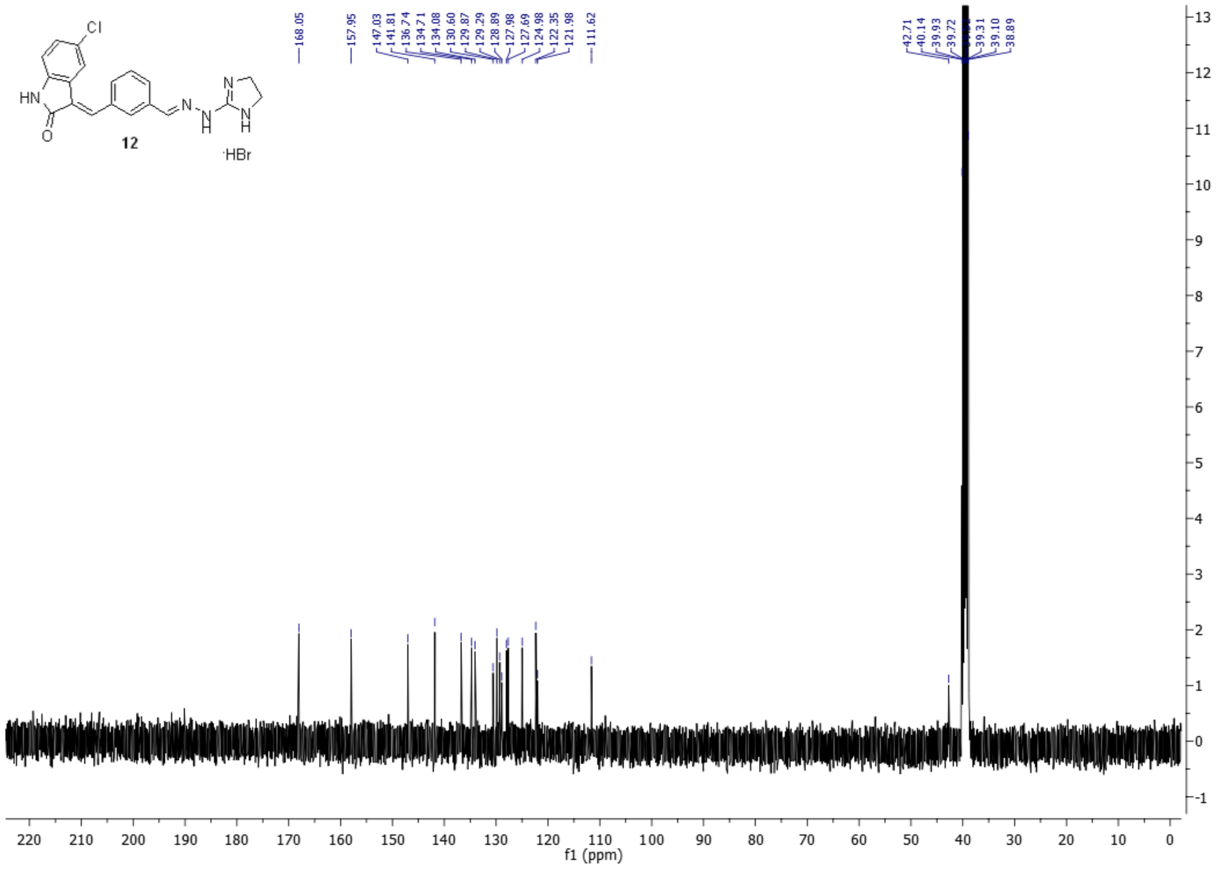
168.05
157.95
147.03
141.81
136.74
134.71
134.08
130.60
129.87
129.29
128.89
127.98
127.69
124.98
122.35
121.98
111.62
42.71
40.14
39.93
39.72
39.31
39.10
38.89
Cl
HN
O
N
N
H
N
H
12
·HBr
f1 (ppm)

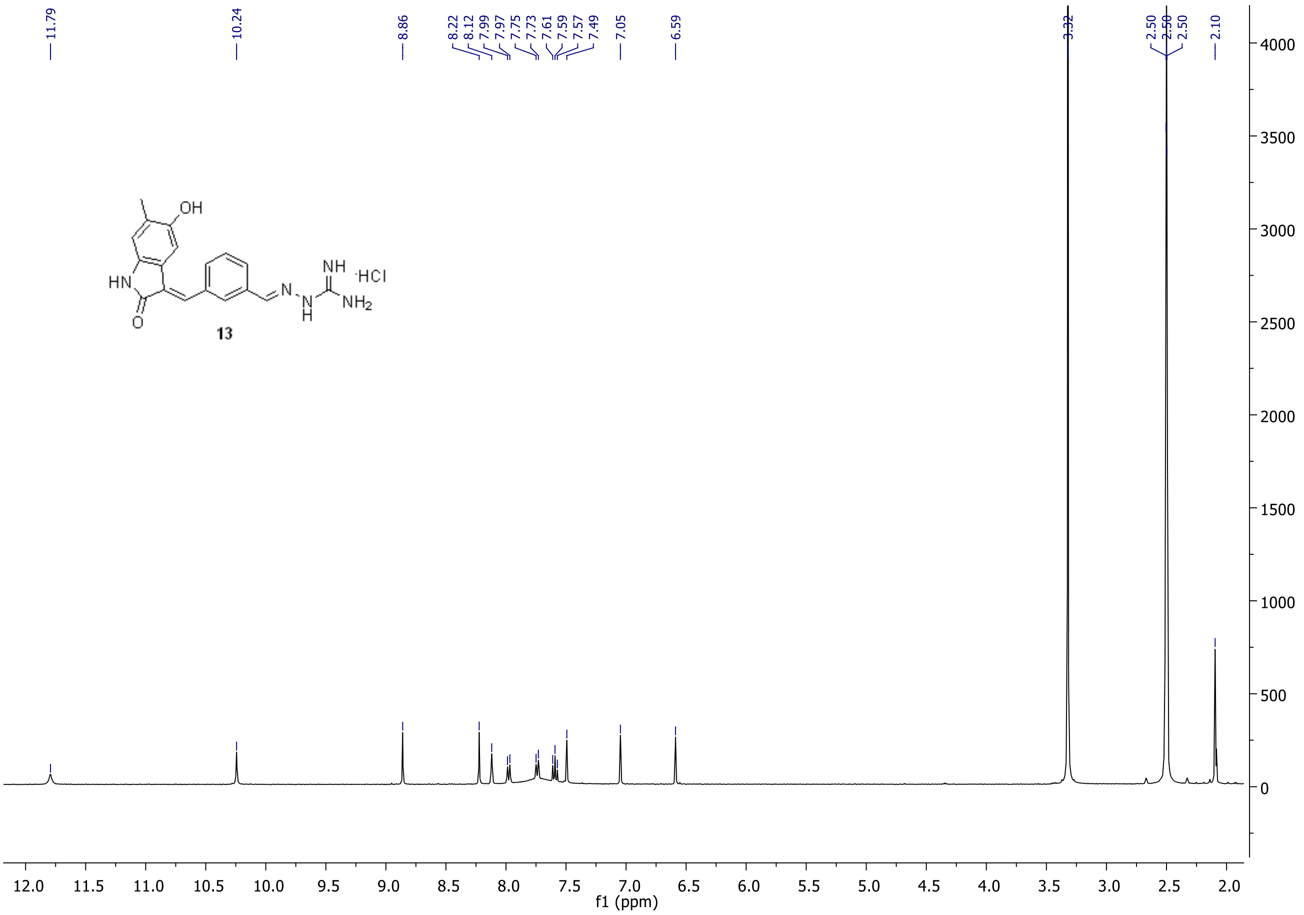
11.79
10.24
8.86
8.22
8.12
7.99
7.97
7.75
7.73
7.61
7.59
7.57
7.49
7.05
6.59
3.32
2.50
2.50
2.50
2.10
OH
HN
O
NH
·HCl
N
N
H
NH2
13
f1 (ppm)

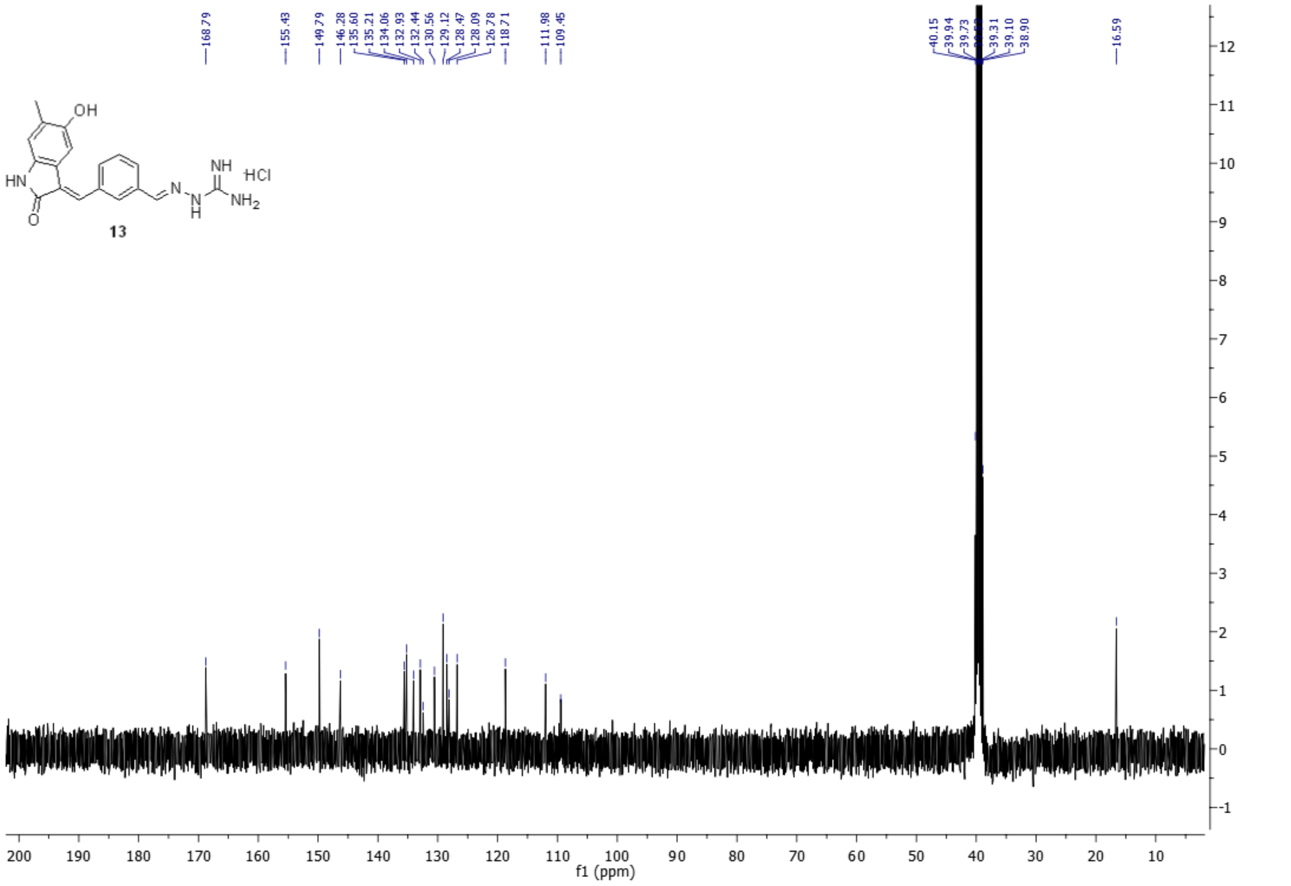
168.79
155.43
149.79
146.28
135.60
135.21
134.06
132.93
132.44
130.56
129.12
128.47
128.09
126.78
118.71
111.98
109.45
40.15
39.94
39.73
39.31
39.10
38.90
16.59
OH
HN
O
NH
·HCl
N
N
H
NH2
13
f1 (ppm)

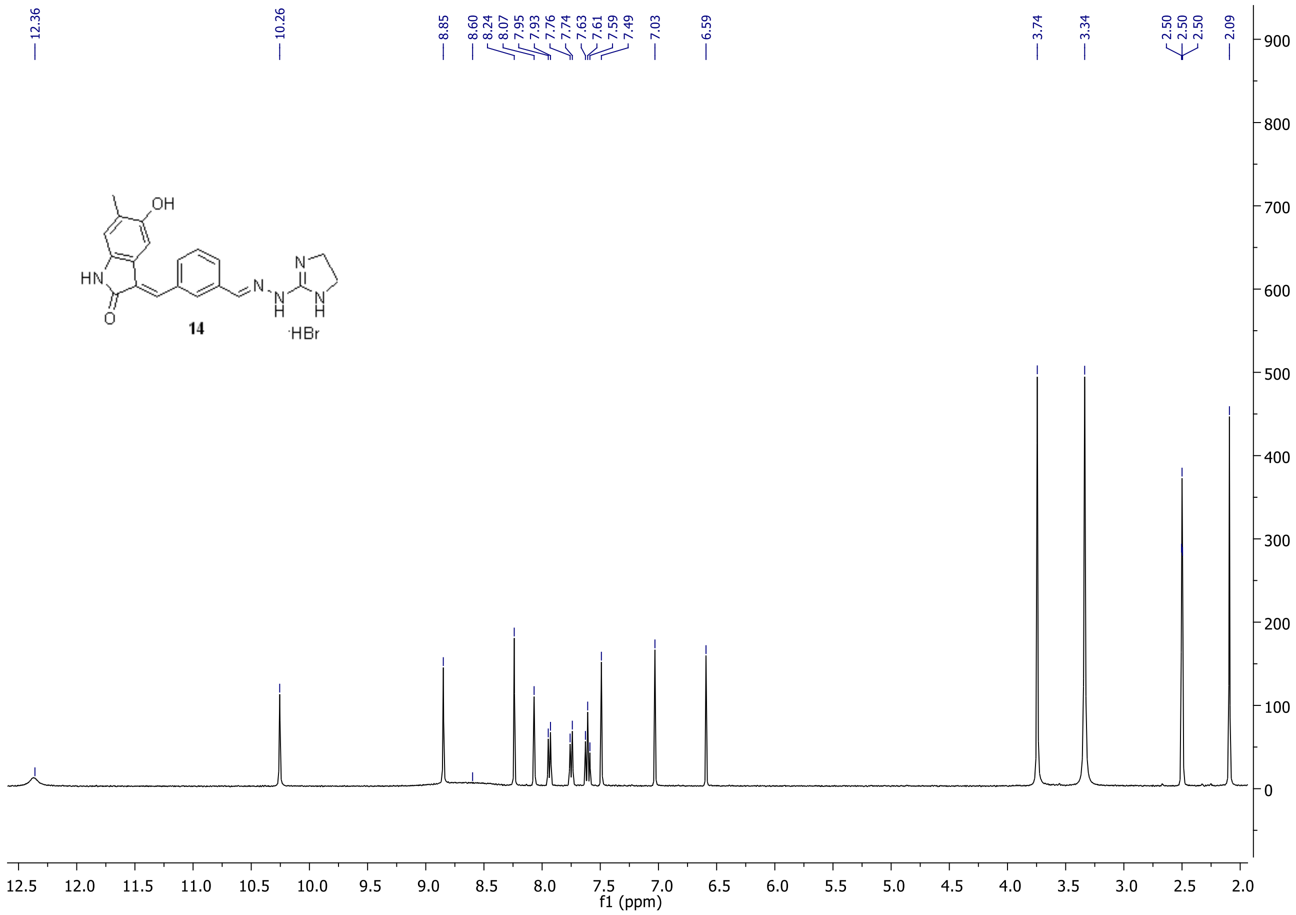
12.36
10.26
8.85
8.60
8.24
8.07
7.95
7.93
7.76
7.74
7.63
7.61
7.59
7.49
7.03
6.59
3.74
3.34
2.50
2.50
2.50
2.09
OH
HN
O
N
N
H
N
H
14
·HBr
900
800
700
600
500
400
300
200
100
0
12.5 12.0 11.5 11.0 10.5 10.0 9.5 9.0 8.5 8.0 7.5 7.0 6.5 6.0 5.5 5.0 4.5 4.0 3.5 3.0 2.5 2.0
f1 (ppm)

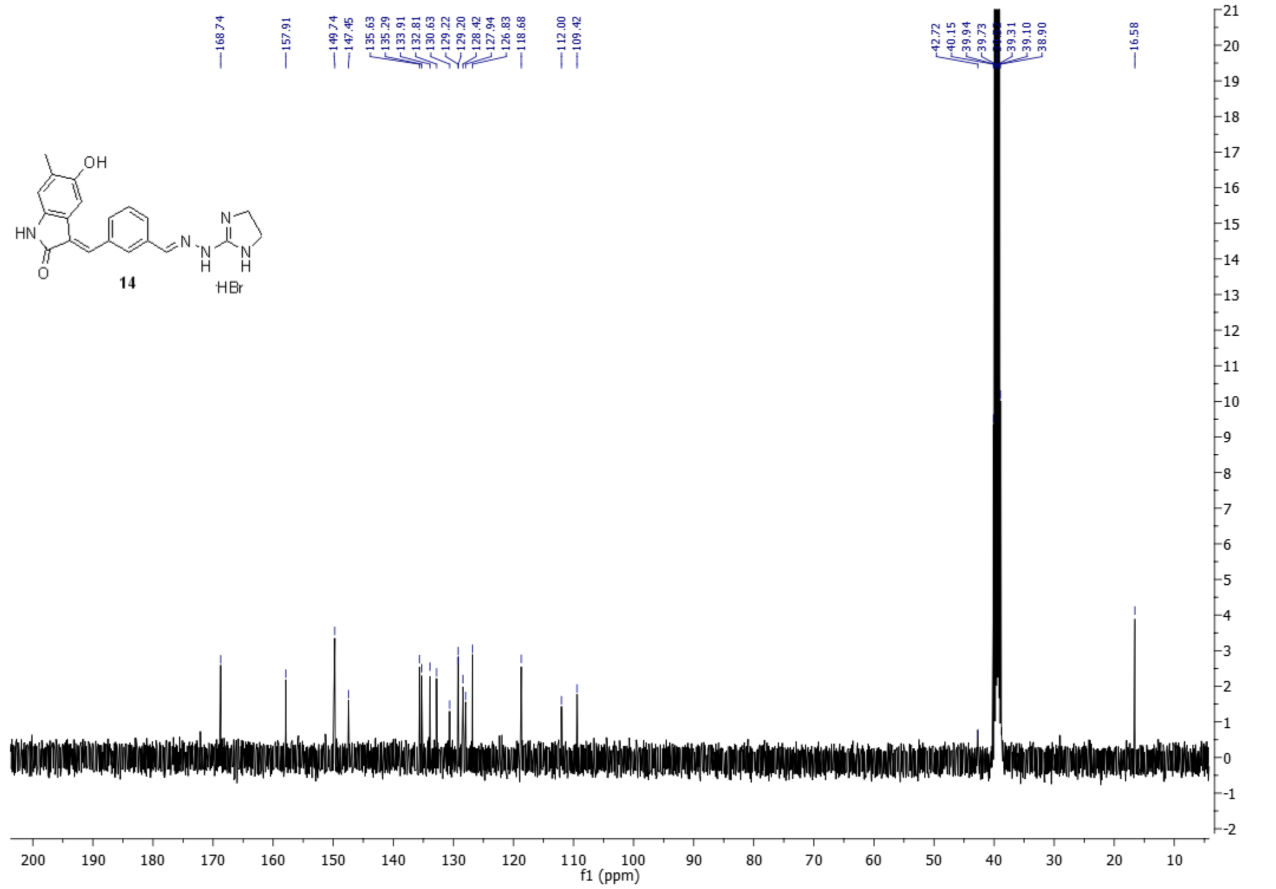
168.74
157.91
149.74
147.45
135.63
135.29
133.91
132.81
130.63
129.22
129.20
128.42
127.94
126.83
118.68
112.00
109.42
42.72
40.15
39.94
39.73
39.31
39.10
38.90
16.58
OH
HN
O
N
N
H
N
H
14
·HBr
21
20
19
18
17
16
15
14
13
12
11
10
9
8
7
6
5
4
3
2
1
0
-1
-2
200 190 180 170 160 150 140 130 120 110 100 90 80 70 60 50 40 30 20 10
f1 (ppm)

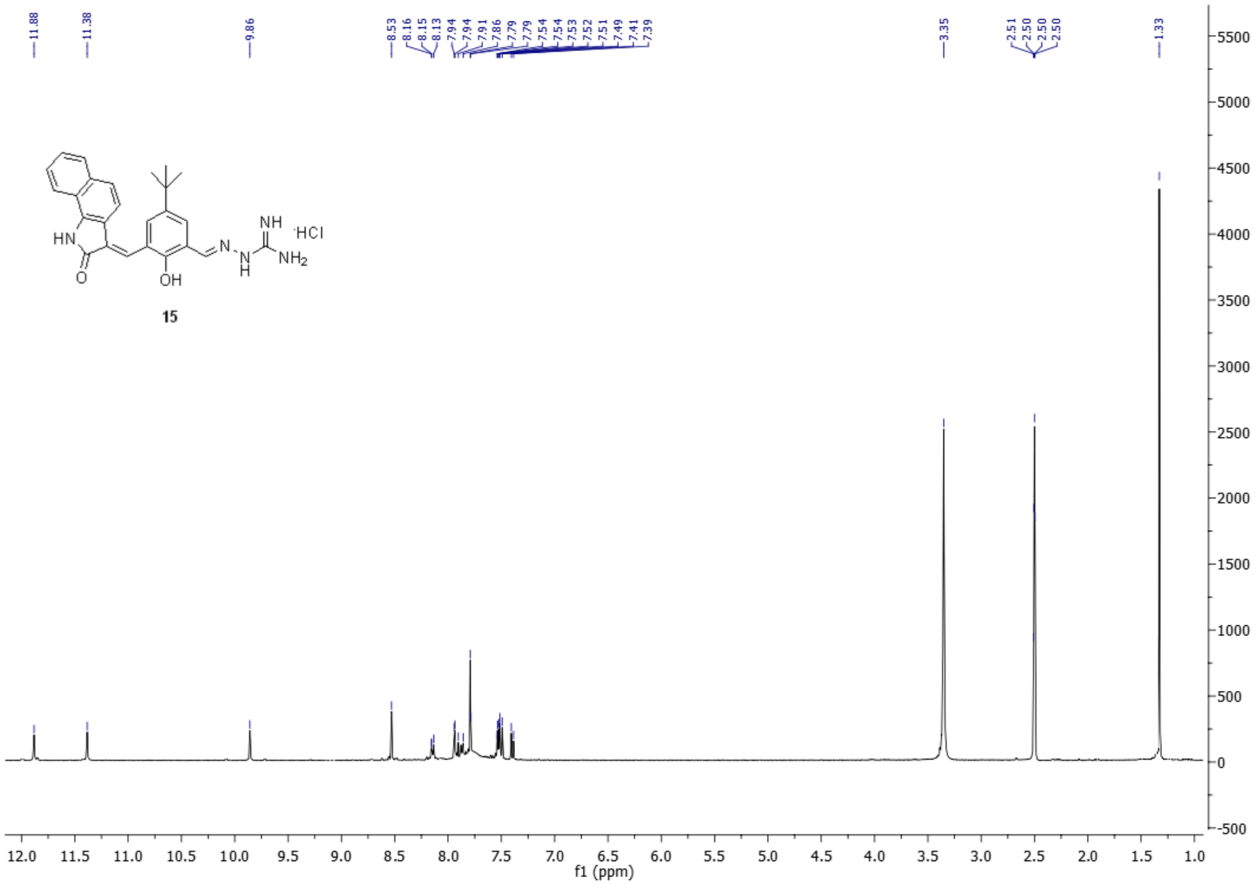
11.88
11.38
9.86
8.53 8.16 8.15 8.13 7.94 7.94 7.91 7.86 7.79 7.79 7.54 7.54 7.53 7.52 7.51 7.49 7.41 7.39
3.35
2.51 2.50 2.50 2.50
1.33
NH
·HCl
HN
NH2
O
OH
N
H
15
f1 (ppm)

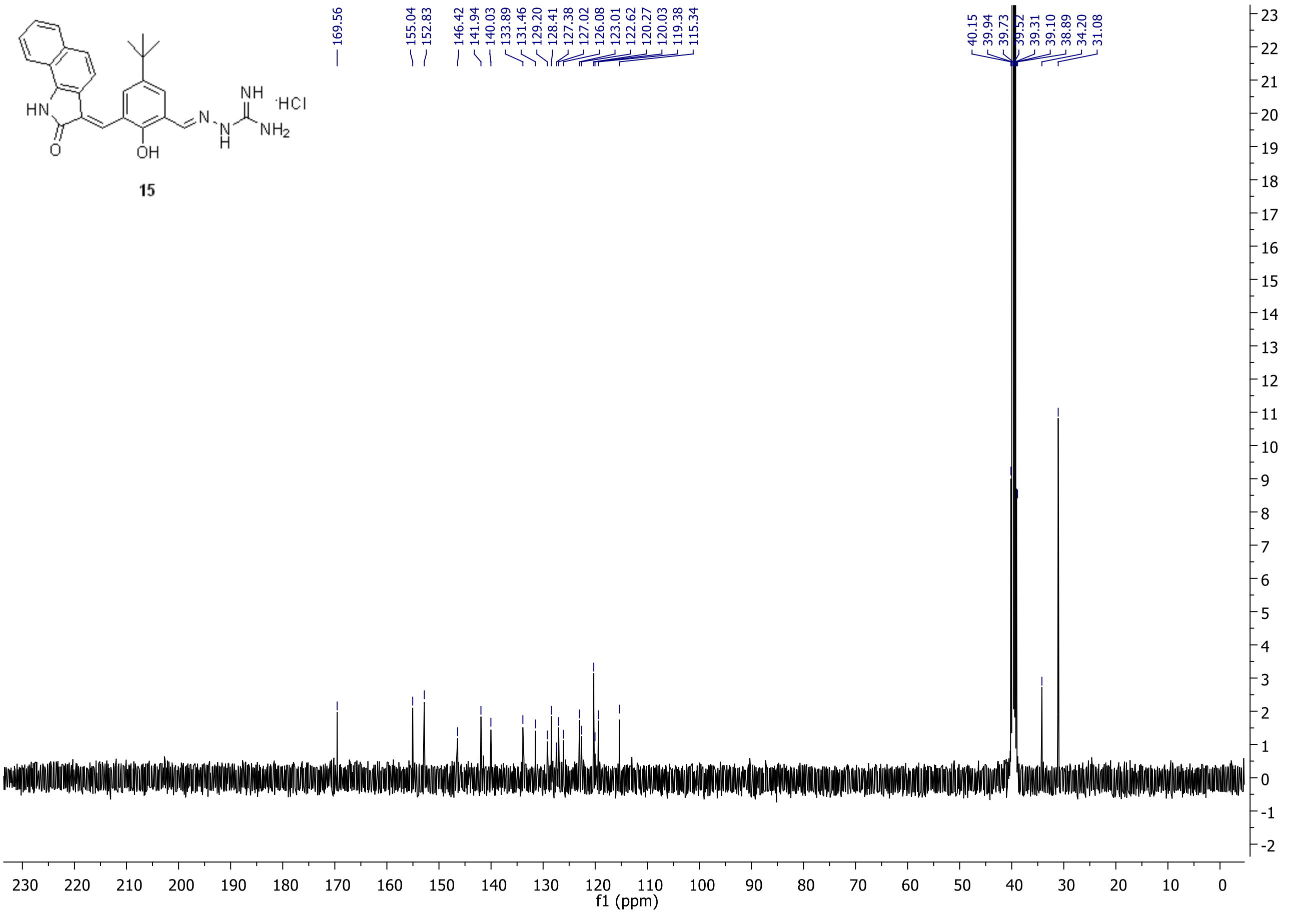
169.56
155.04 152.83
146.42 141.94 140.03 133.89 131.46 129.20 128.41 127.38 127.02 126.08 123.01 122.62 120.27 120.03 119.38 115.34
40.15 39.94 39.73 39.52 39.31 39.10 38.89 34.20 31.08
NH
·HCl
HN
NH2
O
OH
N
H
15
f1 (ppm)

Table S1. Ligand-induced thermal stabilization (ΔT_m)[a] of G4 and duplex DNA molecules measured by CD melting experiments.

**Compd**	***c-Kit1***	***c-Kit2***	***c-Myc***	***Tel$_{23}$-p***	***Tel$_{23}$-h***	***Hairpin***
**1**	4.0	7.0	5.4	6.5	1.0	1.0
**2**	5.5	10.5	8.6	7.7	1.5	3.5
**3**	9.5	17.5	12.5	14.5	3.5	4.5
**4**	10.5	23.5	ND[b]	16.1	4.0	7.0
**5**	5.0	9.0	9.0	26.5	2.5	0.0
**6**	5.0	14.0	7.6	9.0	3.1	2.0
**7**	3.0	10.5	7.0	19.5	4.0	2.5
**8**	2.0	7.0	6.9	15.5	2.5	0.0
**9**	12.0	24.0	ND[b]	22.5	7.5	4.0
**10**	14.5	26.0	ND[b]	26.0	5.5	3.0
**11**	11.0	20.0	12.9	26.5	5.0	4.0
**12**	13.5	20.5	14.1	22.0	7.0	3.6
**13**	8.5	21.0	14.0	22.3	10.0	6.0
**14**	9.0	18.5	14.0	17.5	9.0	0.5
**15**	1.5	10.5	8.0	27.0	1.0	1.0

[a]ΔT_m represents the difference in melting temperature [$\Delta T_m = T_m$ (DNA + 10 ligand equivalents) - T_m (DNA)]. The error on ΔT_m values is ± 1.0 °C. The T_m values of DNA molecules alone in 5 mM potassium phosphate buffer, pH 7.0, containing 20 mM KCl are: *c-Kit1* = 54.5 ± 0.5 °C; *c-Kit2* = 59.5 ± 0.5 °C; *c-Myc* = 77.6 ± 0.5 °C; *Tel$_{23}$-p* = 66.0 ± 0.5 °C; *Tel$_{23}$-h* = 55.0 ± 0.5 °C; *Hairpin* = 62.0 ± 0.5 °C. [b]These compounds increase significantly the thermal stability of the G4 (ΔT_m >22 °C); however, ΔT_m values are not determinable.

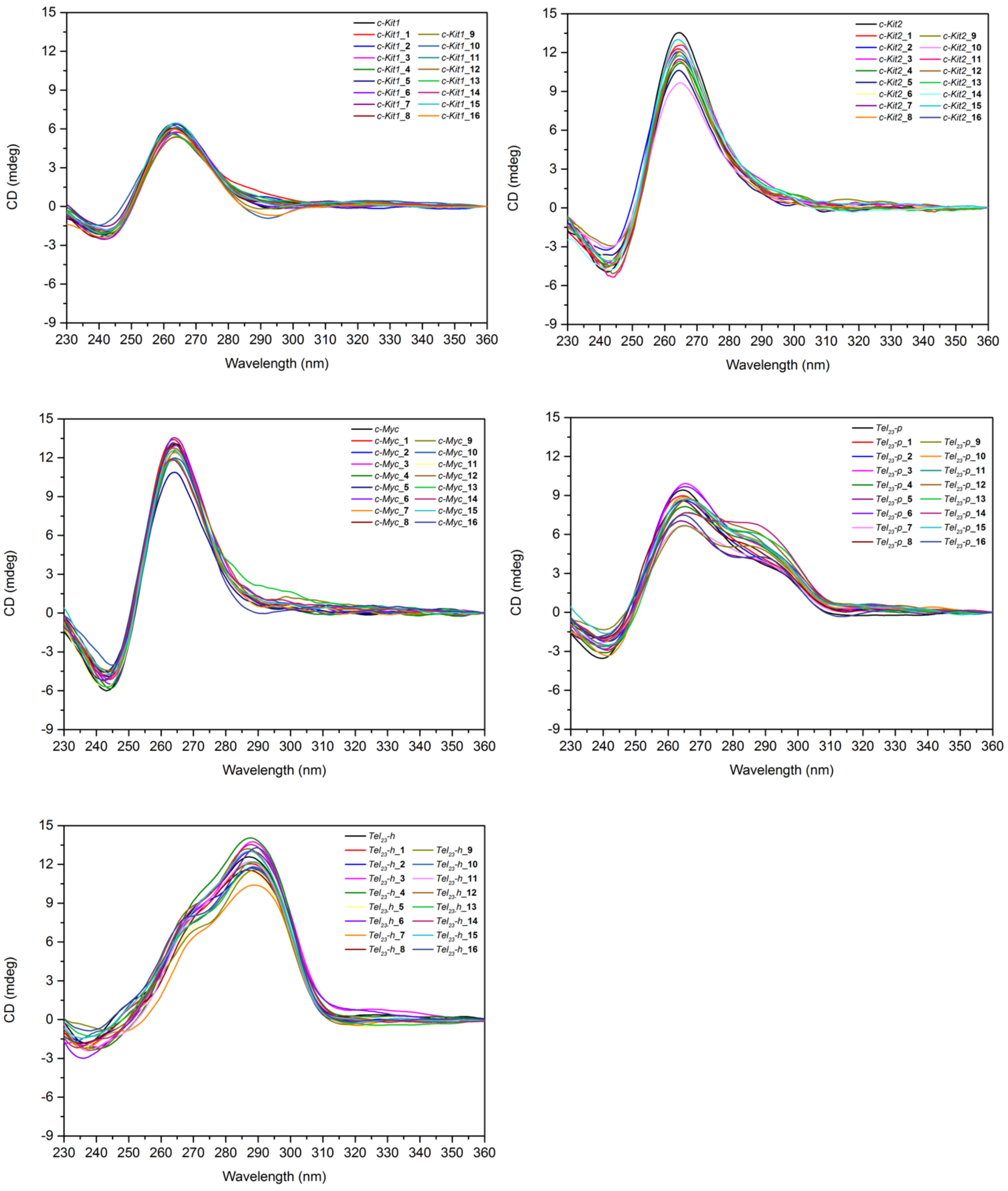

Figure S1. Circular dichroism spectra of the investigated G4s (15 μM) in the absence and presence of 10 mol. equiv. of compounds **1-15**.

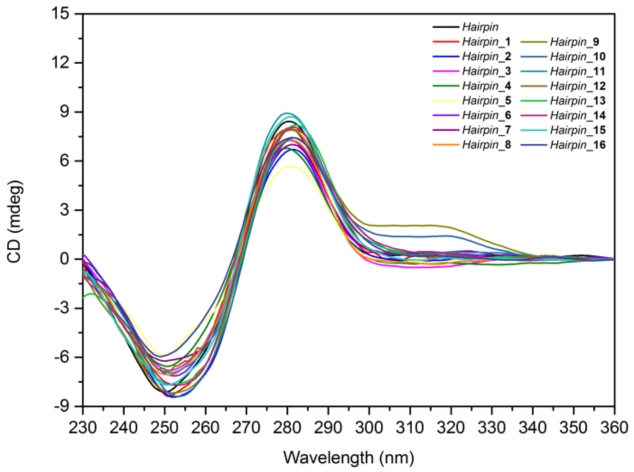

Figure S2. Circular dichroism spectra of the hairpin-duplex DNA (*Hairpin*) (30 µM) in the absence and presence of 10 mol. equiv. of compounds **1-15**.

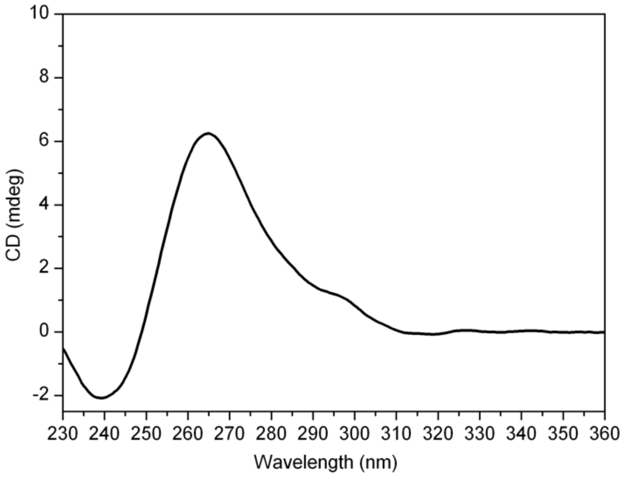

Figure S3. Circular dichroism spectrum of *F-Tel$_{21}$-T* (1 µM) in 20 mM potassium phosphate buffer (pH 7.0) containing 5 mM KCl, prepared by dilution from the highly concentrated (10 mM) DNA solution in annealing.

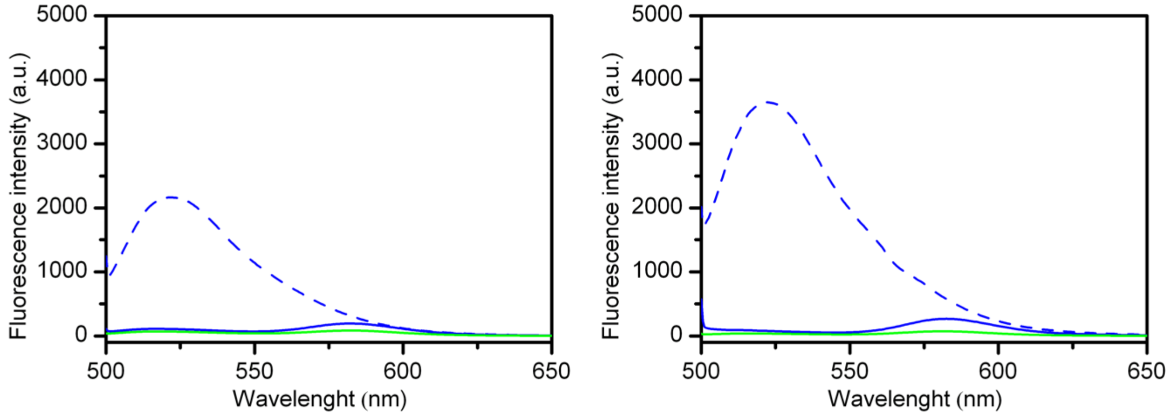

Figure S4. Fluorescence spectra of *F-Tel$_{21}$-T-p* (left panel) and *F-ckit2-T* (right panel) alone at 5 °C (blue line) and 100 °C (dashed blue line), and in the presence of 10 mol. equiv. of compound **15** at 5 °C (green line).

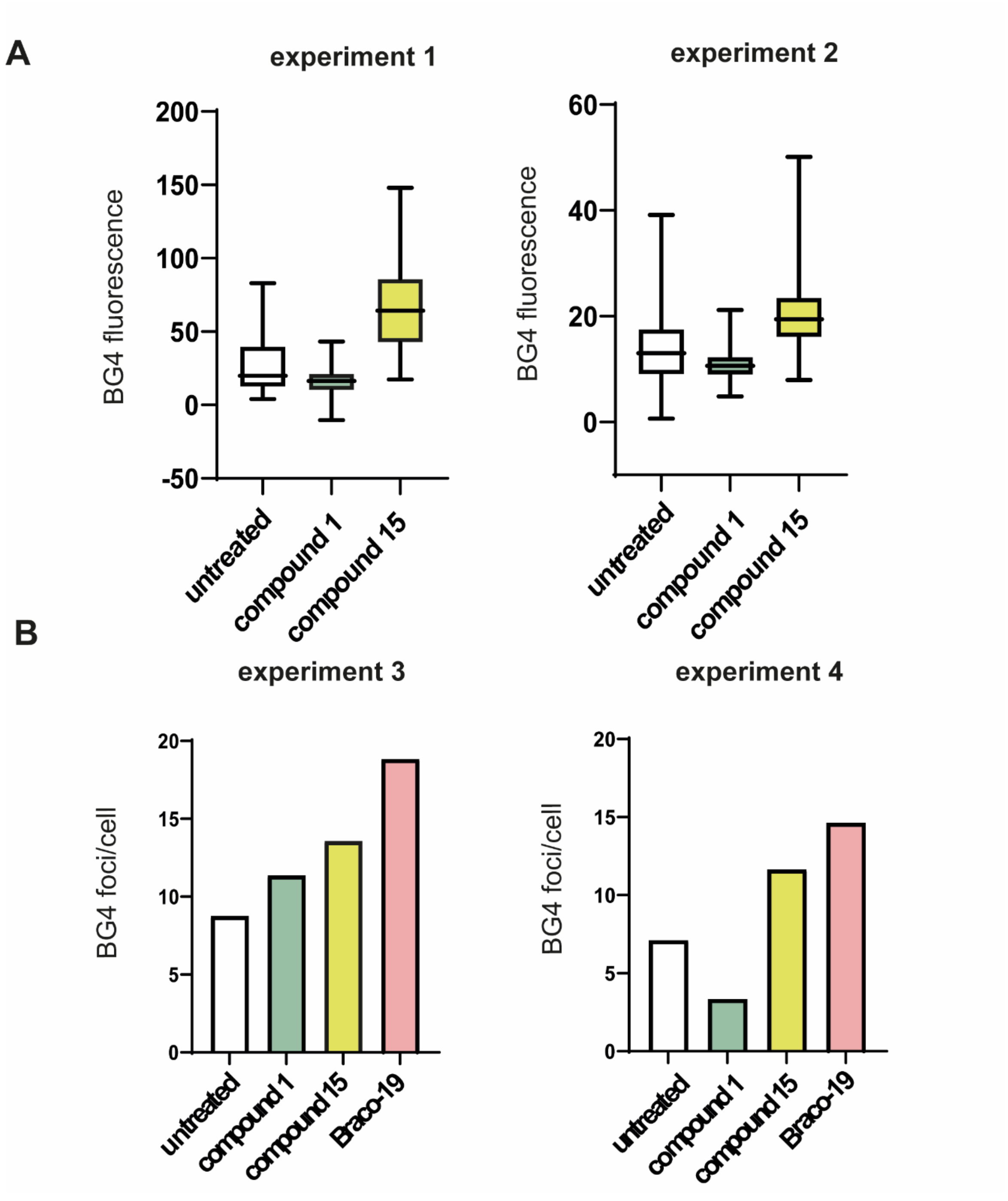

Figure S5. G4 stabilization induced by ligands at 24 h of treatment in U2OS cancer cells. (**A**) Raw data of BG4 fluorescence quantification after 24 h of treatment with compounds **1** (10 µM) and **15** (2 µM) in two biological replicates. (**B**) Raw data of BG4 foci quantification after 24 h of treatment with compounds **1** (10 µM), **15** (2 µM) and Braco-19 (10 µM) in U2OS cells in two biological replicates.

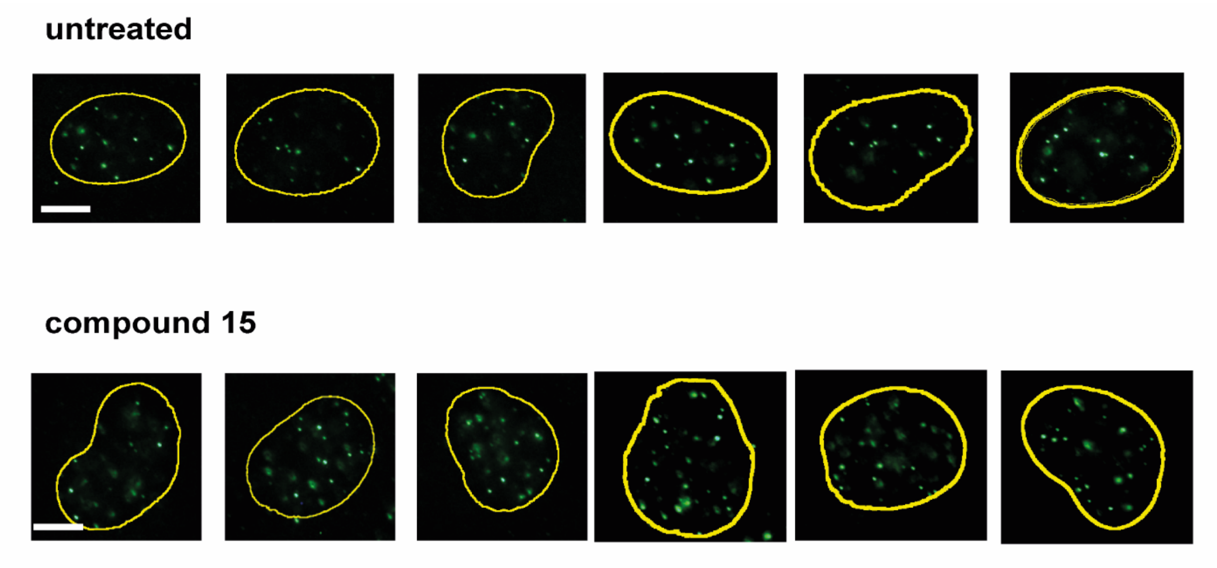

Figure S6. G4 stabilization by 24 h of treatment with compound **15**. Representative images of BG4 foci in untreated cells (top panel) and in cell after 24 h of treatment with **15** (bottom panel). Scale bar is 10 μm.

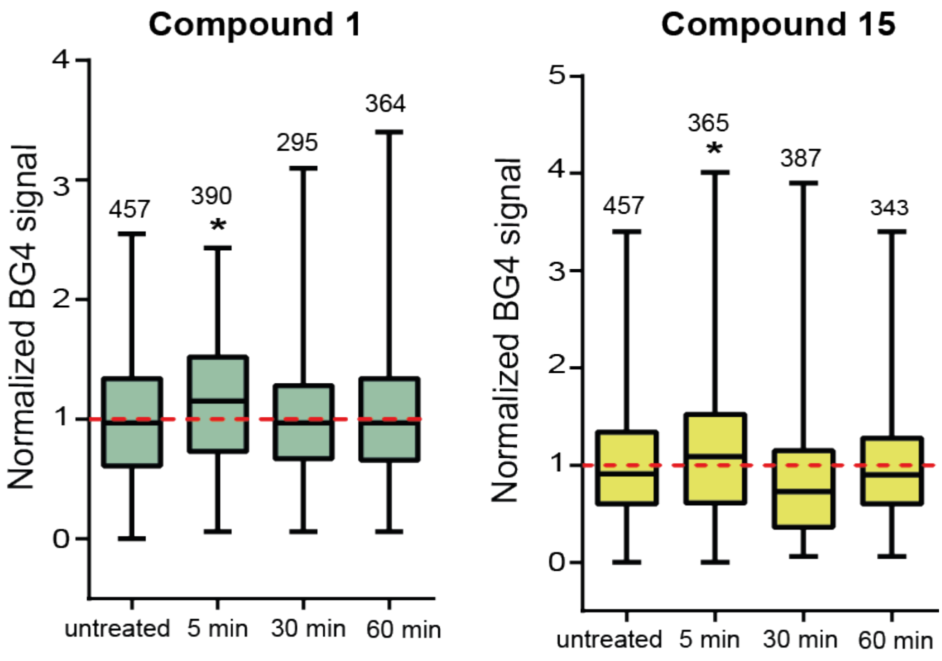

Figure S7. BG4 foci levels quantification in human U2OS cells induced by compounds **1** and **15** after short time of treatments. BG4 foci quantification with **1** (10 μM) and **15** (10 μM) after 5, 30 and 60 min of treatment. Values are means +/- SEM of two biological replicates. Significance has been evaluated by Kologorov-Smirnov parametric test: * $p<0.05$; ** $p>0.01$; *** $p>0.001$; **** $p<0.0001$ with GraphPad software. Numbers above box plot indicate cells nuclei analyzed. Graphs shows three biological replicates.

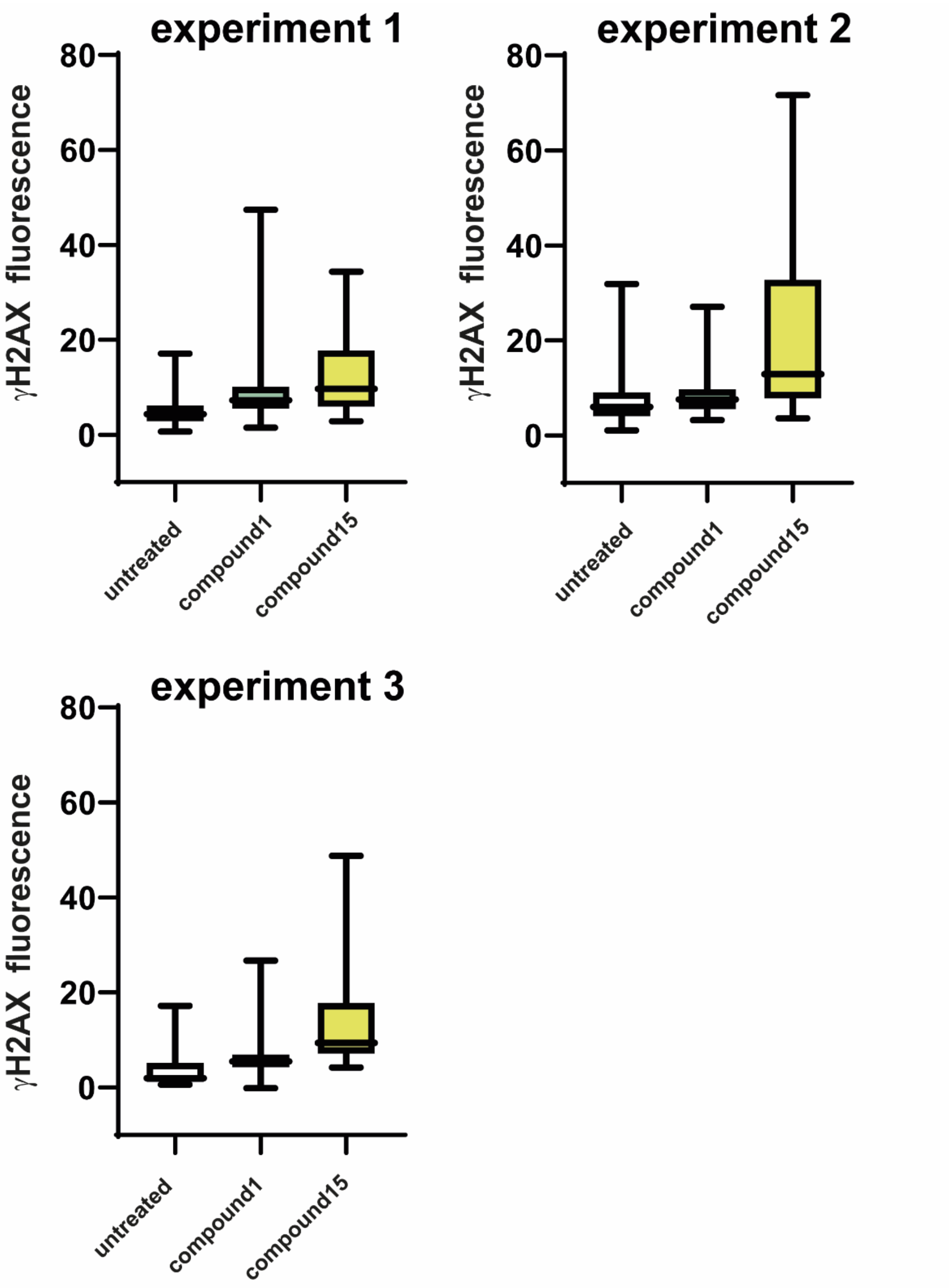

Figure S8. γH2AX fluorescence induced after 4 h of treatment with compounds **1** and **15** (10 µM) in U2OS cancer cells. Each of the 3 graphs shows a biological replicate.
